# Design, Development and Immunogenicity Study of a Multi-Epitope Vaccine Prototype Against SARS-CoV-2

**DOI:** 10.3390/ph17111498

**Published:** 2024-11-07

**Authors:** Mariyana Atanasova, Ivan Dimitrov, Nikola Ralchev, Aleksandar Markovski, Iliyan Manoylov, Silviya Bradyanova, Nikolina Mihaylova, Andrey Tchorbanov, Irini Doytchinova

**Affiliations:** 1 Drug Design and Bioinformatics Laboratory, Faculty of Pharmacy, Medical University of Sofia, 1000 Sofia, Bulgaria; matanasova@pharmfac.mu-sofia.bg (M.A.); idimitrov@pharmfac.mu-sofia.bg (I.D.); 2 Department of Immunology, Stefan Angelov Institute of Microbiology, Bulgarian Academy of Sciences, 1113 Sofia, Bulgaria; nikola_ralchev@microbio.bas.bg (N.R.); ammarkovsk@uni-sofia.bg (A.M.); iliyanmanoylov@microbio.bas.bg (I.M.); silvybradyanova@microbio.bas.bg (S.B.); mihaylova_n@microbio.bas.bg (N.M.)

**Keywords:** multi-epitope vaccine, SARS-CoV-2, humanized-ACE2 transgenic mice, molecular docking, machine learning methods, H2-Db, H2-Kb, I-Ab, peptide epitopes

## Abstract

**Objectives:** SARS-CoV-2 caused the COVID-19 pandemic, which overwhelmed global healthcare systems. Over 776 million COVID-19 cases and more than 7 million deaths were reported by WHO in September 2024. COVID-19 vaccination is crucial for preventing infection and controlling the pandemic. Here, we describe the design and development of a next-generation multi-epitope vaccine for SARS-CoV-2, consisting of T cell epitopes. **Methods:** Immunoinformatic methods were used to derive models for the selection of MHC binders specific for the mouse strain used in this study among a set of human SARS-CoV-2 T cell epitopes identified in convalescent patients with COVID-19. The immunogenicity of the vaccine prototype was tested on humanized-ACE2 transgenic B6.Cg-Tg(K18-ACE2)2Prlmn/J mice by in vitro, in vivo, and ex vivo immunoassays. **Results:** Eleven binders (two from the Envelope (E) protein; two from the Membrane (M) protein; three from the Spike (S) protein; and four from the Nucleocapsid (N) protein) were synthesized and included in a multi-epitope vaccine prototype. The animals were immunized with a mix of predicted MHC-I, MHC-II, or MHC-I/MHC-II peptide epitopes in Complete Freund’s Adjuvant, and boosted with peptides in Incomplete Freund’s Adjuvant. Immunization with SARS-CoV-2 epitopes remodeled the lymphocyte profile. A weak humoral response and the significant production of IL-4 and IFN-γ from T cells were found after the vaccination of the animals. **Conclusions:** The multi-epitope vaccine prototype presented in this study demonstrates immunogenicity in mice and shows potential for human vaccine construction.

## 1. Introduction

Severe acute respiratory syndrome coronavirus 2 (SARS-CoV-2) has caused the rapidly progressing pandemic of Coronavirus-disease 2019 disease (COVID-19), overwhelming healthcare systems worldwide. SARS-CoV-2 belongs to the β-coronavirus genus, which includes SARS-CoV, which emerged in 2002, and Middle Eastern Respiratory Syndrome Coronavirus (MERS-CoV), which emerged in 2012. This genus causes severe human diseases of zoonotic origin [1]. As of September 2024, the World Health Organization has reported more than 776 million cases of COVID-19 worldwide and over 7 million deaths [2]. The clinical manifestations of COVID-19 vary broadly, ranging from asymptomatic infection to acute respiratory failure and death, yet the underlying mechanisms for this high variability are still unknown. Similarly, the role of host immune responses in the viral clearance of COVID-19 remains unresolved, as the vast body of data available is highly controversial regarding the effectiveness of the humoral or cellular immune response in this infection.

**Figure 1:**
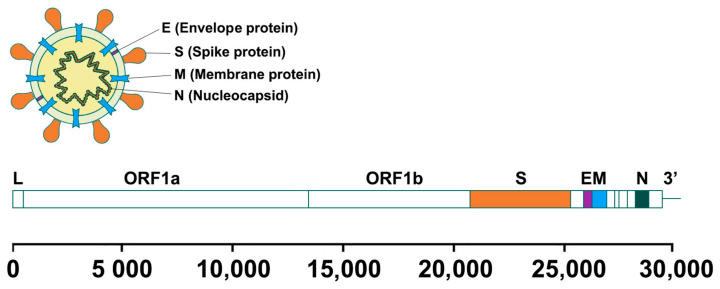
SARS-CoV-2 structure and genome.

SARS-CoV-2 has 12 putative functional open reading frames (ORFs) and shares an 82% nucleotide homology with SARS-CoV. There are at least four structural proteins in SARS-CoV-2: Spike (S), Envelope (E), Membrane (M), and Nucleocapsid (N) (Figure 1). The trimer S protein is cleaved into the S1, containing the receptor binding domain, and S2 subunits. S2 is further cleaved into S2′ to form the viral fusion peptide. The S protein is pivotal for viral entry and is a neutralizing target, as it is in SARS-CoV and MERS-CoV. The S protein is a key target for diagnostic tests and vaccine development as well. The S1 subunit of SARS-CoV-2 shares about 70% identity with SARS-CoV, whereas the sharred identity of the S2 subunit is up to 99%, with some evidence of cross-reactivity between the viruses. Apart from these structural proteins, the SARS-CoV-2 genome encodes for around 20 putative non-structural proteins [3]. Encouragingly, reports have shown that after infection with SARS-CoV-2, patients develop neutralizing antibody responses [4]. However, the magnitude of the antibody responses appears related to the clinical severity of the COVID-19 disease. These instances of low or no antibody responses to traditional serological approaches may lead to an underestimation of asymptomatic and mild infections and threaten the success of a potential vaccine that targets the S protein alone.

Starting from the first identified genetic sequence (Wuhan-Hu1 strain), the SARS-CoV-2 virus has undergone multiple sequence changes based on genetic mutations, currently distributed as several Omicron variants circulating in the human population. Moving to the new variants, the virus seems to become more contagious, but it does not lead to more serious pathogenesis. The high number of deaths worldwide and the extreme effect on social life, healthcare systems, and the economy require the urgent development of a therapy against SARS-CoV-2 [5].

Vaccination is one of the most efficient preventive measures taken against life-threatening infectious diseases and has dramatically improved public health. According to WHO (September 2024), more than 210 candidate vaccines against SARS-CoV-2 are currently in various stages of development. Among these, at least 48 are in human trials, with approximately 10 in phase III [6]. Understanding the adaptive immunity to SARS-CoV-2 is important for vaccine design, interpreting COVID-19 pathogenesis, and the organization of pandemic control measures. The first steps for such an understanding are the ability to quantify the virus-specific CD4+ and CD8+ T cells. Such knowledge is of immediate relevance, as it would provide insights into the immunity against and pathogenesis of SARS-CoV-2 infection, and the same knowledge would assist vaccine design and the evaluation of candidate vaccines.

During the 2020 SARS-CoV-2 pandemic, billions of doses of different registered vaccines were administered to the world population. They differ in their principle of action, content, number of doses, immunogenicity, effectiveness, and side effects [7]. The worldwide distribution of the various vaccines was unevenly spread across regions, with a distinct effect on the level of pandemic and mortality. Among them, mRNA-based vaccines were administered to patients with the aim of preventing infection or severe disease from the current viral strain [8]. Regardless, people immunized with mRNA-based vaccines are still unprotected from the mutated strains of the virus, and the subvariants of the Omicron strain continue to spread infections all over the world. For this reason, the population must be vaccinated or receive secondary boosters with vaccines that are updated every year and are expected to be effective against the new virus strains.

Most of the exploited mRNA-based vaccines against SARS-CoV-2 elicit a predominantly humoral immune response with limited potential to induce cell-mediated antiviral processes. The core SARS-CoV-2 S protein is the leading viral antigen and shows great importance in the pathogenesis of the virus by inducing the generation of neutralizing antibodies during infection. This makes the S protein the logical target for SARS-CoV-2 vaccine development. Unfortunately, S protein-based mRNA vaccines cannot provide effective protection against genetically diverse viruses, which is caused by the high rate of viral mutations of SARS-CoV-2. Newly developed vaccines designed to enhance both T cell-mediated and antibody responses may contribute to effective immune protection [9,10].

T cell epitope-based vaccines have been tested and proven to be an effective way to activate T cell-mediated immune responses. Structural proteins contain many epitopes responsible for cellular immune responses and that are recognized by T and B cells. Instead of whole intact proteins or even attenuated pathogens used for vaccine design, multi-epitope vaccines consisting of conserved short peptide epitopes have the potential to elicit a strong and broad immune response to different SARS-CoV-2 strains. Both structural and non-structural viral proteins contain such short epitopes, which could be derived after antigen processing during the viral infection. These short peptide epitopes are involved in antigen presentation by the major histocompatibility complex (MHC) on the surface of antigen-presenting cells (APCs) and are recognized by T cells triggering cell-mediated immune responses. Such epitope-bearing peptides can be incorporated into epitope-based vaccines [9,10].

Through the use of HLA class I and II predicted peptide ‘megapools’, Grifoni et al. [11] were able to identify circulating SARS-CoV-2-specific CD8+ and CD4+ T cells in approximately 70% and 100% of COVID-19 convalescent patients, respectively. Robust CD4+ T cell responses to the Spike (S) protein, which is the primary target of most vaccine development efforts, were observed and were found to be correlated with the levels of anti-SARS-CoV-2 IgG and IgA antibodies. The Membrane (M), S, and Nucleocapsid (N) proteins made up 11–27% of the total CD4+ response, and additional responses targeted nsp3, nsp4, ORF3a, and ORF8, among others. The CD8+ T cells recognized Spike and M, with at least eight SARS-CoV-2 ORFs targeted. Notably, SARS-CoV-2-reactive CD4+ T cells were detected in approximately 40–60% of unexposed individuals, indicating the possibility of cross-reactive T cell recognition between circulating “common cold” coronaviruses and SARS-CoV-2.

The human leukocyte antigen (HLA) proteins contain a specific binding site for peptides of different origin, including viral. The binding site has a polymorphic architecture and determines the extreme polymorphism of HLA alleles. There are more than 27,000 HLA class I and 11,000 HLA class II alleles registered in the IPD-IMGT/HLA database (April 2024) [12]. The clinical outcome of viral infections is associated with the HLA genotype [13,14,15]. These HLA proteins, which make stable and long-living viral peptide complexes and enable T cell-based immunity development, are associated with mildly severe infection or even resistance to viral infection. On the contrary, HLA proteins failing to form a complex with viral peptides are associated with susceptibility to and severity of viral infection. Several associations between HLA and the current SARS-CoV-2 infection are already described [16,17,18,19].

The prediction of peptide binding to HLA molecules is crucial in immunology and drug design. Several computational methods have been developed to forecast peptide–HLA interactions, aiding in understanding immune responses and disease mechanisms [20,21]. Sequence-based algorithms like NetMHC, NetMHCpan, and NetCTL utilize experimental binding data to predict peptide–HLA binding affinities based on sequence motifs and HLA polymorphisms [22,23]. Structure-based approaches, employing tools like Rosetta [24] and AutoDock [25], analyze 3D structures to predict binding modes and interactions between peptides and HLA molecules. Data-driven machine learning models, including MHCflurry [26], and MixMHCpred [27,28], use deep learning on extensive binding datasets, capturing intricate peptide–HLA interactions for accurate predictions. Additionally, integrated approaches combining genomics, transcriptomics, and proteomics data enhance the prediction accuracy, considering individual variations in HLA expression and peptide processing pathways [29].

MHC/HLA class I binders are associated with T cell epitopes and activate CD8+ T cells (cytotoxic), which is important for killing infected cells. MHC/HLA class II binders are associated with T helper cells (CD4+) and indirectly help the production of antibodies by B cells. Our contribution in the field is focused on developing models for peptide binding prediction to HLA proteins based on docking-based quantitative matrices (QMs) and machine learning (ML) methods. The QMs for 23 HLA class II alleles are implemented in EpiDOCK server [30], while the sequence-based models are freely accessible via EpiJen [31] and EpiTOP servers [32]. Despite challenges, these methods collectively facilitate peptide–HLA binding predictions, crucial in vaccine design and understanding immune-related diseases.

The aim of the present study is the knowledge-based development of a next-generation novel multi-epitope vaccine for a preventive therapy for SARS-CoV-2. The multi-epitope vaccines consist of several HLA-restricted epitopes originating from viral proteins and recognizable by CD8+ or/and CD4+ T cells [33]. Here, we develop docking-based QMs and ML models to identify SARS-CoV-2 peptides binding to MHC proteins specific for the tested mouse strain. The peptides were identified that bind to mouse MHC class I molecules (H2-Db and H2-Kb) as well as mouse MHC class II molecules (I-Ab). The docking-based QMs, utilizing peptide–MHC 3D structures, decipher binding preferences at each peptide position within the binding core. Conversely, the ML models rely on the sequence analysis of peptides with known affinities for distinct mouse MHCs, constituting sequence-based predictors. Both model types undergo rigorous validation against external test sets. Based on their performance metrics, we select the models demonstrating the highest predictive accuracy. These models are subsequently employed to identify the most probable peptide binders from the main SARS-CoV-2 structural proteins—S, M, N, and Envelope (E). The peptide binders are included in a multi-epitope vaccine prototype. The vaccine is expected to generate a virus-specific T and/or B cell immune response and to serve as in vivo validation of the protective properties of a nanoparticle-based multi-epitope vaccine tailored to be active on humanized-ACE2 transgenic B6.Cg-Tg(K18-ACE2)2Prlmn/J mice.

## 2. Results

The vaccine prototype developed in the present study is tested on transgenic B6.Cg-Tg(K18-ACE2)2Prlmn/J mice. The transgenic mouse model is developed by the insertion of the human ACE2 gene into the genome of C57B6 mice. This mouse strain carries the MHC class I alleles H2-Db and H2-Kb and the MHC class II allele I-Ab. The multi-epitope vaccine is designed to contain peptides binding to these MHC alleles.

### 2.1. Development of Docking-Based Models for the Structure-Based Vaccine Design

The input data for the structure-based vaccine design include the X-ray structures of the complexes formed between the peptides and MHC proteins H2-Db (pdb code: 4L8D) [34], H2-Kb (pdb code: 3P9L) [35], and I-Ab (pdb code: 1MUJ) [36]. The 3D structures were retrieved from the Protein Data Bank (https://www.rcsb.org, accessed on 28 June 2021) [37]. The peptides in the complexes were used as parent structures for the construction of combinatorial libraries through the method of single amino acid substitution (SAAS), where each position of the peptide-binding core was sequentially replaced by every other amino acid. Each substitution results in the generation of a new peptide. The combinatorial peptide library for H2-Db included 172 peptides (19 aas × 9 positions + 1 parent peptide), the library for H2-Kb included 153 peptides (19 × 8 + 1), and that for I-Ab included 267 peptides (19 × 14 + 1).

The molecular docking in the present study was performed by AutoDock Vina [38] using the settings given in Table S1. The protocols were applied on the combinatorial libraries for each mouse MHC allele. The best binding pose (with the lowest energy of binding) for each peptide was collected and normalized in three different modes: normal score, normal score per unit mass including all hydrogen atoms, and normal score per unit mass including polar hydrogens only. Thus, three docking-based quantitative matrices (QMs) were generated for each allele. The normalized values reflect the contribution of each amino acid at each position in the binding core to the overall binding affinity. A positive value indicates that a particular amino acid at a given position enhances the binding affinity, while a negative value indicates a decrease in affinity.

### 2.2. Development of Regression and Machine Learning Models for the Sequence-Based Vaccine Design

Three sets of peptides of different length binding to H2-Db, H2-Kb, and I-Ab were collected from the Immune Epitope Database (IEDB) (https://www.iedb.org, accessed on 25 September 2024) [39]. The initial set for H2-Db included 29,328 records, the set for H2-Kb included 27,464 records, and that for I-Ab included 7798 records. Each record contains a peptide sequence, test method, type of assay, and quantitative and qualitative evaluation of the binding. Peptides containing non-natural amino acids were removed. Among the records, the peptides with a dissociation constant *K_d_*, measured by the competitive radioactivity method on purified MHC, were the most common and they were collected and allocated to three sets. The set for the H2-Db allele contained 1902 peptides, that for H2-Kb contained 2219, and that for I-Ab contained 711. The *K_d_* values were presented as negative decimal logarithms (p*K_d_*). Duplicates and peptides with uncertain *K_d_* values were removed and the sets further decreased to 1384, 2006, and 571 peptides, respectively. Among the peptides binding to H2-Db and H2-Kb, 690 nonamers and 880 octamers were collected as training sets, respectively. Each peptide binding to I-Ab was presented as a set of overlapping nonamers. The training sets were used for model development.

Additionally, five external test sets for each allele were collected from IEDB. Set A contained peptides binding to the corresponding MHC with affinities measured using the fluorescence method with cellular MHC. Set B included binding peptides measured by mass spectrometry of cellular MHC. Set C consisted of binders measured by mass spectrometry of secreted MHC. Set D comprised binders derived by mass spectrometry or the fluorescence method. Set N included non-binders measured either by the fluorescence or radioactivity method. The external test sets were used for the validation of the models derived from both the structure- and sequence-based analyses. The quantity of peptides within the datasets used in the sequence-based analysis is summarized in Table S2.

The peptide sequences in the study were encoded by amino acid descriptors. Two types of descriptors were used: binary strings and *z* scores. Each binary string consists of 20 elements: 19 zeros and 1 unit. The position of the unit corresponds to the amino acid order, as follows: Ala, Arg, Asn, Asp, Cys, Gln, Glu, Gly, His, Ile, Leu, Lys, Met, Phe, Pro, Ser, Thr, Trp, Tyr, Val. Three *z* scores (*z_1_*, *z_2_*, and *z_3_*) describing the main amino acid properties were used. These descriptors have been obtained by Hellberg et al. through a principal component analysis (PCA) conducted on a matrix encompassing 29 physicochemical properties attributed to the twenty naturally occurring amino acids [40]. The first principal component, designated as *z_1_*, is associated with hydrophobicity; the second component, *z_2_*, characterizes molecular size; and the third component, *z_3_*, represents electronic properties.

A multiple linear regression (MLR) with partial least squares (PLS) was applied on the peptide training sets encoded as binary strings and by *z* scores using SIMCA 13.0 (Sartorius AG, Göttingen, Germany). The coefficients were presented in three formats: centered and scaled (CS), MLR, and unscaled. The ML methods used in the study were the radial basis function network (RBF), multilayer perceptron (MLP), *k* nearest neighbor (kNN), random forest (RF), support vector machine (SVM), Gaussian Naïve Bayes (GaussianNB), and Xgboost, as implemented in Weka 3-8-3 [41].

The models derived by regression and ML methods were validated by the external test sets, and their predictive ability was assessed by the measures of *sensitivity* (true binders/all binders) and *specificity* (true non-binders/all non-binders). As the PLS and RBF models predict continuous *pK_d_* values, their predictive ability was assessed at cutoffs between the binders and non-binders ranging from 5.3 (−log*K_d_* = 5000 nM) to 6.3 (−log*K_d_* = 500 nM) with a step of 0.1.

The QMs, generated through either the docking-based approach or PLS method, served to evaluate the binding affinity of any given peptide by summarizing the contributions (coefficients) of amino acid residues at each position within the peptide-binding core. Peptides were classified as binders if the predicted affinity surpassed a predefined threshold; otherwise, they were classified as non-binders.

### 2.3. Selection of Models for Peptide Binding Prediction to H2-Db

In the structure-based analysis, three docking-based QMs for H2-Db were generated. In the sequence-based analysis, 8 PLS and 14 ML models were derived. Half of them were developed on the binary coded sets, the other half was developed on the sets encoded by *z* scores. The models were validated by the external test sets. The predictive ability values assessed by the parameters *sensitivity* and *specificity* of the best-performing structure- and sequence-based models are given in Table S3.

The docking-based QMs gave poor predictions, with *sensitivity* and *specificity* for some of the test sets below 50%. The poor performance of the docking method likely stems from a trade-off between the fixed backbone assumed for the binding peptide during docking onto the mouse MHCs and the computational time required for accurate calculations. Among the sequence-based models, the best-performing PLS, RBF, and RF models showed *sensitivities* in the range 74–88% and *specificities* between 60% and 71%. The number of principal components (PCs) in the PLS models was three. Further, these three models were used to select the high H2-Db binders for the multi-epitope vaccine prototype.

**Figure 2:**
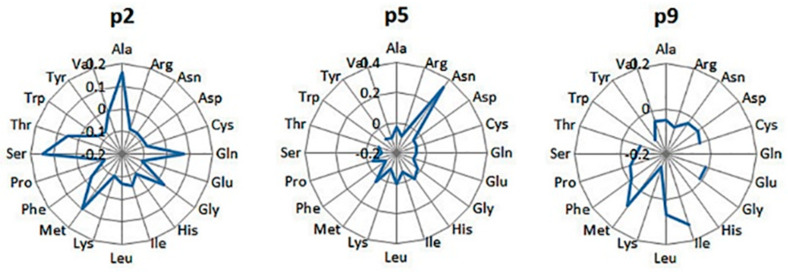
Contributions of the amino acids at the anchor positions 2, 5, and 9 in the peptide binding to H2-Db, according to the PLS model. Peptides with Trp at p5 and with Gln, His, and Trp at p9 are absent in the training set.

The preferred amino acids at the anchor positions p2, p5, and p9 [34], according to the PLS model, are given in Figure 2. At p2, Ala, Ser, Met, Gln, Thr, and Gly are preferred; Pro and Glu are deleterious. At p5, Asn is favored; Phe and Trp are toxic. At p9, aliphatic residues like Ile, Met, and Leu are well accepted, while bulky or positively charged residues are unacceptable. Peptides with Trp at p5 and with Gln, His, and Trp at p9 are absent in the training set. These preferences are in good agreement with the binding motif and QM for H2-Db available in IEDB. The PLS QM for H2-Db is given in the Supplementary Materials (Table S4).

### 2.4. Selection of Models for Peptide Binding Prediction to H2-Kb

A total of 3 docking-based QMs, 8 PLS, and 14 ML models were generated for H2-Kb. The PLS models were based on two PCs. The results from the external validation of the best predictive models are given in Table S5.

Similarly to the docking-based QMs for H2-Db, the docking QMs for H2-Kb gave poor predictions. The best-performing sequence-based models were PLS (for the binders only), RBF, and RF with *sensitivities* ranging between 71% and 98% and *specificities* of 61% and 73% for the RF and RBF models, respectively. These three models for peptide binding to H2-Kb were used next in the present study to select high binders for the multi-epitope vaccine prototype.

**Figure 3:**
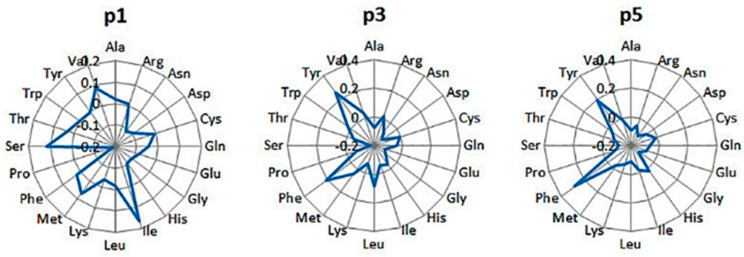
Contributions of the amino acids at the anchor positions 1, 3, and 5 in the peptide binding to H2-Kb, according to the PLS model.

The anchor positions for this allele are p1, p3, and p5 [33]. According to the PLS model, the preferences at p1 are for Ile, Ser, Val, and Met (Figure 3). At p3 and p5, the favored residues are Tyr and Phe. Ser, Asp, and Glu are deleterious at p3; Gly, Asn, and Ser are deleterious at p5. Again, the preferences for the anchor positions derived in the present study agree fully with the binding motif and QM for H2-Kb available in IEDB. The PLS QM for H2-Kb is given in the Supplementary Materials (Table S6).

### 2.5. Selection of Models for Peptide Binding Prediction to I-Ab

The same number of sequence- and structure-based models were developed for I-Ab as for H2-Db and H2-Kb: 3 docking-based QMs, 8 PLS and 14 ML models. Two PCs were extracted for the PLS model. Only the RBF model was validated as a robust predictive tool with a *sensitivity* from 75% to 100% and *specificity* of 67% (Table S7). This model was also used in the prediction of the pK_d_ of peptides binding to I-Ab.

### 2.6. Selection of Peptides for the Multi-Epitope Vaccine Prototype and Epitope Localization in the Structure of SARS-CoV-2 Proteins

Because of the differing binding site structures of mouse and human MHC alleles, peptides binding to one may not necessarily bind to the other. To identify overlapping peptides, we employed a set of known binders to human MHCs. We utilized derived models from this set to select peptides that bind to mouse MHC alleles.

**Figure 4:**
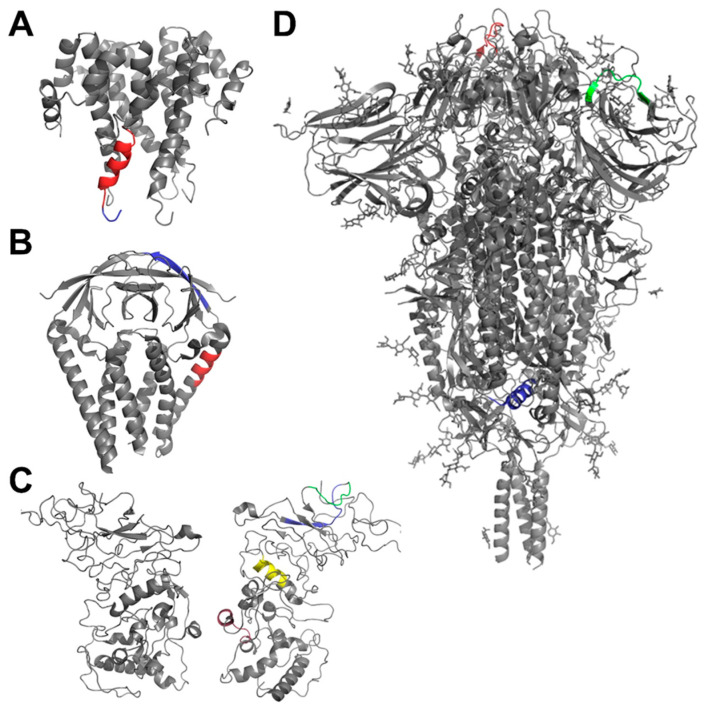
Localization of the epitopes from Table 2 on the structures of SARS-CoV-2 proteins. (**A**) E protein (PDB: 5 × 29): YSFVSEETG (PDB: 5 × 29 structure contains only the last three amino acids from this epitope) (blue) and TLIVNSVLLFLAF (red). (**B**) M protein (PDB: 8CTK): LSYYKLGAS (blue) and LSYFIASF (red). (**C**) N protein (PDB: 8FG2): AQFAPSASAF (red), WYFYYLGTGP (blue), AGLPYGAN (green), and LALLLLDRL (yellow). (**D**) S protein (PDB: 6XR8): QSYGFQPTNGV (red), IPFAMQMAYRFNGI (blue), and EFRVYSSANNCTFE (green). The proteins E, M, and N are dimers, and the opposite regions contain the same epitopes (not marked). The images were generated by PyMOL Molecular Graphics System, version 2.5.0.

Meyer et al. [42] have published a list of T cell epitopes from the Spike (S), Membrane (M), and Envelope (E) proteins of the SARS-CoV-2 virus, identified in convalescent patients with COVID-19. In the present study, we considered only epitopes recognized by at least three patients (Table 1). These epitopes were used as a starting set in the selection of prospective candidate epitopes among the mice MHC high binders. The T cell epitopes from the S, M, and E proteins of SARS-CoV-2 virus were presented as sets of overlapping nonamers/octamers and their p*K_d_* values were predicted by the best-performing models derived in this study. The fourth structural protein of SARS-CoV-2—the Nucleocapsid protein (N)—was also presented as sets of overlapping nonamers/octamers and the p*K_d_* values were predicted by the models. Asbinders were considered peptides predicted as such by two of the three models for H2-Db and H2-Kb. For the prediction of the binders to I-Ab, only the RBF model was used. We mapped the binders on the corresponding SARS-CoV-2 proteins and selected the sequences containing binders to the three mouse MHC alleles. Based on their hydrophobic potential to be included in lipid-based nanoparticles and the protocol restrictions for synthesis, eleven best-predicted MHC-I- and MHC-II-binding peptides from the structures of the S, M, N, and E SARS-CoV-2 proteins have been selected for the synthesis and animal experiments (Table 2). These peptides were synthesized with >98% purity (Caslo Laboratory, Lyngby, Denmark). The localization of the peptides is pictured on the X-ray structures of the E, M, N, and S proteins of the SARS-CoV-2 virus (Figure 4).

### 2.7. Immunization with SARS-CoV-2 Epitopes Remodels the Lymphocyte Profile

**Figure 5:**
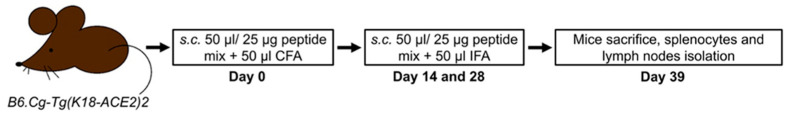
Scheme of treatment of transgenic mice expressing the human ACE2 receptor.

Groups of 10-week-old female B6.Cg-Tg(K18-ACE2)2Prlmn/J transgenic mice expressing the human angiotensin I-converting enzyme 2 (ACE2) receptor were randomly assigned to four test groups (five animals per cage), as described in the Materials and Methods. The treatment schedule is described in detail in the Materials and Methods and is illustrated in Figure 5. In brief, the animals from group 1 (control group) were injected subcutaneously (s.c.) with 50 µL PBS + 50 µL Complete Freund’s Adjuvant (CFA); the mice from group 2 were immunized with a single s.c. injection of 50 µL CFA + 50 µL mix of MHC-I peptides; group 3 received 50 µL CFA + mix of MHC-II peptides; and group 4 was injected with 50 µL CFA + mix of MHC-I + MHC-II peptides. After 14 days, the mice were boosted with Incomplete Freund’s Adjuvant (IFA) + 25 µg respective peptides or with PBS, and another boost was performed 14 days later. All mice were bled and were sacrificed on day 39. The collected sera were kept at −70 °C for further analyses.

**Figure 6:**
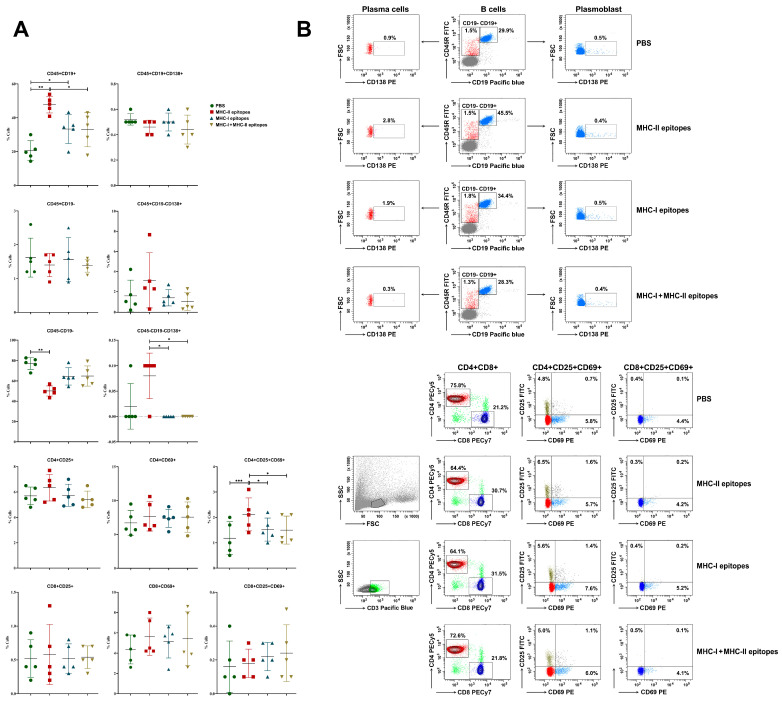
Flow cytometry analysis for splenocyte phenotyping. (**A**) Isolated lymphocytes from inguinal lymph nodes from all experimental mice were analyzed with combinations of anti-mouse antibodies, as described in Section 4. The extracted results from all experiments are presented graphically. Results are represented as mean ±SD (*n* = 5). Data were analyzed by the one-way ANOVA test (* *p* < 0.05; ** *p* < 0.01; *** *p* < 0.001). (**B**) Representative data of 5 experiments are shown.

A number of fluorescence-activated cell sorting (FACS) analyses have been performed in order to follow the changes in the B and T immune cell subsets after immunizations of B6.Cg-Tg(K18-ACE2)2Prlmn/J mice with the predicted MHC-I and MHC-II epitopes, as described in the Materials and Methods. A significant increase in the CD19+ B cell number was found in the group of mice injected with the MHC-II peptide mix compared to the control group, while a moderate statistically significant increase in the same cells was also detected in the mice immunized with the MHC-I peptide mix (Figure 6). This indicates the potential activation of the humoral arm of the immune system and an increase in the number of corresponding viral epitope-specific B cells. A statistically non-significant increase in the plasma cell number (CD19-CD138+) was found in the MHC-II peptide mix-immunized group. No changes were observed in the percentage of plasmablasts (CD19+CD138+) between the groups.

Focusing on T cells, a significant increase in double-activated (CD25+CD69+) CD4+ T cells was detected in the group of mice injected with the MHC-II peptide mix in comparison to the control animals and to the other two groups of peptide-treated mice. No significant differences were found in the percentage of activated CD8+ T cells between the groups, although the treatment with the peptide combinations exhibited the tendency to increase the percentage of activated CD8+ lymphocytes.

### 2.8. Detection of Mouse Anti-SARS-CoV-2 IgG Antibodies

**Figure 7:**
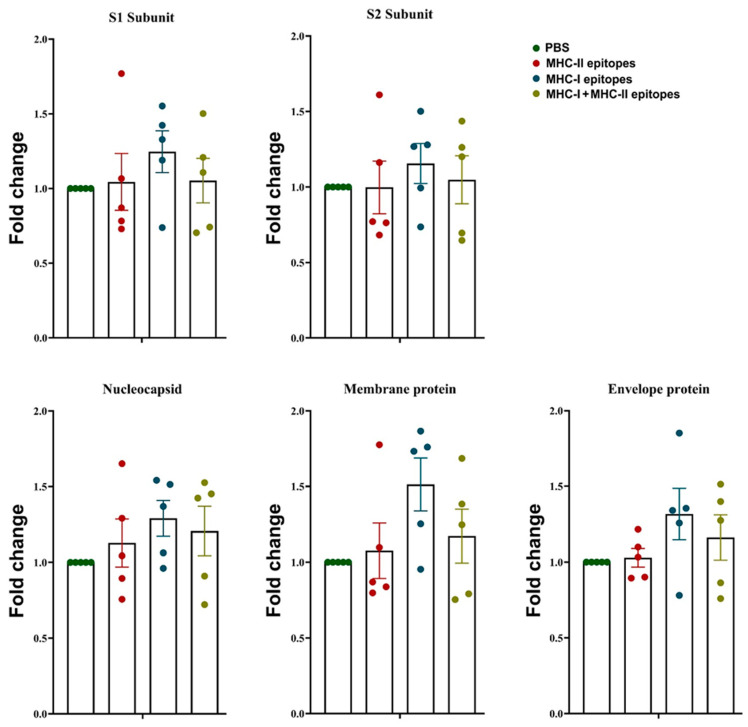
The humoral immune response against viral proteins was analyzed after immunization of B6.Cg-Tg(K18-ACE2)2Prlmn/J mice with SARS-CoV-2 peptide epitopes. Anti-S1, S2, M, N, and E protein IgG antibody levels were determined using ELISA. All samples were triplicated and average values were used for analysis. Summarized results for the calculated fold change obtained from individual mouse sera (*n* = 5) are presented and mean ± SD values were calculated for each group, in comparison to controls; *p* values were calculated using the one-way ANOVA test (*p* < 0.05) in comparison to each group. Representative data of four experiments are shown.

The in vivo effects of the predicted MHC-I and MHC-II epitopes were studied in the sera of B6.Cg-Tg(K18-ACE2)2Prlmn/J mice after immunization with the peptide combinations, and the levels of anti-SARS-CoV-2 IgG antibodies were determined by ELISA. As shown in Figure 7, the immunization of the experimental mice with the MHC-I, MHC-II, or MHC-I/MHC-II peptide epitope mixes in CFA, followed by double boosting with the same epitope combinations in IFA resulted in a non-significant increase in the fold change in the anti-SARS-CoV-2 IgG antibody levels after the last immunization. The administration of MHC-I peptide epitopes in CFA/IFA to the B6.Cg-Tg(K18-ACE2)2Prlmn/J mice led to the generation of the highest levels of anti-S1, anti-S2, anti-M, anti-N, and anti-E IgG antibodies compared to the CFA/IFA-treated control mice, while the immunization with the MHC-II peptide epitopes could not elicit an increased humoral levels. A weak non-significant increase was found after the immunization of the test animals with the MHC-I/MHC-II epitope mix.

### 2.9. Determination of IL-4- and IFN-γ-Producing T Cells

**Figure 8:**
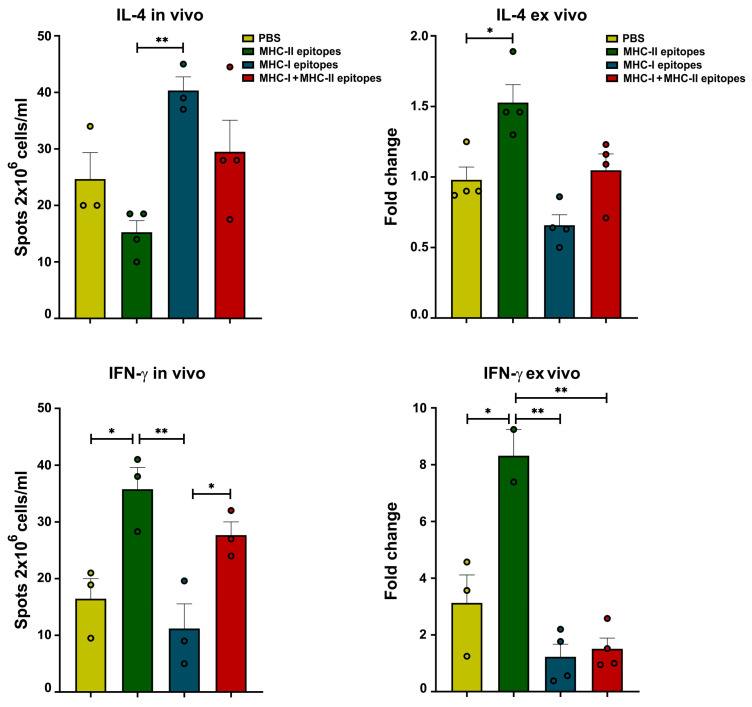
Cytokine production in the animals after in vivo SARS-CoV-2 peptide epitope administration. IL-4 and IFN-γ ELISpot assays were used to count the number of IL-4- and IFN-γ-producing T cells from mice treated in vivo with different mixes of peptides (**left panels**) or ex vivo with additional peptide stimulation (**right panels**). All samples were triplicated and the data are presented as mean ± SD for each group; p values were calculated using the one-way ANOVA test (* *p* < 0.05; ** *p* < 0.01) in comparison to each group. Representative data of four independent experiments are shown.

The in vivo and ex vivo effects of immunization with the predicted peptide epitopes on the production of IL-4 and IFN-γ from isolated mouse splenocytes were assessed by ELISpot. Significantly elevated levels of IL-4 were measured after the in vivo treatment of the animal group administered the MHC-I peptide epitope mix, while the same cytokine production was suppressed in the mice immunized with the MHC-II peptide epitopes (Figure 8). Opposite results were found after additional ex vivo stimulation of the respective mouse groups, and the IL-4 values were significantly higher in the MHC-II peptide-treated group compared to those in the control animals. Here, the production of IL-4 was suppressed in the mice immunized with the MHC-I peptide epitope mix. In both sets of experiments, a weak non-significant increase in IL-4 production was measured after the administration of the MHC-I/MHC-II peptide epitope mix.

A significant increase in the number of cells secreting IFN-γ was observed in the mouse group immunized in vivo with MHC-II peptide epitopes compared to the control animals and those treated with MHC-I peptides. The same results were found after the ex vivo incubation of the splenocytes from the respective groups with the peptide epitopes. While a significant increase in IFN-γ-producing cells was found in the mouse group treated in vivo with MHC-I/MHC-II peptides, lower numbers of splenocytes were counted in the same mice after ex vivo stimulation compared to in the controls.

## 3. Discussion

Multi-epitope vaccines are a new approach to vaccine development that have been gaining attention in recent years due to their potential to provide comprehensive protection against infectious diseases and cancer [33,43]. These vaccines combine multiple epitopes, or specific regions of a pathogen, to create a more effective immune response. They offer several benefits over traditional vaccines. By targeting multiple epitopes of a pathogen, these vaccines can provide protection against multiple strains of the targeted cause of infection. This is particularly important for pathogens that have many strains, such as SARS-CoV-2. In addition, these vaccines can be designed to be more specific, targeting only the most important epitopes of the pathogen, which reduces the risk of side effects.

A great number of designed vaccine constructs against SARS-CoV-2 based on immunoinformatic analysis have been published since the beginning of the pandemic, but only a few of them included experimental evidence about the immunogenicity of the proposed constructs [44,45,46]. Hundreds of B and T cell epitopes have been identified in the main surface proteins of the targeted virus, providing various opportunities for vaccine design and approaches for combinations to induce both specific humoral and cellular immunity. Although a T cell response is the desired effect of vaccination, efforts for the generation of virus-neutralizing antibodies are restricted by the occurrence of undesired effects such as antibody-dependent enhancement (ADE) of the viral infection.

Another important issue determining effective vaccine protection against SARS-CoV-2 is the high rate of mutations generated during virus adaptation and circulation among heterotrophic populations. Newly occurring mutations can increase the virulence of the virus; reduce the efficacy of vaccines; and avoid the protective immune response built up after infection. For this reason, it is essential that the conserved B and T cell epitopes in the core structural viral proteins are identified to avoid the high mutation rate among other epitopes subjected to mutagenic pressure. An appropriate vaccine design should include viral structural units and moieties that provide effective immunity and protection during a sustained epidemic with shifting viral strains. Bagherzadeh et al. analyzed the mutation rate in the immunodominant regions (IDRs), and in the predicted epitopes. Based on their conservativity and antigenicity score, the authors selected only 8 IDRs and 10 predicted epitopes out of all the epitopes with higher conservativity and immunogenicity [47].

Here, we present a multi-epitope vaccine prototype designed using the immunoinformatic approach and tailored to be immunogenic on a specific biological species, humanized-ACE2 transgenic B6.Cg-Tg(K18-ACE2)2Prlmn/J mice, which develop severe lung disease in response to SARS-CoV-2 infection. The efficacy of the designed vaccine epitopes was tested using in vitro, in vivo, and ex vivo tests. Furthermore, the epitopes included in our vaccine originate from the pool of human SARS-CoV-2 T cell epitopes identified by convalescent patients with COVID-19. This means that although the final vaccine encapsulated in lipid-based nanoparticles is designed to be immunogenic in mice, it can be expected to be immunogenic in humans as well.

The conserved structure of the selected epitopes from E (two epitopes), M (two epitopes), N (four epitopes), and S (three epitopes) proteins was unchanged not only in the frame of SARS-CoV-2, but also concerning SARS-CoV and MERS-CoV (Tables S8–S14). The unique design of selecting unchanged structures among pathogenic coronaviruses can provide vaccine content without the need for a redesign following viral mutations over time. These core epitopes could be a solid base for inclusion in nanoparticles for vaccine design.

Using the gold standard for adjuvanticity, coupled CFA/IFA can provide the expected information about the epitopes’ immunogenicity and their processing in immune cells, but this approach of immunization cannot provide a sufficient T cell response. The immunization with adjuvanted MHC-I, MHC-II, or MHC-I/MHC-II peptide epitopes of humanized-ACE2 transgenic mice leads to changes in the ratio and activation state of some immune cell populations. Even if not all the changes that occur are statistically proven, a significant increase in the CD19+ B cell number in the mice injected with the MHC-II peptide mix is an important indicator of B cell recognition and proliferation. Immunization with the same peptide epitope mix caused a significantly greater increase in double-activated (CD25+CD69+) CD4+ T cells compared to that in the control animals and the mice treated with MHC-I or MHC-I/MHC-II peptide epitopes. These B and T cell activations are an important basis for an effective immune response as a result of the involvement of both the humoral and cellular arms of immunity. All of these changes in the ratio and activation state of immune cells provide important data on the exact mechanisms of the immune response after immunization with a multi-epitope vaccine. Against the background of hundreds of developed in silico peptide vaccines against SARS-CoV-2 proposed for in vivo studies, real experimental data on immune system remodeling after vaccination are lacking [48]. Our results may support the immunological impact of vaccination on experimental animals, providing important information on the influence of the changing lymphocyte status in the immune response process.

Starting with 292 CD8+ and 284 CD4+ identified T cell epitopes, Smith et al. have selected 16 candidate vaccine peptides (10 T cell epitopes and 6 B cell epitopes). Following immunization in BALB/c mice, the authors observed a strong T cell response in 7/10 of the T cell epitope peptides, while no efficient humoral response was found due to the linear peptide epitopes used [49]. Pardieck at al. also found that high titers Spike-specific IgG antibodies against CpG/IFA adjuvanted linear B cell epitopes are not neutralizing against SARS-CoV-2 infection in K18-hACE2 transgenic mice, but a fully protective immune response was elicited after vaccination with a DNA vaccine encoding the entire Spike protein, proven by intranasal challenge with a lethal dose of SARS-CoV-2 [50]. In the same study, immunization with a single Spike protein-derived epitope resulted in a strong CD8+ T cell response, providing full protection against SARSCoV-2 after a double booster. The presence of high numbers of circulating and tissue-resident memory T cells is a promising strategy against SARS-CoV-2 even with missing neutralizing antibodies.

In our study, immunization with SARS-CoV-2 peptide epitopes in CFA that was boosted with the same peptides in IFA induced a weak humoral immune response against viral proteins (S1, S2, M, N, E) in B6.Cg-Tg(K18-ACE2)2Prlmn/J mice, but this is not surprising in many cases of such immunizations [49,50]. Instead, immunization with the selected epitopes resulted in significant changes in IL-4 cytokine production in vivo and ex vivo after immunization with MHC-I or MHC-II epitopes, respectively, which promoted B cell activation through Th2 cell differentiation. The T cell ELISpot is a highly sensitive assay used to determine the immune response to a specific antigen based on the cytokines released by T lymphocytes. The same assay demonstrated a significant increase in the number of IFN-γ-producing T cells in vivo after the vaccination of animals with MHC-II and MHC-I/MHC-II epitopes and ex vivo using MHC-II epitopes for immunization. As a result, we can expect the mobilization and activation of CD8+ T cells, which has the potential to protect against SARS-CoV-2. Heitman et al. also obtained SARS-CoV-2-specific T cell responses and IFNγ T cell production mediated by Th 1 CD4+ and CD8+ T cells by the injection of multiple T cell peptide epitopes derived from different viral proteins [51]. In another study, Song at al. proposed a synthetic SARS-CoV-2-derived T and B cell peptide epitope cocktail, which elicited full protection against SARS-CoV-2 infection in H11-K18-hACE2 mice [52]. 

Following our primary investigation, the selected peptides will be incorporated into specific lipid-based nanoparticles and will be administered to humanized-ACE2 transgenic B6.Cg-Tg(K18-ACE2)2Prlmn/J mice. We hypothesize that it may be possible to induce selectively elevated levels of anti-SARS-CoV-2 antibodies and a strong SARS-CoV-2-specific CTL response in a model of humanized-ACE2 transgenic C57B6 mice by administering constructed virus-like delivery particles. These engineered particles are expected to bind selectively to mannose receptors (MRs) and toll-like receptors (TLRs) on antigen-presenting cells (APCs), and to induce in these cells strong activating signals via their surface receptors. The mechanism is involved in antigen presentation and can potentially be translated to better immunizations and stronger B and CD8+ immune responses.

## 4. Materials and Methods

### 4.1. Monoclonal Antibodies

Anti-mouse Pacific Blue-conjugated CD3 (clone 145-2C11; #100334,) and CD19 (clone 6D5; #115523); Fluorescein isothiocyanate (FITC)-conjugated CD25 (clone PC61; #102006) and CD45R (B220) (clone RA3-6B2; #103206); Phycoerythrin (PE)-Cy7-conjugated CD8 (clone 53-5.8; #140416); PE-Cy5-conjugated CD4 (clone GK1.5; #100410); and PE-conjugated CD69 (clone H1.2F3; #104507) (BioLegend, Amsterdam, The Netherlands) and CD138 (clone 300506; #fab2966p) (R&D, Minneapolis, MN, USA) were used for fluorescence-activated cell sorting (FACS) experiments. Corresponding fluorescence-labeled isotype controls (BioLegend) were used to exclude nonspecific antibody binding.

HRP (horseradish peroxidase)-conjugated anti-mouse IgG (#405306, BioLegand) was used for enzyme-linked immunosorbent (ELISA) assay.

### 4.2. Mice

Groups of 10-week-old female B6.Cg-Tg(K18-ACE2)2Prlmn/J transgenic mice expressing the human angiotensin I-converting enzyme 2 (ACE2) receptor under the regulation of the cytokeratin 18 (K18) promoter, Strain #:034860 (obtained from The Jackson Laboratory, Bar Harbor, ME, USA), were randomly assigned to the test groups (five animals per cage). The mice were housed in a barrier-type animal house under specific-pathogen-free (SPF) conditions with ad libitum access to food and water. The room was kept on a reverse 12 hr light cycle. All animal experiments and the study protocols were approved by the Animal Care Commission at the Bulgarian Food Safety Agency (BFSA) (Licence #293) and were carried out in accordance with the International regulations (EU Directive 2010/63/EU) for the Care and Use of Laboratory Animals.

### 4.3. Peptide Synthesis and Treatment Schedule

All selected peptides from Table 2 (H2-Db and H2-Kb for MHC-I mix and I-Ab for MHC-II mix) were synthesized with >98% purity (Caslo Laboratory, Lyngby, Denmark) and used for immunization.

The experimental mice were divided into four groups (five animals per group) (Figure 5). The control animals (group 1) were injected subcutaneously (*s.c.*) with 50 µL PBS + 50 µL Complete Freund’s Adjuvant (CFA, Sigma-Aldrich, Taufkirchen, Germany); the mice from group 2 were immunized with a single *s.c.* injection of 50 µL CFA + 50 µL mix of MHC-I peptides (25 µg peptides per mouse, 1:1 CFA-to-peptide ratio); group 3 received 50 µL CFA + mix of MHC-II peptides (25 µg peptides per mouse); and group 4 was injected with 50 µL CFA + mix of MHC-I + MHC-II peptides (25 µg peptides per mouse). After 14 days, the mice were boosted with Incomplete Freund’s Adjuvant (IFA, Sigma-Aldrich) + 25 µg respective peptides per mouse (in 100 µL, 1:1 IFA-to-peptide ratio) or with PBS, and another boost was performed 14 days later (Figure 2). All mice were bled and were sacrificed on day 39. The collected sera were kept at −70 °C for further analyses.

### 4.4. Flow Cytometry Analysis

Inguinal lymph nodes were excised from the sacrificed B6.Cg-Tg(K18-ACE2)- 2Prlmn/J mice and passed through a 70 µm sterile cell strainer (BD Biosciences, Erenbodegem, Belgium) to prepare monocellular suspensions. After lysis of erythrocytes with hypotonic ammonium chloride solution, the lymphocytes were washed with FACS Buffer (PBS, 2.5% fetal calf serum (FCS) and 0.05% sodium azide). The isolated cells were counted and transferred into FACS tubes (BD Falcon, BD Biosciences, San Diego, CA, USA), washed twice, and incubated (2 × 10^5^ cells/tube) for 20 min on ice with one of the following mixes of anti-mouse antibodies: CD3-Pacific Blue, CD4-PE-Cy5, CD8-PE-Cy7, CD25-FITC, and CD69-PE antibodies for CD4 and CD8 T cell populations; CD19-Pacific Blue, CD45R-FITC, and CD138 PE antibodies for B and plasma cells.

After the incubation, the cells were washed twice, and 30,000 lymphocyte-gated cells from each sample were analyzed with a BD LSR II flow cytometer using the Diva v6.1.3. software (BD Biosciences, San Jose, CA, USA).

### 4.5. ELISA for Anti-SARS-CoV-2 IgG Antibodies

SARS-CoV-2 S1 subunit (#230-01101, RayBiotech Life, Peachtree Corners, GA, USA), S2 (#230-01103, RayBiotech Life), Nucleocapsid protein (#230-30164, RayBiotech Life), Membrane glycoprotein (#230-01124, RayBiotech Life), and Envelope protein (# ENN-C5128, ACROBiosystems, Newark, DE, USA) were diluted at concentration 0.5 µg/mL in carbonate buffer (NaHCO_3_, pH-9.6) and were used to coat 96-well immunoplates (Maxisorp, Nunc, Roskilde, Denmark) overnight at 4 °C. Next, the plates were washed with PBS/0.05% Tween 20 (T-PBS) and blocked with 1% gelatin (#G-7765, Sigma, Germany) for one hour at room temperature. Further, the plates were incubated with diluted mouse sera, followed by incubation with HRP-conjugated anti-mouse IgG antibody. The reaction was developed with TMB (3,3′, 5,5″-tetramethylbenzidine) substrate, stopped with 2N H_2_SO_4_ and measured at 450 nm (CLARIOstar Plus, BMG Labtech, Ortenberg, Germany).

### 4.6. ELISpot Assay

Mouse IL-4 ELISpot (CT319-PR5) and Mouse IFN-γ ELISpot (CT317-PR5) kits (U-CyTech biosciences, Utrecht, The Netherlands) were used to count the number of IL-4- and IFN-γ-producing T cells from mice treated in vivo with different mixes of peptides. The spleens were surgically removed from all experimental B6.Cg-Tg(K18-ACE2)2Prlmn/J mice, and monocellular suspensions were prepared as described above. Isolated splenocytes were cultured (2 × 10^5^ cells/well) in complete RPMI (Roswell Park Memorial Institute Medium) 1640 medium (HiMedia Laboratories GmbH, Einhausen, Germany) without further stimulation for 24 h at 37 °C/5% CO_2._

In a separate experiment, the same splenocytes were co-cultured in the presence of 15 µg/mL MHC-I, MHC-II, or MHC-I/MHC-II peptide mixes corresponding to the immunization protocol to assess the ex vivo effect. The splenocytes from the control mice were cultured in RPMI medium only. Next, the ELISpot assay was performed according to the manufacturer’s instructions. The number of IL-4- and IFN-γ-producing T cells was assessed by counting the corresponding colored spots in the wells using C.T.L. Immunospot S6 Ultimate UV Image Analyzer (Bonn, Germany).

### 4.7. Statistical Analysis

All statistical analyses were performed with GraphPad Prism 5 software (San Diego, CA, USA). Data normality was assessed with a Shapiro–Wilk test and Kolmogorov–Smirnov normality test. The one-way ANOVA test followed by Tukey’s multiple comparisons test were used to determine the differences between each two groups. Values in the figures correspond to mean ± SD. A value of *p* < 0.05 was considered statistically significant.

## 5. Conclusions

The aim of the present study is the knowledge-based development of a next-generation novel multi-epitope vaccine for the preventive therapy of SARS-CoV-2. Immunization with mixes of selected SARS-CoV-2 epitopes remodeled the lymphocyte profile in B6.Cg-Tg(K18-ACE2)2Prlmn/J mice. A weak humoral response and significant production of IL-4 and IFN-γ from T cells were found after the vaccination of the animals. The multi-epitope vaccine prototype presented in this study demonstrates immunogenicity in the mice and shows potential for human vaccine construction. In the next step, the selected peptides will be loaded onto specific lipid-based delivery nanoparticles expected to bind selectively to mannose receptors and toll-like receptors on the antigen-presenting cells, and to induce in these cells strong activating signals via their surface receptors after immunization of the same mouse strain.

## Figures and Tables

**Table 1:** Human SARS-CoV-2 T cell epitopes identified in at least three convalescent patients with COVID-19 [29].

Peptide ID in [24]	Protein	Peptide Position	Peptide
**1**	E	1–15	MYSFVSEETGTLIVN
**2**	M	13–27	LKKLLEQWNLVIGFL
**4**	M	93–107	LSYFIASFRLFARTR
**5**	M	97–113	IASFRLFARTRSMWSFN
**6**	M	146–160	RGHLRIAGHHLGRCD
**7**	M	165–179	PKEITVATSRTLSYY
**8**	M	175–190	TLSYYKLGASQRVAGD
**9**	M	201–217	IGNYKLNTDHSSSSDNI
**11**	S	87–102	NDGVYFASTEKSNIIR
**13**	S	154–171	ESEFRVYSSANNCTFEYV
**19**	S	347–364	FASVYAWNRKRISNCVAD
**20**	S	446–468	GGNYNYLYRLFRKSNLKPFERDI
**22**	S	492–508	LQSYGFQPTNGVGYQPY
**26**	S	895–911	QIPFAMQMAYRFNGIGV

**Table 2:** SARS-CoV-2 peptides predicted to bind to H2-Db, H2-Kb, and I-Ab and selected for synthesis and animal experiments.

Peptide ID in [29]	Protein	Peptide Position	Peptide	Binding to
**1**	E	2–10	YSFVSEETG	I-Ab
**1**	E	11–23	TLIVNSVLLFLAF	H2-Db, H2-Kb
**4**	M	93–100	LSYFIASF	H2-Kb
**8**	M	176–184	LSYYKLGAS	I-Ab
**13**	S	156–169	EFRVYSSANNCTFE	H2-Db, I-Ab
**22**	S	493–503	QSYGFQPTNGV	H2-Kb, I-Ab
**26**	S	896–909	IPFAMQMAYRFNGI	H2-Db, H2-Kb, I-Ab
**-**	N	107–116	WYFYYLGTGP	H2-Kb, I-Ab
**-**	N	118–125	AGLPYGAN	H2-Kb
**-**	N	218–226	LALLLLDRL	H2-Db
**-**	N	304–313	AQFAPSASAF	I-Ab

## Data Availability

Data is contained within the article and Supplementary Material.

## Supplementary Materials

The following supporting information can be downloaded at https://www.mdpi.com/article/10.3390/ph17111498/s1: Table S1. Input settings of the molecular docking protocols used in the present study. Table S2. Quantity of peptides within the datasets used in the present study. The training sets consist of peptides with quantitatively measured affinities. Test sets A–D contain binders measured qualitatively by radioactivity or fluorescence or mass spectrometry. Test set N includes non-binders. Table S3. Sensitivity and specificity of the best-performing models for peptide binding to H2-Db. Table S4. PLS-based quantitative matrix (QM) for peptide binding to H2-Db. The free term in the model is 5.649. Table S5. Sensitivity and specificity of the best-performing models for peptide binding to H2-Kb. Table S6. PLS-based quantitative matrix (QM) for peptide binding to H2-Kb. The free term in the model is 6.024. Table S7. Sensitivity and specificity of the best-performing model for peptide binding to I-Ab. Table S8. Top 100 protein sequence alignments of E proteins from SARS-CoV-2 and MERS-CoV. Table S9. Top 100 protein sequence alignments of M proteins from SARS-CoV-2 and MERS-CoV. Table S10. Top 100 protein sequence alignments of N proteins from SARS-CoV-2 and MERS-CoV from position 100 to position 150. Table S11. Top 100 protein sequence alignments of N proteins from SARS-CoV-2 and MERS-CoV form position 200 to position 320. Table S12. Top 100 protein sequence alignments of S proteins from SARS-CoV-2 and MERS-CoV from position 151 to position 234. Table S13. Top 100 protein sequence alignments of S proteins from SARS-CoV-2 and MERS-CoV from position 490 to position 550. Table S14. Top 100 protein sequence alignments of S proteins from SARS-CoV-2 and MERS-CoV from position 890 to position 920.
