# Supplementary material for: Design, Development and Immunogenicity Study of a Multi-Epitope Vaccine Prototype Against SARS-CoV-2
Source: Pharmaceuticals (Basel). 2024 Nov 7;17(11):1498. doi: 10.3390/ph17111498 (PMC11597159; doi:10.3390/ph17111498)
Supplement: Supplementary file 1 [file Table_S10_N_protein_alignment[100..150].pdf › Table_S10_N_protein_alignment[100..150].pdf]

NCBI Multiple Sequence Alignment Viewer, Version 1.25.2

Sequence ID	Start	Alignment	End	Organism
		100 110 120 130 140 150		
QOC66901.1 (+)	1	KMKDLSPRWYFYYLGTGPEAGLPYGANKDGIIWVATEGALNTPKDHIGTRN	419	Severe acute respiratory...
YP_009724397.2 (+)	1	KMKDLSPRWYFYYLGTGPEAGLPYGANKDGIIWVATEGALNTPKDHIGTRN	419	Severe acute respiratory...
WPW43103.1 (+)	1	KMKDLSPRWYFYYLGTGPEAGLPYGANKDGIJWVATEGALNTPKDHIGTRN	419	Severe acute respiratory...
QSM21170.1 (+)	1	KMKDLSPRWYFYYLGTGPEAGLPYGANKDGIIWVATEGALNTPKDHIGTRN	419	Severe acute respiratory...
QQV43073.1 (+)	1	KMKDLSPRWYFYYLGTGPEAGLPYGANKDGIIWVATEGALNTPKDHIGTRN	419	Severe acute respiratory...
WKQ39290.1 (+)	1	KMKDLSPRWYFYYLGTGPEAGLPYGANKDGIIWVATEGALNTPKDHIGTRN	419	Severe acute respiratory...
QLC94852.1 (+)	1	KMKDLSPRWYFYYLGTGPEAGLPYGANKDGIIWVATEGALNTPKDHIGTRN	419	Severe acute respiratory...
UPD16481.1 (+)	1	KMKDLSPRWYFYYLGTGPEAGLPYGANKDGIIWVATEGALNTPKDHIGTRN	419	Severe acute respiratory...
WWQ82479.1 (+)	1	KMKDLSPRWYFYYLGTGPEAGLPYGANKDGIIWVATEGALNTPKDHIGTRN	419	Severe acute respiratory...
QNO40616.1 (+)	1	KMKDLSPRWYFYYLGTGPEAGLPYGANKDGIIWVATEGALNTPKDHIGTRN	419	Severe acute respiratory...
QOL73089.1 (+)	1	KMKDLSPRWYFYYLGTGPEAGLPYGANKDGIIWVATEGALNTPKDHIGTRN	419	Severe acute respiratory...
UBE86422.1 (+)	1	KMKDLSPRWYFYYLGTGPEAGLPYGANKDGIIWVATEGALNTPKDHIGTRN	419	Severe acute respiratory...
UXB96785.1 (+)	1	KMKDLSPRWYFYYLGTGPEAGLPYGANKDGIIWVATEGALNTPKDHIGTRN	419	Severe acute respiratory...
QOI09455.1 (+)	1	KMKDLSPRWYFYYLGTGPEAGLPYGANKDGIIWVATEGALNTPKDHIGTRN	419	Severe acute respiratory...
QKE46048.1 (+)	1	KMKDLSPRWYFYYLGTGPESGLPYGANKDGIIWVATEGALNTPKDHIGTRN	419	Severe acute respiratory...
QSY42010.1 (+)	1	KMKDLSPRWYFYYLGTGPEAGLPYGANKDGIIWVATEGALNTPKDHIGTRN	419	Severe acute respiratory...
UYG85820.1 (+)	1	KMKDLSPRWYFYYLGTGPEAGLPYGANKDGIIWVATEGALNTPKDHIGTRN	419	Severe acute respiratory...
WJZ58491.1 (+)	1	KMKDLSPRWYFYYLGTGPEAGLPYGANKDGIIWVATEGALNTPKDHIGTRN	419	Severe acute respiratory...
ULF05100.1 (+)	1	KMKDLSPRWYFYYLGTGPEAGLPYGANKDGIIWVATEGALNTPKDHIGTRN	419	Severe acute respiratory...
UHE10300.1 (+)	1	KMKDLSPRWYFYYLGTGPEAGLPYGANKDGIIWVATEGALNTPKDHIGTRN	419	Severe acute respiratory...
ULD97398.1 (+)	1	KMKDLSPRWYFYYLGTGPEAGLPYGANKDGIIWVATEGALNTPKDHIGTRN	419	Severe acute respiratory...
QNA39423.1 (+)	1	KMKDLSPRWYFYYLGTGPEAGLPYGANKDGIIWVATEGALNTPKDHIGTRN	419	Severe acute respiratory...
WKJ48376.1 (+)	1	KMKDLSPRWYFYYLGTGPEAGLPYGANKDGIIWVATEGALNTPKDHIGTRN	419	Severe acute respiratory...
QLL36249.1 (+)	1	KMKDLSPRWYFYYLGTGPEAGLPYGANKDGIIWVATEGALNTPKDHIGTRN	419	Severe acute respiratory...
QKS42641.1 (+)	1	KMKDLSPRWYFYYLGTGPEAGLPYGANKDGIIWVATEGALNTPKDHIGTRN	419	Severe acute respiratory...
QNH88939.1 (+)	1	KMKDLSPRWYFYYLGTGPEAGLPYGANKDGIIWVATEGALNTPKDHIGTRN	419	Severe acute respiratory...
QPN00879.1 (+)	1	KMKDLSPRWYFYYLGTGPEAGLPYGANKDGIIWVATEGALNTPKDHIGTRN	419	Severe acute respiratory...
QMJ00989.1 (+)	1	KMKDLSPRWYFYYLGTGPEAGLPYGANKDGIIWVATEGALNTPKDHIGTRN	419	Severe acute respiratory...
QIQ49839.1 (+)	1	KMKDLSPRWYFYYLGTGPEAGLPYGANKDGIIWVATEGALNTPKDHIGTRN	419	Severe acute respiratory...
QOU89326.1 (+)	1	KMKDLSPRWYFYYLGTGPEAGLPYGANKDGIIWVATEGALNTPKDHIGTRN	419	Severe acute respiratory...
QIZ64406.1 (+)	1	KMKDLSPRWYFYYLGTGPEAGLPYGANKDGIIWVATEGALNTPKDHIGTRN	419	Severe acute respiratory...
QLD94726.1 (+)	1	KMKDLSPRWYFYYLGTGPEAGLPYGANKDGIIWVATEGALNTPKDHIGTRN	419	Severe acute respiratory...
QJD20845.1 (+)	1	KMKDLSPRWYFYYLGTGPEAGLPYGANKDGIIWVATEGALNTPKDHIGTRN	419	Severe acute respiratory...
QKG88570.1 (+)	1	KMKDLSPRWYFYYLGTGPEAGLPYGANKDGIIWVATEGALNTPKDHIGTRN	419	Severe acute respiratory...
QKN20771.1 (+)	1	KMKDLSPRWYFYYLGTGPEAGLPYGANKDGIIWVATEGALNTPKDHIGTRN	419	Severe acute respiratory...
WEW62783.1 (+)	1	KMKDLSPRWYFYYLGTGPEAGLPYGANKDGIIWVATEGALNTPKDHIGTRN	419	Severe acute respiratory...
UOK30060.1 (+)	1	KMKDLSPRWYFYYIGTGPEAGLPYGANKDGIIWVATEGALNTPKDHIGTRN	419	Severe acute respiratory...
QOF20005.1 (+)	1	KMKDLSPRWYFYYLGTGPEAGLPYGANKDGIIWVATEGALNTPKDHIGTRN	419	Severe acute respiratory...
QOW08093.1 (+)	1	KMKDLSPRWYFYYLGTGPEAGLPYGANKDGIIWVATEGALNTPKDHIGTRN	419	Severe acute respiratory...
WPD73506.1 (+)	1	KMKDLSPRWYFYYLGTGPEAGLPYGANKDGIIWVATEGAVNTPKDHIGTRN	419	Severe acute respiratory...
QLB39364.1 (+)	1	KMKDLSPRWYFYYLGTGPEAGLPYGANKDGIIWVATEGALNTPKDHIGTRN	419	Severe acute respiratory...
UOA74334.1 (+)	1	KMKDLSPRWYFYYLGTGPEAGLPYGANKDGIIWVATEGALNTPKDHIGTRN	419	Severe acute respiratory...
QJD24157.1 (+)	1	KMKDLSPRWYFYYLGTGPEAGLPYGANKDGIIWVATEGALNTPKDHIGTRN	419	Severe acute respiratory...
QKE54130.1 (+)	1	KMKDLSPRWYFYYLGTGPEAGLPYGANKDGIIWVATEGALNTPKDHIGTRN	419	Severe acute respiratory...
QOL72261.1 (+)	1	KMKDLSPRWYFYYLGTGPEAGLPYGANKDGIIWVATEGALNTPKDHIGTRN	419	Severe acute respiratory...
WPR38736.1 (+)	1	KMKDLSPRWYFYYLGTGPEAGLPYGANKDGIIWVATEGALNTPKDHIGTRN	419	Severe acute respiratory...
QJD48383.1 (+)	1	KMKDLSPRWYFYYLGTGPEAGLPYGANKDGIIWVATEGALNTPKDHIGTRN	419	Severe acute respiratory...
QOF07825.1 (+)	1	KMKDLSPRWYFYYLGTGPEAGLPYGANKDGIIWVATEGALNTPKDHIGTRN	419	Severe acute respiratory...
QOY59133.1 (+)	1	KMKDLSPRWYFYYLGTGPEAGLPYGANKDGIIWVATEGALNTPKDHIGTRN	419	Severe acute respiratory...
QRG23177.1 (+)	1	KMKDLSPRWYFYYLGTGPEAGLPYGANKDGIIWVATEGALNTPKDHIGTRN	419	Severe acute respiratory...
QSO40789.1 (+)	1	KMKDLSPRWYFYYLGTGPEAGLPYGANKDGIIWVATEGALNTPKDHIGTRN	419	Severe acute respiratory...
QJR95597.1 (+)	1	KMKDLSPRWYFYYLGTGPEAGLPYGANKDGIIWVATEGALNTPKDHIGTRN	419	Severe acute respiratory...
QKF96015.1 (+)	1	KMKDLSPRWYFYYLGTGPEAGLPYGANKDGIIWVATEGALNTPKDHIGTRN	419	Severe acute respiratory...
QNI23493.1 (+)	1	KMKDLSPRWYFYYLGTGPEAGLPYGANKDGIIWVATEGALNTPKDHIGTRN	419	Severe acute respiratory...
QKG87202.1 (+)	1	KMKDLSPRWYFYYLGTGPEAGLPYGANKDGIIWVATEGALNTPKDHIGTRN	419	Severe acute respiratory...
WGC60343.1 (+)	1	KMKDLSPRWYFYYLGTGPEAGLPYGANKDGIIWVATEGALNTPKDHIGTRN	419	Severe acute respiratory...
WKO31098.1 (+)	1	KMKDLSPRWYFYYLGTGPEAGLPYGANKDGIIWVATEGALNTPKDHIGTRN	419	Severe acute respiratory...
QOI61828.1 (+)	1	KMKDLSPRWYFYYLGTGPEAGLPYGANKDGIIWVATEGALNTPKDHIGTRN	419	Severe acute respiratory...
QJD47102.1 (+)	1	KMKDLSPRWYFYYLGTGPEAGLPYGANKDGIIWVATEGALNTPKDHIGTRN	419	Severe acute respiratory...
UBE99971.1 (+)	1	KMKDLSPRWYFYYLGTGPEAGLPYGANRDGIIWVATEGALNTPKDHIGTRN	419	Severe acute respiratory...
QJD24961.1 (+)	1	KMKDLSPRWYFYYLGTGPEAGLPYGANKDGIIWVATEGALNTPKDHIGTRN	419	Severe acute respiratory...
QKS67451.1 (+)	1	KMKDLSPRWYFYYLGTGPEAGLPYGANKDGIIWVATEGALNTPKDHIGTRN	419	Severe acute respiratory...
QYA66272.1 (+)	1	KMKDLSPRWYFYYLGTGPEAGLPYGANKDGIIWVATEGALNTPKDHIGTRN	419	Severe acute respiratory...
UCK97592.1 (+)	1	KMKDLSPRWYFYYLGTGPEAGLPYGANKDGIIWVATEGALNTPKDHIGTRN	419	Severe acute respiratory...
QKV06267.1 (+)	1	KMKDLSPRWYFYYLGTGPEAGLPYGANKDGIIWVATEGALNTPKDHIGTRN	419	Severe acute respiratory...
QNN95553.1 (+)	1	KMKDLSPRWYFYYLGTGPEAGLPYGANKDGIIWVATEGALNTPKDHIGTRN	419	Severe acute respiratory...
XHN05705.1 (+)	1	KMKDLSPRWYFYYLGTGPEAGLPYGANKDGIIWVATEGALNTPKDHIGTRN	419	Severe acute respiratory...
UOH29770.1 (+)	1	KMKDLSPRWYFYYLGTGPEAGLPYGANKDGIIWVATEGALNTPKDHIGTRN	418	Severe acute respiratory...
QKU32077.1 (+)	1	KMKDLSPRWYFYYLGTGPEAGLPYGANKDGIIWVATEGALNTPKDHIGTRN	419	Severe acute respiratory...
QQY88248.1 (+)	1	KMKDLSPRWYFYYLGTGPEAGLPYGANKDGIIWVATEGALNTPKDHIGTRN	419	Severe acute respiratory...
WKO23443.1 (+)	1	KMKDLSPRWYFYYLGTGPEAGLPYGANKDGIIWVATEGALNTPKDHIGTRN	419	Severe acute respiratory...
QKS65664.1 (+)	1	KMKDLSPRWYFYYLGTGPEAGLPYGANKDGIIWVATEGALNTPKDHIGTRN	419	Severe acute respiratory...
QOQ72276.1 (+)	1	KMKDLSPRWYFYYLGTGPEAGLPYGANKDGIIWVATEGALNTPKDHIGTRN	419	Severe acute respiratory...
QXX41536.1 (+)	1	KMKDLSPRWYFYYLGTGPEAGLPYGANKDGIIWVATEGALNTPKDHIGTRN	419	Severe acute respiratory...
QMT91248.1 (+)	1	KMKDLSPRWYFYYLGTGPEAGLPYGANKDGIIWVATEGALNTPKDHIGTRN	419	Severe acute respiratory...
UDA98239.1 (+)	1	KMKDLSPRWYFYYLGTGPEAGLPYGANKDGIIWVATEGALNTPKDHIGTRN	419	Severe acute respiratory...
QJX69490.1 (+)	1	KMKDLSPRWYFYYLGTGPEAGLPYGANKDGIIWVATEGALNTPKDHIGTRN	419	Severe acute respiratory...
UCL02383.1 (+)	1	KMKDLSPRWYFYYLGTGPEAGLPYGANKDGIIWVATEGALNTPKDHIGTRN	419	Severe acute respiratory...
QYX84784.1 (+)	1	KMKDLSPRWYFYYLGTGPEAGLPYGANKDGIIWVATEGALNTPKDHIGTRN	419	Severe acute respiratory...
QRG18737.1 (+)	1	KMKDLSPRWYFYYLGTGPEAGLPYGANKDGIIWVATEGALNTPKDHIGTRN	419	Severe acute respiratory...
QIS60878.1 (+)	1	KMKDLSPRWYFYYLGTGPEAGLPYGANKDGIIWVATEGALNTPKDHIGTRN	419	Severe acute respiratory...
QPV20115.1 (+)	1	KMKDLSPRWYFYYLGTGPEAGLPYGANKDGIIWVATEGALNTPKDHIGTRN	419	Severe acute respiratory...
URG23179.1 (+)	1	KMKDLSPRWYFYYLGTGPEAGLPYGANKDGIIWVATEGALNTPKDHIGTRN	419	Severe acute respiratory...
QZC53203.1 (+)	1	KMKDLSPRWYFYYLGTGPEAGLPYGANKDGIIWVATEGALNTPKDHIGTRN	419	Severe acute respiratory...
QOS50197.1 (+)	1	KMKDLSPRWYFYYLGTGPEAGLPYGANKDGIIWVATEGALNTPKDHIGTRN	419	Severe acute respiratory...
QZP75395.1 (+)	1	KMKDLSPRWYFYYLGTGPEAGLPYGANKDGIIWVATEGALNTPKDHIGTRN	419	Severe acute respiratory...
QMX86289.1 (+)	1	KMKDLSPRWYFYYLGTGPEAGLPYGANKDGIIWVATEGALNTPKDHIGTRN	419	Severe acute respiratory...
QNU12530.1 (+)	1	KMKDLSPRWYFYYLGTGPEAGLPYGANKDGILWVATEGALNTPKDHIGTRN	419	Severe acute respiratory...
WKQ08845.1 (+)	1	KMKDLSPRWYFYYLGTGPEAGLPYGANKDGIIWVATEGALNTPKDHIGTRN	419	Severe acute respiratory...
QZJ78431.1 (+)	1	KMKDLSPRWYFYYLGTGPEAGLPYGANKDGIIWVATEGALNTPKDHIGTRN	419	Severe acute respiratory...
QJR88385.1 (+)	1	KMKDLSPRWYFYYLGTGPEAGLPYGANKDGIIWVATEGAFNTPKDHIGTRN	419	Severe acute respiratory...
QJU11477.1 (+)	1	KMKDLSPRWYFYYLGTGPEAGLPYGANKDGIIWVATEGALNTPKDHIGTRN	419	Severe acute respiratory...
QKV26073.1 (+)	1	KMKDLSPRWYFYYLGTGPEAGLPYGANKDGIIWVVTEGALNTPKDHIGTRN	419	Severe acute respiratory...
WKT26399.1 (+)	1	KMKDLSPRWYFYYLGTGPEAGLPYGVNKDGIIWVATEGALNTPKDHIGTRN	419	Severe acute respiratory...
QMT51417.1 (+)	1	KMKDLSPRWYFYYLGTGPEAGLPYGANKDGIIWVATEGALNTPKDHIGTRN	419	Severe acute respiratory...
UFQ73927.1 (+)	1	KMKDLSPRWYFYYLGTGPEAGLPYGANKDGIIWVATEGALNTPKDHIGTRN	419	Severe acute respiratory...
QNN88258.1 (+)	1	KMKDLSPRWYFYYLGTGPEAGLPYGANKDGIIWVATEGALNTPKDHIGTRN	419	Severe acute respiratory...
QMJ20502.1 (+)	1	KMKDLSPRWYFYYLGTGPEAGLPYGANKDGIIWVATEGALNTPKDHIGTRN	419	Severe acute respiratory...
QSX72020.1 (+)	1	KMKDLSPRWYFYYLGTGPEAGLPYGANKDGIIWVATEGALNTPKDHIGTRN	419	Severe acute respiratory...
QLL35362.1 (+)	1	KMKDLSPRWYFYYLGTGPEAGLPYGANKDGIIWVATEGALNTPKDHIGTRN	419	Severe acute respiratory...
