# Supplementary material for: Design, Development and Immunogenicity Study of a Multi-Epitope Vaccine Prototype Against SARS-CoV-2
Source: Pharmaceuticals (Basel). 2024 Nov 7;17(11):1498. doi: 10.3390/ph17111498 (PMC11597159; doi:10.3390/ph17111498)
Supplement: Supplementary file 1 [file Table_S12_S_protein_alignment[151..234].pdf › Table_S12_S_protein_alignment[151..234].pdf]

NCBI Multiple Sequence Alignment Viewer, Version 1.25.2

Sequence ID		Start	Alignment	End	Organism
			151 160 170 180 190 200 210 220 230 234		
BCN86353.1	(+)	1	GVYYHKNNKSWMESEFRVYSSANNCTFEYVSQPFLMDLEGKQGNFKNLREFVFKNIDGYFKIYSKHTPINLVRDLPQGFSALEP	1,282	Severe acute respiratory...
6XR8_A	(+)	1	GVYYHKNNKSWMESEFRVYSSANNCTFEYVSQPFLMDLEGKQGNFKNLREFVFKNIDGYFKIYSKHTPINLVRDLPQGFSALEP	1,310	Severe acute respiratory...
QJF75467.1	(+)	1	GVYYHKNNKSWMESEFRVYSSANNCTFEYVSQPFLMDLEGKQGNFKNLREFVFKNIDGYFKIYSKHTPINLVRDLPQGFSALEP	1,273	Severe acute respiratory...
8DT3_C	(+)	1	GVYYHKNNKSWMESEFRVYSSANNCTFEYVSQPFLMDLEGKQGNFKNLREFVFKNIDGYFKIYSKHTPINLVRDLPQGFSALEP	1,281	Severe acute respiratory...
QJX45031.1	(+)	1	GVYYHKNNKSWMESEFRVYSSANNCTFEYVSQPFLMDLEGKQGNFKNLREFVFKNIDGYFKIYSKHTPINLVRDLPQGFSALEP	1,273	Severe acute respiratory...
QJR85953.1	(+)	1	GVYYHKNNKSWMESEFRVYSSANNCTFEYVSQPFLMDLEGKQGNFKNLREFVFKNIDGYFKIYSKHTPINLVRDLPQGFSALEP	1,273	Severe acute respiratory...
YP_009724390.1	(+)	1	GVYYHKNNKSWMESEFRVYSSANNCTFEYVSQPFLMDLEGKQGNFKNLREFVFKNIDGYFKIYSKHTPINLVRDLPQGFSALEP	1,273	Severe acute respiratory...
QRN64146.1	(+)	1	GVYYHKNNKSWMESEFRVYSSANNCTFEYVSQPFLMDLEGKQGNFKNLREFVFKNIDGYFKIYSKHTPINLVRDLPQGFSALEP	1,273	Severe acute respiratory...
QNN86157.1	(+)	1	GVYYHKNNKSWMESEFRVYSSANNCTFEYVSQPFLMDLEGKQGNFKNLREFVFKNIDGYFKIYSKHTPINLVRDLPQGFSALEP	1,273	Severe acute respiratory...
QJF77846.1	(+)	1	GVYYHKNNKSWMESEFRVYSSANNCTFEYVSQPFLMDLEGKQGNFKNLREFVFKNIDGYFKIYSKHTPINLVRDLPQGFSALEP	1,273	Severe acute respiratory...
QRN78371.1	(+)	1	GVYYHKNNKSWMESEFRVYSSANNCTFEYVSQPFLMDLEGKQGNFKNLREFVFKNIDGYFKIYSKHTPINLVRDLPQGFSALEP	1,273	Severe acute respiratory...
QMS52716.1	(+)	1	GVYYHKNNKSWMESEFRVYSSANNCTFEYVSQPFLMDLEGKQGNFKNLREFVFKNIDGYFKIYSKHTPINLVRDLPQGFSALEP	1,273	Severe acute respiratory...
QIZ16509.1	(+)	1	GVYYHKNNKSWMESEFRVYSSANNCTFEYVSQPFLMDLEGKQGNFKNLREFVFKNIDGYFKIYSKHTPINLVRDLPQGFSALEP	1,273	Severe acute respiratory...
QMI90807.1	(+)	1	GVYYHKNNKSWMESEFRVYSSANNCTFEYVSQPFLMDLEGKQGNFKNLREFVFKNIDGYFKIYSKHTPINLVRDLPQGFSALEP	1,273	Severe acute respiratory...
QIZ97039.1	(+)	1	GVYYHKSNKSWMESEFRVYSSANNCTFEYVSQPFLMDLEGKQGNFKNLREFVFKNIDGYFKIYSKHTPINLVRDLPQGFSALEP	1,273	Severe acute respiratory...
QKS90791.1	(+)	1	GVYYHKNNKSWMESEFRVYSSANNCTFEYVSQPFLMDLEGKQGNFKNLREFVFKNIDGYFKIYSKHTPINLVRDLPQGFSALEP	1,273	Severe acute respiratory...
QQP45825.1	(+)	1	GVYYHKNNKSWMESEFRVYSSANNCTFEYVSQPFLMDLEGKQGNFKNLREFVFKNIDGYFKIYSKHTPINLVRDLPQGFSALEP	1,273	Severe acute respiratory...
QMJ01317.1	(+)	1	GVYYHKNNKSWMESEFRVYSSANNCTFEYVSQPFLMDLEGKQGNFKNLREFVFKNIDGYFKIYSKHTPINLVRDLPQGFSALEP	1,273	Severe acute respiratory...
QOH28993.1	(+)	1	GVYYHKNNKSWMESEFRVYSSANNCTFEYVSQPFLMDLEGKQGNFKNLREFVFKNIDGYFKIYSKHTPINLVRDLPQGFSALEP	1,273	Severe acute respiratory...
QPN00919.1	(+)	1	GVYYHKNNKSWMESEFRVYSSANNCTFEYVSQPFLMDLEGKQGNFKNLREFVFKNIDGYFKIYSKHTPINLVRDLPQGFSALEP	1,273	Severe acute respiratory...
QOS49673.1	(+)	1	GVYYHKNNKSWMESEFRVYSSANNCTFEYVSQPFLMDLEGKQGNFKNLREFVFKNIDGYFKIYSKHTPINLVRDLPQGFSALEP	1,273	Severe acute respiratory...
UJE19031.1	(+)	1	GVYYHKNNKSWMESEFRVYSSANNCTFEYVSQPFLMDLEGKQGNFKNLREFVFKNIDGYFKIYSKHTPINLVRDLPQGFSALEP	1,273	Severe acute respiratory...
QNN86445.1	(+)	1	GVYYHKNNKSWMESEFRVYSSANNCTFEYVSQPFLMDLEGKQGNFKNLREFVFKNIDGYFKIYSKHTPINLVRDLPQGFSALEP	1,273	Severe acute respiratory...
QKV39923.1	(+)	1	GVYYHKNNKSWMESEFRVYSSANNCTFEYVSQPFLMDLEGKQGNFKNLREFVFKNIDGYFKIYSKHTPINLVRDLPQGFSALEP	1,273	Severe acute respiratory...
QNA39510.1	(+)	1	GVYYHKNNKSWMESEFRVYSSANNCTFEYVSQPFLMDLEGKQGNFKNLREFVFKNIDGYFKIYSKHTPINLVRDLPQGFSALEP	1,273	Severe acute respiratory...
WJZ59893.1	(+)	1	GVYYHKNNKSWMESEFRVYSSANNCTFEYVSQPFLMDLEGKQGNFKNLREFVFKNIDGYFKIYSKHTPINLVRDLPQGFSALEP	1,273	Severe acute respiratory...
QMX86773.1	(+)	1	GVYYHKNNKSWMESEFRVYSSANNCTFEYVSQPFLMDLEGKQGNFKNLREFVFKNIDGYFKIYSKHTPINLVRDLPQGFSALEP	1,273	Severe acute respiratory...
QJT73034.1	(+)	1	GVYYHKNNKSWMESEFRVYSSANNCTFEYVSQPFLMDLEGKQGNFKNLREFVFKNIDGYFKIYSKHTPINLVRDLPQGFSALEP	1,273	Severe acute respiratory...
WQR41098.1	(+)	1	GVYYHKNNKSWMESEFRVYSSANNCTFEYVSQPFLMDLEGKQGNFKNLREFVFKNIDGYFKIYSKHTPINLVRDLPQGFSALEP	1,273	Severe acute respiratory...
QIZ16559.1	(+)	1	GVYYHKNNKSWMESEFRVYSSANNCTFEYVSQPFLMDLEGKQGNFKNLREFVFKNVDGYFKIYSKHTPINLVRDLPQGFSALEP	1,273	Severe acute respiratory...
QOF10625.1	(+)	1	GVYYHKNNKSWMESEFRVYSSANNCTFEYVSQPFLMDLEGKQGNFKNLREFVFKNIDGYFKIYSKHTPINLVRDLPQGFSALEP	1,273	Severe acute respiratory...
QWC76579.1	(+)	1	GVYYHKNNKSWMESEFRVYSSANNCTFEYVSQPFLMDLEGKQGNFKNLREFVFKNIDGYFKIYSKHTPINLVRDLPQGFSALEP	1,273	Severe acute respiratory...
QSE99577.1	(+)	1	GVYYHKNNKSWMESEFRVYSSANNCTFEYVSQPFLMDLEGKQGNFKNLREFVFKNIDGYFKIYSKHTPINLVRDLPQGFSALEP	1,273	Severe acute respiratory...
QOF08429.1	(+)	1	GVYYHKNNKSWMESEFRVYSSANNCTFEYVSQPFLMDLEGKQGNFKNLREFVFKNIDGYFKIYSKHTPINLVRDLPQGFSALEP	1,273	Severe acute respiratory...
QJS39579.1	(+)	1	GVYYHKNNKSWMESEFRVYSSANNCTFEYVSQPFLMDLEGKQGNFKNLREFVFKNIDGYFKIYSKHTPINLVRDLPQGFSALEP	1,273	Severe acute respiratory...
QOE75686.1	(+)	1	GVYYHKNNKSWMESEFRVYSSANNCTFEYVSQPFLMDLEGKQGNFKNLREFVFKNIDGYFKIYSKHTPINLVRDLPQGFSALEP	1,273	Severe acute respiratory...
QQO76328.1	(+)	1	GVYYHKNNKSWMESEFRVYSSANNCTFEYVSQPFLMDLEGKQGNFKNLREFVFKNIDGYFKIYSKHTPINLVRDLPQGFSALEP	1,273	Severe acute respiratory...
UXB96093.1	(+)	1	GVYYHKNNKSWMESEFRVYSSANNCTFEYVSQPFLMDLEGKQGNFKNLREFVFKNIDGYFKIYSKHTPINLVRDLPQGFSALEP	1,273	Severe acute respiratory...
UYA31504.1	(+)	1	GVYYHKNNKSWMESEFRVYSSANNCTFEYVSQPFLMDLEGKQGNFKNLREFVFKNIDGYFKIYSKHTPINLVRDLPQGFSALEP	1,273	Severe acute respiratory...
QTI95854.1	(+)	1	GVYYHKNNKSWMESEFRVYSSANNCTFEYVSQPFLMDLEGKQGNFKNLREFVFKNIDGYFKIYSKHTPINLVRDLPQGFSALEP	1,273	Severe acute respiratory...
QNJ99456.1	(+)	1	GVYYHKNNKSWMESEFRVYSSANNCTFEYVSQPFLMDLEGKQGNFKNLREFVFKNIDGYFKIYSKHTPINLVRDLPQGFSALEP	1,273	Severe acute respiratory...
QXE71677.1	(+)	1	GVYYHKNNKSWMESEFRVYSSANNCTFEYVSQPFLMDLEGKQGNFKNLREFVFKNIDGYFKIYSKHTPINLVRDLPQGFSALEP	1,273	Severe acute respiratory...
QIT07011.1	(+)	1	GVYYHKNNKSWMESEFRVYSSANNCTFEYVSQPFLMDLEGKQGNFKNLREFVFKNIDGYFKIYSKHTPINLVRDLPQGFSALEP	1,273	Severe acute respiratory...
QMX85049.1	(+)	1	GVYYHKNNKSWMESEFRVYSSANNCTFEYVSQPFLMDLEGKQGNFKNLREFVFKNIDGYFKIYSKHTPINLVRDLPQGFSALEP	1,273	Severe acute respiratory...
QJW70147.1	(+)	1	GVYYHKNNKSWMESEFRVYSSANNCTFEYVSQPFLMDLEGKQGNFKNLREFVFKNIDGYFKIYSKHTPINLVRDLPQGFSALEP	1,273	Severe acute respiratory...
QKU28894.1	(+)	1	GVYYHKNNKSWMESEFRVYSSANNCTFEYVSQPFLMDLEGKQGNFKNLREFVFKNIDGYFKIYSKHTPINLVRDLPQGFSALEP	1,273	Severe acute respiratory...
QXR88761.1	(+)	1	GVYYHKNNKSWMESEFRVYSSANNCTFEYVSQPFLMDLEGKQGNFKNLREFVFKNIDGYFKIYSKHTPINLVRDLPQGFSALEP	1,273	Severe acute respiratory...
QOU94298.1	(+)	1	GVYYHKNNKSWMESEFRVYSSANNCTFEYVSQPFLMDLEGKQGNFKNLREFVFKNIDGYFKIYSKHTPINLVRDLPQGFSALEP	1,273	Severe acute respiratory...
QJS53398.1	(+)	1	GVYYHKNNKSWMESEFRVYSSANNCTFEYVSQPFLMDLEGKQGNFKNLREFVFKNIDGYFKIYSKHTPINLVRDLPQGFSALEP	1,273	Severe acute respiratory...
QOU91202.1	(+)	1	GVYYHKNNKSWMESEFRVYSSANNCTFEYVSQPFLMDLEGKQGNFKNLREFVFKNIDGYFKIYSKHTPINLVRDLPQGFSALEP	1,273	Severe acute respiratory...
QQP47157.1	(+)	1	GVYYHKNNKSWMESEFRVYSSANNCTFEYVSQPFLMDLEGKQGNFKNLREFVFKNIDGYFKIYSKHTPINLVRDLPQGFSALEP	1,273	Severe acute respiratory...
QLC93524.1	(+)	1	GVYYHKNNKSWMESEFRVYSSANNCTFEYVSQPFLMDLEGKQGNFKNLREFVFKNIDGYFKIYSKHTPINLVRDLPQGFSALEP	1,273	Severe acute respiratory...
QWM87761.1	(+)	1	GVYYHKNNKSWMESEFRVYSSANNCTFEYVSQPFLMDLEGKQGNFKNLREFVFKNIDGYFKIYSKHTPINLVRDLPQGFSALEP	1,273	Severe acute respiratory...
QXR89097.1	(+)	1	GVYYHKNNKSWMESEFRVYSSANNCTFEYVSQPFLMDLEGKQGNFKNLREFVFKNIDGYFKIYSKHTPINLVRDLPQGFSALEP	1,273	Severe acute respiratory...
QOF12353.1	(+)	1	GVYYHKNNKSWMESEFRVYSSANNCTFEYVSQPFLMDLEGKQGNFKNLREFVFKNIDGYFKIYSKHTPINLVRDLPQGFSALEP	1,273	Severe acute respiratory...
WAQ38474.1	(+)	1	GVYYHKNNKSWMESEFRVYSSANNCTFEYVSQPFLMDLEGKQGNFKNLREFVFKNIDGYFKIYSKHTPINLVRDLPQGFSALEP	1,273	Severe acute respiratory...
QOU89966.1	(+)	1	GVYYHKNNKSWMESEFRVYSSANNCTFEYVSQPFLMDLEGKQGNFKNLREFVFKNIDGYFKIYSKHTPINLVRDLPQGFSALEP	1,273	Severe acute respiratory...
QOU93974.1	(+)	1	GVYYHKNNKSWMESEFRVYSSANNCTFEYVSQPFLMDLEGKQGNFKNLREFVFKNIDGYFKIYSKHTPINLVRDLPQGFSALEP	1,273	Severe acute respiratory...
QQK89979.1	(+)	1	GVYYHKNNKSWMESEFRVYSSANNCTFEYVSQPFLMDLEGKQGNFKNLREFVFKNIDGYFKIYSKHTPINLVRDLPQGFSALEP	1,273	Severe acute respiratory...
QMI94525.1	(+)	1	GVYYHKNNKSWMESEFRVYSSANNCTFEYVSQPFLMDLEGKQGNFKNLREFVFKNIDGYFKIYSKHTPINLVRDLPQGFSALEP	1,273	Severe acute respiratory...
QLC48052.1	(+)	1	GVYYHKNNKSWMESEFRVYSSANNCTFEYVSQPFLMDLEGKQGNFKNLREFVFKNIDGYFKIYSKHTPINLVRDLPQGFSALEP	1,273	Severe acute respiratory...
QJE38426.1	(+)	1	GVYYHKNNKSWMESEFRVYSSANNCTFEYVSQPFLMDLEGKQGNFKNLREFVFKNIDGYFKIYSKHTPINLVRDLPQGFSALEP	1,273	Severe acute respiratory...
QIS60489.1	(+)	1	GVYYHKNNKSWMESEFRVYSSANNCTFEYVSQPFLMDLEGKQGNFKNLREFVFKNIDGYFKIYSKHTPINLVRDLPQGFSALEP	1,273	Severe acute respiratory...
UCK95537.1	(+)	1	GVYYHKNNKSWMESEFRVYSSANNCTFEYVSQPFLMDLEGKQGNFKNLREFVFKNIDGYFKIYSKHTPINLVRDLPQGFSALEP	1,273	Severe acute respiratory...
QLI51781.1	(+)	1	GVYYHKNNKSWMESEFRVYSSANNCTFEYVSQPFLMDLEGKQGNFKNLREFVFKNIDGYFKIYSKHTPINLVRDLPQGFSALEP	1,273	Severe acute respiratory...
QKV41471.1	(+)	1	GVYYHKNNKSWMESEFRVYSSANNCTFEYVSQPFLMDLEGKQGNFKNLREFVFKNIDGYFKIYSKHTPINLVRDLPQGFSALEP	1,273	Severe acute respiratory...
QKY78084.1	(+)	1	GVYYHKNNKSWMESEFRVYSSANNCTFEYVSQPFLMDLEGKQGNFKNLREFVFKNIDGYFKIYSKHTPINLVRDLPQGFLALEP	1,273	Severe acute respiratory...
UHX58263.1	(+)	1	GVYYHKNNKSWMESEFRVYSSANNCTFEYVSKPFLMDLEGKQGNFKNLREFVFKNIDGYFKIYSKHTPINLVRDLPQGFSALEP	1,273	Severe acute respiratory...
QRA18933.1	(+)	1	GVYYHKNNKSWMESEFRVYSSANNCTFEYVSQPFLMDLEGKQGNFKNLREFVFKNIDGYFKIYSKHTPINLVRDLPQGFSALEP	1,273	Severe acute respiratory...
QJR84873.1	(+)	1	GVYYHKNNKSWMESEFRVYSSANNCTFEYVSQPFLMDLEGKQGNFKNLREFVFKNIDGYFKIYSKHTPINLVRDLPQGFSALEP	1,273	Severe acute respiratory...
QWC77885.1	(+)	1	GVYYHKNNKSWMESEFRVYSSANNCTFEYVSQPFLMDLEGKQGNFKNLREFVFKNIDGYFKIYSKHTPINLVRDLPQGFSALEP	1,273	Severe acute respiratory...
QRN63738.1	(+)	1	GVYYHKNNKSWMESEFRVYSSANNCTFEYVSQPFLMDLEGKQGNFKNLREFVFKNIDGYFKIYSKHTPINLVRDLPQGFSALEP	1,273	Severe acute respiratory...
QMT96172.1	(+)	1	GVYYHKNNKSWMESEFRVYSSANNCTFEYVSQPFLMDLEGKQGNFKNLREFVFKNIDGYFKIYSKHTPINLVRDLPQGFSALEP	1,273	Severe acute respiratory...
QJQ39524.1	(+)	1	GVYYHKNNKSWMESEFRVYSSANNCTFEYVSQPFLMDLEGKQGNFKNLREFVFKNIDGYFKIYSKHTPINLVRDLPQGFSALEP	1,273	Severe acute respiratory...
QIU80973.1	(+)	1	GVYYHKNNKSWMESEFRVYSSANNCTFEYVSQPFLMDLEGKQGNFKNLREFVFKNIDGYFKIYSKHTPINLVRDLPQGFSALEP	1,273	Severe acute respiratory...
QOF15989.1	(+)	1	GVYYHKNNKSWMESEFRVYSSANNCTFEYVSQPFLMDLEGKQGNFKNLREFVFKNIDGYFKIYSKHTPINLVRDLPQGFSALEP	1,273	Severe acute respiratory...
QIS30335.1	(+)	1	GVYYHKNNKSWMESEFRVYSSANNCTFEYVSQPFLMDLEGKQGNFKNLREFVFKNIDGYFKIYSKHTPINLVRDLPQGFSALEP	1,273	Severe acute respiratory...
QKU28906.1	(+)	1	GVYYHKNNKSWMESEFRVYSSANNCTFEYVSQPFLMDLEGKQGNFKNLREFVFKNIDGYFKIYSKHTPINLVRDLPQGFSALEP	1,273	Severe acute respiratory...
QRN58518.1	(+)	1	GVYYHKNNKSWMESEFRVYSSANNCTFEYVSQPFLMDLEGKQGNFKNLREFVFKNIDGYFKIYSKHTPINLVRDLPQGFSALEP	1,273	Severe acute respiratory...
QJQ84676.1	(+)	1	GVYYHKNNKSWMESEFRVYSSANNCTFEYVSQPFLMDLEGKQGNFKNLREFVFKNIDGYFKIYSKHTPINLVRDLPQGFSALEP	1,273	Severe acute respiratory...
UGP11912.1	(+)	1	GVYYHKNNKSWMESEFRVYSSANNCTFEYVSQPFLMDLEGKQGNFKNLREFVFKNIDGYFKIYSKHTPINLVRDLPQGFSALEP	1,273	Severe acute respiratory...
QKU33257.1	(+)	1	GVYYHKNNKSWMESEFRVYSSANNCTFEYVSQPFLMDLEGKQGNFKNLKEFVFKNIDGYFKIYSKHTPINLVRDLPQGFSALEP	1,273	Severe acute respiratory...
QJR84837.1	(+)	1	GVYYHKNNKSWMESEFRVYSSANNCTFEYVSQPFLMDLEGKQGNFKNLREFVFKNIDGYFKIYSKHTPINLVRDLPQGFSALEP	1,273	Severe acute respiratory...
QOU86714.1	(+)	1	GVYYHKNNKSWMESEFRVYSSANNCTFEYVSQPFLMDLEGKQGNFKNLREFVFKNIDGYFKIYSKHTPINLVRDLPQGFSALEP	1,273	Severe acute respiratory...
QIS60906.1	(+)	1	GVYYHKNNKSWMESEFRVYSSANNCTFEYVSQPFLMDLEGKQGNFKNLREFVFKNIDGYFKIYSKHTPINLVRDLPQGFSALEP	1,273	Severe acute respiratory...
QKU31481.1	(+)	1	GVYYHKNNKSWMESEFRVYSSANNCTFEYVSQPFLMDLEGKQGNFKNLREFVFKNIDGYFKIYSKHTPINLVRDLPQGFSALEP	1,273	Severe acute respiratory...
QZJ49063.1	(+)	1	GVYYHKNNKSWMESEFRVYSSANNCTFEYVSQPFLMDLEGKQGNFKNLREFVFKNIDGYFKIYSKHTPINLVRDLPQGFSALEP	1,273	Severe acute respiratory...
QJC19455.1	(+)	1	GVYYHKNNKSWMESEFRVYSSANNCTFEYVSQPFLMDLEGKQGNFKNLREFVFKNIDGYFKIYSKHTPINLVRDLPQGFSALEP	1,273	Severe acute respiratory...
QQP44733.1	(+)	1	GVYYHKNNKSWMESEFRVYSSANNCTFEYVSQPFLMDLEGKQGNFKNLREFVFKNIDGYFKIYSKHTPINLVRDLPQGFSALEP	1,273	Severe acute respiratory...
QWJ89677.1	(+)	1	GVYYHKNNKSWMESEFRVYSSANNCTFEYVSQPFLMDLEGKQGNFKNLREFVFKNIDGYFKIYSKHTPINLVRDLPQGFSALEP	1,273	Severe acute respiratory...
QKV35819.1	(+)	1	GVYYHKNNKSWMESEFRVYSSANNCTFEYVSQPFLMDLEGKQGNFKNLREFVFKNIDGYFKIYSKHTPINLVRDLPQGFSALEP	1,273	Severe acute respiratory...
QPF48560.1	(+)	1	GVYYHKNNKSWMESEFRVYSSANNCTFEYVSQPFLMDLEGKQGNFKNLREFVFKNIDGYFKIYSKHTPINLVRDLPQGFSALEP	1,273	Severe acute respiratory...
UFV35855.1	(+)	1	GVYYHKNNKSWMESEFRVYSSANNCTFEYVSQPFLMDLEGKQGNFKNLREFVFKNIDGYFKIYSKHTPINLVRDLPQGFSALEP	1,273	Severe acute respiratory...
QIA98583.1	(+)	1	GVYYHKNNKSWMESEFRVYSSANNCTFEYVSQPFLMDLEGKQGNFKNLREFVFKNIDGYFKIYSKHTPINLVRDLPQGFSALEP	1,273	Severe acute respiratory...
UHH97155.1	(+)	1	GVYYHKNNKSWMESEFRVYSSANNCTFEYVSQPFLMDLEGKQGNFKNLREFVFKNIDGYFKIYSKHTPINLVRDLPQGFSALEP	1,273	Severe acute respiratory...
QIS30165.1	(+)	1	GVYYHKNNKSWMESEFRVYSSANNCTFEYVSQPFLMDLEGKQGNFKNLREFVFKNIDGYFKIYSKHTPINLVRDLPQGFSALEP	1,273	Severe acute respiratory...
QLJ53548.1	(+)	1	GVYYHKNNKSWMESEFRVYSSANNCTFEYVSQPFFMDLEGKQGNFKNLREFVFKNIDGYFKIYSKHTPINLVRDLPQGFSALEP	1,273	Severe acute respiratory...
QJW39952.1	(+)	1	GVYYHKNNKSWMESEFRVYSSANNCTFEYVSQPFLMDLEGKQGNFKNLREFVFKNIDGYFKIYSKHTPINLVRDLPQGFSALEP	1,273	Severe acute respiratory...
QIU81873.2	(+)	1	GVYYHKNNKSWMESEFRVYSSANNCTFEYVSQPFLMDLEGKQGNFKNLREFVFKNIDGYFKIYSKHTPINLVRDLPQGFSALEP	1,273	Severe acute respiratory...
QOU93902.1	(+)	1	GVYYHKNNKSWMESEFRVYSSANNCTFEYVSQPFLMDLEGKQGNFKNLREFVFKNIDGYFKIYSKHTPINLVRDLPQGFSALEP	1,273	Severe acute respiratory...
