# Supplementary material for: Design, Development and Immunogenicity Study of a Multi-Epitope Vaccine Prototype Against SARS-CoV-2
Source: Pharmaceuticals (Basel). 2024 Nov 7;17(11):1498. doi: 10.3390/ph17111498 (PMC11597159; doi:10.3390/ph17111498)
Supplement: Supplementary file 1 [file Table_S8_E_protein_alignment.pdf › Table_S8_E_protein_alignment.pdf]

NCBI Multiple Sequence Alignment Viewer, Version 1.25.2

Sequence ID	Start	Alignment	End	Organism
YP_009825054.1 (+)	1	MYSFVSEETGTLIVNSVLLFLAFVVFLLVTLAILTALRLCAYCCNIVNVSLVKPTVYVYSRVKNLNSSEGVPDLLV	76	SARS coronavirus Tor2
ADE34757.1 (+)	1	MYSFVSEETGTLIVNSVILFLAFVVFLLVTLAILTALRLCAYCCNIVNVSLVKPTVYVYSRVKNLNSSEGVPDLLV	76	Bat SARS coronavirus ...
AGZ48809.1 (+)	1	MYSFVSEETGTLIVNSVLLFLAFVVFLLVTLAILTALRLCAYCCNIVNVSLVKPTVYVYSRVKNLNSSQGVPDLLV	76	Bat SARS-like coronavir...
AAP51230.1 (+)	1	MYSFVSEETGTLIVNSVLLFLAFMVFLLVTLAILTALRLCAYCCNIVNVSLVKPTVYVYSRVKNLNSSEGVPDLLV	76	SARS coronavirus GD01
ATO98160.1 (+)	1	MYSFVSEETGTLIVNSVLLFLAFVVFLLVTLAILTALRLCAYCCNIVNVSLVKPTVYVYSRVKNLNSAEGVPDLLV	76	Bat SARS-like coronavirus
AIA62312.1 (+)	1	MYSFVSEETGTLIVNSVLLFVAFVVFLLVTLAILTALRLCAYCCNIVNVSLVKPTVYVYSRVKNLNSSEGVPDLLV	76	BtRs-BetaCoV/HuB2013
ACB69908.1 (+)	1	MYSFVSEETGTLILNSVLLFLAFVVFLLVTLAILTALRLCAYCCNIVNVSLVKPTVYVYSRVKNLNSSEGVPDLLV	76	SARS coronavirus BJ1...
ACZ72023.1 (+)	1	MFSFVSEETGTLIVNSVLLFLAFVVFLLVTLAILTALRLCAYCCNIVNVSLVKPTVYVYSRVKNLNSSEGVPDLLV	76	SARS coronavirus ExoN1
Q0Q473.1 (+)	1	MYSFVSEETGTLIVNSVLLFFAFVVFLLVTLAILTALRLCAYCCNIVNVSLVKPTVYVYSRVKNLNSSEGVPDLLV	76	Bat CoV 279/2005
ADC35514.1 (+)	1	MYSFVSEETGTLIVNSVLFFLAFVVFLLVTLAILTALRLCAYCCNIVNVSLVKPTVYVYSRVKNLNSSEGVPDLLV	76	SARS coronavirus HKU...
ADE34768.1 (+)	1	MYSFVSEETGTLIVNSVILFLAFVVFLLVTIAILTALRLCAYCCNIVNVSLVKPTVYVYSRVKNLNSSEGVPDLLV	76	Bat SARS coronavirus ...
AHX37560.1 (+)	1	MYSFVSEETGTLIVNSVLLFLAFVVFLLVTLAILTALRLCAYCCNIVNVSLVKPTVYVYSRVKNLSSSEGVPDLLV	76	Rhinolophus affinis coro...
AKZ19089.1 (+)	1	MYSFVSEETGTLIVNSVLLFLAFVVFLLVALAILTALRLCAYCCNIVNVSLVKPTVYVYSRVKNLNSSEGVPDLLV	76	Bat SARS-like coronavir...
AAR23255.1 (+)	1	MYSFFSEETGTLIVNSVLLFLAFVVFLLVTLAILTALRLCAYCCNIVNVSLVKPTVYVYSRVKNLNSSEGVPDLLV	76	SARS coronavirus Sino3-1
AIA62280.1 (+)	1	MYSFVSEETGTLIVNSVLLFVAFVVFLLVTLAILTALRLCAYCCNIVNVSLVKPTVYVYSRVKNLNSAEGVPDLLV	76	BtRf-BetaCoV/JL2012
ACZ72198.1 (+)	1	MYSFVSEETGTLIVNSVLLFLAFVVFLLVTLAILTAHRLCAYCCNIVNVSLVKPTVYVYSRVKNLNSSEGVPDLLV	76	SARS coronavirus ExoN1
ACZ72243.1 (+)	1	MYSFVSEETGTLIVNSVLLFLAFVVFLLVTLAILTALRLCAYCCNIVNVSLVKPTVYVYSRVKNLNSFEGVPDLLV	76	SARS coronavirus MA...
ACZ72168.1 (+)	1	MYSFVSEETGTLIVNSVLLFLAFVXFLLVTLAILTALRLCAYCCNIVNVSLVKPTVYVYSRVKNLNSSEGVPDLL	75	SARS coronavirus ExoN1
WEG19433.1 (+)	1	MYSFVSEETGTLIVNSVLLFLAFVVFLLVTLAILTALRLCAYCCNIVNVSLVKPTVYVYSRVKNLNSSERVPDLLV	76	Severe acute respiratory...
ABM92862.1 (+)	1	MYSFVSEETGTLIVNSVLLFLAFVVFLLVILAILTALRLCAYCCNIVNVSLVKPTVYVHSRVKNLNSSEGVPDLLV	76	SARS coronavirus TJ01
ATO98184.1 (+)	1	MYSFVSEETGTLIVNSVLLFLAFVVFLLVTLAILTALRLCAYCCNIVNVSLVKPTVYVYSRVKNLNSSEGVLDLLV	76	Bat SARS-like coronavirus
ACZ72257.1 (+)	1	MYSFVSEETGTLIVNSVLLFLAFVVFLLVTLAILTALRLCACCCNIVNVSLVKPTVYVYSRVKNLNSSEGVPDLLV	76	SARS coronavirus ExoN1
APO40581.1 (+)	1	MYSFVSEETGTLIVNSVLLFLAFVVFLLVTLAILTALRLCAYCCNIVNVSLVKPSFYIYSRVKNLNSSQGIPDLLV	76	Severe acute respiratory...
AGC74167.1 (+)	1	MYSFVSEETGTLIVNSVLLFFAFVVFLLVTLAILTALRLCAYCCNIVNVSLVKPTVYVYSRVKNLNSSVGVPDLLV	76	Bat coronavirus Rp/Sha...
AAS44718.1 (+)	1	MYSFVSEETGTLI-NSVLLFLAFVVFLLVTLAILTALRLCAYCCNIVNVSLVKPTVYVYSRVKNLNSSEGVPDLLV	75	SARS coronavirus TW-...
ABD75313.1 (+)	1	MYSFVSEETGTLIVNSVLLFVAFVVFLLVTLAILTALRLCAYCCNIVNVSLVKPTVYVYSRVKNLNSSDCVPDLLV	76	Bat SARS CoV Rf1/2004
AIA62302.1 (+)	1	MYSFVSEETGTLIVNSVLLFVAFVVFLLVTLAILTALRLCAYCCNIVNVSLVKPTVYVYSRVKNMNSSDCVPDLLV	76	BtRf-BetaCoV/SX2013
AAR23247.1 (+)	1	MYSFVSEETGTLI-NSVLLFLAFVVFLLVTLAILTALRLCAYCCYIVNVSLVKPTVYVYSRVKNLNSSEGVPDLLV	75	SARS coronavirus Sino1-1
AAS48454.1 (+)	1	MYSFVSEETGTLIVNSVLPFLAFVVFLLAILAILTALRLCAYCCNIVNVSLVKPTVYVYSRVKNLNSSEGVSDLLV	76	SARS coronavirus BJ01
QQN96057.1 (+)	1	MYSFVSEETGTLIVNSVLLFLAFVVFLLVTLAILTALRLCAYCCNIVNVSLVKPSFYVYSRVKNLNSS-GVPDLLV	75	Severe acute respiratory...
URA79942.1 (+)	1	MYSFVSEETGTLIVNSVLLFLAFVVFLLVTLAILTALRLCAYCCNIVNVSLVKPSVYVYSRVKNLNSS-RVPDLLV	75	Severe acute respiratory...
UDK72781.1 (+)	1	MYSFVSEETGTLIVNSVLLFLAFVVFLLVTLAILTALRLCAYCCNIVNVSLVKPSFYVYSRVKNLNSS-RGPDLLV	75	Severe acute respiratory...
UFD49709.1 (+)	1	MYSFVSEETGTLIVNSVLLFLAFVVFLLVTLAILTALRLCAYCCNIVNVSLVKPSIYVYSRVKNLNSS-RVPDLLV	75	Severe acute respiratory...
QRJ61691.1 (+)	1	MYSFVSEETGTLIVNSVLLFLAFVVFLLVTLAILTALRLCAYCCNIVNVSLVKPSLYVYSRVKNLNSS-RVPDLLV	75	Severe acute respiratory...
QVI46785.1 (+)	1	MYSFVSEETGTLIVNSVLLFLAFVVFLLVTLAILTALRLCAYCCNIVNVSLVKPSXYVYSRVKNLNSS-RVPDLLV	75	Severe acute respiratory...
QRX24290.1 (+)	1	MYSFVSEETGTLIVNSVLLFLAFVVFLLVTLAILTALRLCAYCCNIVNVSLVKPSFYVYSRVKNLNSS-KVPDLLV	75	Severe acute respiratory...
ULL96160.1 (+)	1	MYSFVSEEIGTLIVNSVLLFLAFVVFLLVTLAILTALRLCAYCCNIVNVSLVKPSFYVYSRVKNLNSS-GVPDLLV	75	Severe acute respiratory...
YP_009724392.1 (+)	1	MYSFVSEETGTLIVNSVLLFLAFVVFLLVTLAILTALRLCAYCCNIVNVSLVKPSFYVYSRVKNLNSS-RVPDLLV	75	Severe acute respiratory...
QZC48420.1 (+)	1	MYSFVSEETGTLIVNSVLLFLAFVVFLLVTLAILTALRLCAYCCNIVBVSLVKPSFYVYSRVKNLNSS-RVPDLLV	75	Severe acute respiratory...
UFR59131.1 (+)	1	MYSFVSEETGTLIVNSVLLFLAFVVFLLVTLAILTALRLCAYCCBIVNVSLVKPSFYVYSRVKNLNSS-RVPDLLV	75	Severe acute respiratory...
QTT14430.1 (+)	1	MYSFVSEETGTLIVNSVLLFLAFVVFLLVTLAILTALRLCAYCCNIVNVSLVKPSFYVYSRVKNLNSS-SVPDLLV	75	Severe acute respiratory...
ULT67074.1 (+)	1	MYSFVSEEIGTLIVNSVLLFLAFVVFLLVTLAILTALRLCAYCCNIVNVSLVKPSVYVYSRVKNLNSS-RVPDLLV	75	Severe acute respiratory...
UDL46515.1 (+)	1	MYSFVSEETGTLIVNSVLLFLAFVVFLLVTLAILTALRLCAYCCNIVNVSLVKPSSYVYSRVKNLNSS-RVPDLLV	75	Severe acute respiratory...
QSX85891.1 (+)	1	MYSFVSEETGTLIVNSVLLFLAFVVFLLVTLAILTALRLCAYCCNIVNVSLVKPSYYVYSRVKNLNSS-RVPDLLV	75	Severe acute respiratory...
UQF05356.1 (+)	1	MYSFVSEETGTLIVNSVLLFLAFVVFLLVTLAIJTALRLCAYCCNIVNVSLVKPSFYVYSRVKNLNSS-RVPDLLV	75	Severe acute respiratory...
UHO77330.1 (+)	1	MYSFVSEETGTLIVNSVJLFLAFVVFLLVTLAILTALRLCAYCCNIVNVSLVKPSFYVYSRVKNLNSS-RVPDLLV	75	Severe acute respiratory...
UFK01962.1 (+)	1	MYSFVSEETGTLIVNSVLJFLAFVVFLLVTLAILTALRLCAYCCNIVNVSLVKPSFYVYSRVKNLNSS-RVPDLLV	75	Severe acute respiratory...
UGK66263.1 (+)	1	MYSFVSEETGTLIVNSVLLFJAFVVFLLVTLAILTALRLCAYCCNIVNVSLVKPSFYVYSRVKNLNSS-RVPDLLV	75	Severe acute respiratory...
QPK75943.1 (+)	1	MYSFVSEETGTLIVNSVLLFLAFVVFLLVTLAILTALRLCAYCCNIVNVSJVKPSFYVYSRVKNLNSS-RVPDLLV	75	Severe acute respiratory...
QUE12501.1 (+)	1	MYSFVSEETGTLIVNSILLFLAFVVFLLVTLAILTALRLCAYCCNIVNVSLVKPSFYVYSRVKNLNSS-RVPDLLV	75	Severe acute respiratory...
QWQ62957.1 (+)	1	MYSFVSEETGTLIVNSVLLFLAFVVFLLVTLAILTALRLCAYCCNIINVSLVKPSFYVYSRVKNLNSS-RVPDLLV	75	Severe acute respiratory...
UHK01702.1 (+)	1	MYSFVSEETGTLIVNSVLLFLAFVIFLLVTLAILTALRLCAYCCNIVNVSLVKPSFYVYSRVKNLNSS-RVPDLLV	75	Severe acute respiratory...
QRG50750.1 (+)	1	MYSFVSEETGTLIVNSVLLFLAFVVFLLVTLAILTALRLCAYCCNIVNVSLVKPSFYIYSRVKNLNSS-RVPDLLV	75	Severe acute respiratory...
QQZ31248.1 (+)	1	MYSFVSEETGTLIVNSVLLFLAFVVFLLVTLAILTALRLCAYCCNIVNVSLIKPSFYVYSRVKNLNSS-RVPDLLV	75	Severe acute respiratory...
QRK19029.1 (+)	1	MYSFVSEETGTLIVNSVLLFLAFVVFLLVTLAILTALRLCAYCCNIVNVSLVKPSFYVYSRVKNLNSS-RIPDLLV	75	Severe acute respiratory...
QQX20997.1 (+)	1	MYSFVSEETGTLIVNSVLLFLAFVVFLLVTLAILTALRLCAYCCNIVNVSLVKPSFYVYSRIKNLNSS-RVPDLLV	75	Severe acute respiratory...
QLH58110.1 (+)	1	MYSFISEETGTLIVNSVLLFLAFVVFLLVTLAILTALRLCAYCCNIVNVSLVKPSFYVYSRVKNLNSS-RVPDLLV	75	Severe acute respiratory...
QSG29993.1 (+)	1	MYSFVSEETGTLIINSVLLFLAFVVFLLVTLAILTALRLCAYCCNIVNVSLVKPSFYVYSRVKNLNSS-RVPDLLV	75	Severe acute respiratory...
QSG72893.1 (+)	1	MYSFVSEETGTLIVNSVLLFLAFVVFLLVTLAILTALRLCAYCCNIVNVSLVKPSFYVYSRVKNLNSS-RVPDLLI	75	Severe acute respiratory...
QTS38927.1 (+)	1	MYSFVSEETGTLIVNSVLLFLAFVVFLLITLAILTALRLCAYCCNIVNVSLVKPSFYVYSRVKNLNSS-RVPDLLV	75	Severe acute respiratory...
UEK36518.1 (+)	1	MYSFVSEETGTLIVNSVLLFLAFVVFLLVTLAILTALRLCAYCCNIVNVSLVKPAFYVYSRVKNLNSS-RVPDLLV	75	Severe acute respiratory...
QRG35278.1 (+)	1	MYSFVSEETGTLIVNSVLLFLAFVVFLLVTLAILTALRLCAYCCNIVNVSLVKPSFYVYSRVKNMNSS-RVPDLLV	75	Severe acute respiratory...
QNO87829.1 (+)	1	MYSFVSEETGTLIVNSVLLFLAFVVFLLVTLAILTALRLCAYCCNIVNVSLVKPSFYVYSRVKNLNSS-RVPDLMV	75	Severe acute respiratory...
QJS53352.1 (+)	1	MYSFVSEETGTLIVNSVLLFLAFVVFLLVTLAILTALRMCAYCCNIVNVSLVKPSFYVYSRVKNLNSS-RVPDLLV	75	Severe acute respiratory...
UEN00206.1 (+)	1	MYSFVSEETGTLIVNSVLLFLAFVVFLLVTLAILTALRLCAYCCNIVNVSLAKPSLYVYSRVKNLNSS-RVPDLLV	75	Severe acute respiratory...
QRW50543.1 (+)	1	MYSFVSEETGTLIVNSVLLFLAFVVFLLVTLAILTALRLCAYCCNIVNVSLVKPSFYVYSRVKNLNSS-TVPDLLV	75	Severe acute respiratory...
QRN44206.1 (+)	1	MYSFVSEETGTLIVNSVLLFLAFVVFLLVTLAILTALRLCAYCCNIVNVSLVKPSFYVYSRVKNLNSS-RAPDLLV	75	Severe acute respiratory...
UAZ11634.1 (+)	1	MYSFVSEETGTLIVNSVLLFLAFVVFLLVTLAILTALRLCAYCCNIVNVSLVKPXXYVYSRVKNLNSS-RVPDLLV	75	Severe acute respiratory...
UGL31825.1 (+)	1	MYSFVSEETGTLIVNSVLLFLAFVVFLLVTLAVLTALRLCAYCCNIVNVSLVKPSFYVYSRVKNLNSS-RVPDLLV	75	Severe acute respiratory...
QPF50380.1 (+)	1	MYSFVSEETGTLIVNSVLLFLAFVVFLLVTLAILTALRLCAYCCNIVNVSLVKPSFYVYSRVKNLNSS-XVPDLLV	75	Severe acute respiratory...
QOS50895.1 (+)	1	MYSFVSEETGTLIVNSVLLFLAFVVFLLVTLAILTALRLCAYCCNVVNVSLVKPSFYVYSRVKNLNSS-RVPDLLV	75	Severe acute respiratory...
QSM10526.1 (+)	1	MYSFVSEETGTLVVNSVLLFLAFVVFLLVTLAILTALRLCAYCCNIVNVSLVKPSFYVYSRVKNLNSS-RVPDLLV	75	Severe acute respiratory...
UAG57581.1 (+)	1	MYSFVSEETGTLIVNSVLLFLAFVVFLLVTLAIITALRLCAYCCNIVNVSLVKPSFYVYSRVKNLNSS-RVPDLLV	75	Severe acute respiratory...
UCB79167.1 (+)	1	MYSFVSEETGTLIVNSVLLFLAFVVFLIVTLAILTALRLCAYCCNIVNVSLVKPSFYVYSRVKNLNSS-RVPDLLV	75	Severe acute respiratory...
QTI97232.1 (+)	1	MYSFVSEETGTLIVNSVILFLAFVVFLLVTLAILTALRLCAYCCNIVNVSLVKPSFYVYSRVKNLNSS-RVPDLLV	75	Severe acute respiratory...
QSM17398.1 (+)	1	MYSFVSEETGTLIVNSVLIFLAFVVFLLVTLAILTALRLCAYCCNIVNVSLVKPSFYVYSRVKNLNSS-RVPDLLV	75	Severe acute respiratory...
QVD65749.1 (+)	1	MYSFVSEETGTLIVNSVLLFLAFVVFLLVTIAILTALRLCAYCCNIVNVSLVKPSFYVYSRVKNLNSS-RVPDLLV	75	Severe acute respiratory...
QTM77609.1 (+)	1	MYSFVSEETGTLIVNSVLLFLAFVVFLLVTLAILTALRLCAYCCNIVNVSIVKPSFYVYSRVKNLNSS-RVPDLLV	75	Severe acute respiratory...
QVJ16632.1 (+)	1	MYSFVSEETGTLIVNSVLLFIAFVVFLLVTLAILTALRLCAYCCNIVNVSLVKPSFYVYSRVKNLNSS-RVPDLLV	75	Severe acute respiratory...
QTY59441.1 (+)	1	MYSFVSEETGTLIVNSVLLFLAFVVFLLVTLAILTALRLCAYCCNIVNVSLVKPSFYVYSRVKNLNSS-RVPDILV	75	Severe acute respiratory...
QPM28696.1 (+)	1	MYSFVSEETGTLIVNSVLLFLAFVVFLLVTLAILTALRLCAYCCNIVNVSLVKPXFYVYSRVKNLNSS-RVPDLLV	75	Severe acute respiratory...
QVQ68251.1 (+)	1	MYSFVSEETGTLJVNSVLLFLAFVVFLLVTLAILTALRLCAYCCNIVNVSLVKPSFYVYSRVKNLNSS-RVPDLLV	75	Severe acute respiratory...
QVF67304.1 (+)	1	MYSFVSEQTGTLIVNSVLLFLAFVVFLLVTLAILTALRLCAYCCNIVNVSLVKPSFYVYSRVKNLNSS-RVPDLLV	75	Severe acute respiratory...
QKU52835.1 (+)	1	MYSFVSQETGTLIVNSVLLFLAFVVFLLVTLAILTALRLCAYCCNIVNVSLVKPSFYVYSRVKNLNSS-RVPDLLV	75	Severe acute respiratory...
QZI16545.1 (+)	1	MYSFVSEETGTLIVNSVLLFLAFVVFLLVTLAILTALRLCAYCCNIVNVSLVKPSFYVYSRVRNLNSS-RVPDLLV	75	Severe acute respiratory...
QRW67958.1 (+)	1	MYSFVSEETGTLIVNSVLLFLAFVVFLLVTLAILTALRLCAYCCNIVNVSLVRPSFYVYSRVKNLNSS-RVPDLLV	75	Severe acute respiratory...
QSH79286.1 (+)	1	MYSFVSEETGTLMVNSVLLFLAFVVFLLVTLAILTALRLCAYCCNIVNVSLVKPSFYVYSRVKNLNSS-RVPDLLV	75	Severe acute respiratory...
QOU92764.1 (+)	1	MYSFVSEETGTLIVNSVLLFLAFVVFLLVTLAILTALRLCAYCCNIVNMSLVKPSFYVYSRVKNLNSS-RVPDLLV	75	Severe acute respiratory...
QSD56678.1 (+)	1	MYSFVSEETGTLIVNSVLLFLAFVVFLLVTLAILTALRLCAYCCNIVNVSLVKPSFYVYARVKNLNSS-RVPDLLV	75	Severe acute respiratory...
QRJ37649.1 (+)	1	MYSFVSEETGTLIVNSVLLFLAFVVFLLVTLAILTALRLCAYCCNIVNVSLVKPSFYVYSRVKNLNAS-RVPDLLV	75	Severe acute respiratory...
UBN79421.1 (+)	1	MYAFVSEETGTLIVNSVLLFLAFVVFLLVTLAILTALRLCAYCCNIVNVSLVKPSFYVYSRVKNLNSS-RVPDLLV	75	Severe acute respiratory...
QTS81864.1 (+)	1	MYSFVSEETGTLIVNSVLLFLAFVVFLLVTLAILTALRLCAYCCNIVNVSLVKPSFYVYSRVKNLNSA-RVPDLLV	75	Severe acute respiratory...
QOU93100.1 (+)	1	MYSFVSEETGTLIVNSVLLFLAFMVFLLVTLAILTALRLCAYCCNIVNVSLVKPSFYVYSRVKNLNSS-RVPDLLV	75	Severe acute respiratory...
QUD33741.1 (+)	1	MYSFVAEETGTLIVNSVLLFLAFVVFLLVTLAILTALRLCAYCCNIVNVSLVKPSFYVYSRVKNLNSS-RVPDLLV	75	Severe acute respiratory...
UIB98784.1 (+)	1	MYSFVSEETGTLIVNSVJLFLAFVVFLLVTLAILTALRLCAYCCNIVNVSLVKPSFYVYSRVKNLNSS-XVPDLLV	75	Severe acute respiratory...
QRX21762.1 (+)	1	MYSFVSEETGTLLVNSVLLFLAFVVFLLVTLAILTALRLCAYCCNIVNVSLVKPSFYVYSRVKNLNSS-RVPDLLV	75	Severe acute respiratory...
QTZ61550.1 (+)	1	MYSFVTEETGTLIVNSVLLFLAFVVFLLVTLAILTALRLCAYCCNIVNVSLVKPSFYVYSRVKNLNSS-RVPDLLV	75	Severe acute respiratory...
QVF66436.1 (+)	1	MYSFVSEETGTLIVNTVLLFLAFVVFLLVTLAILTALRLCAYCCNIVNVSLVKPSFYVYSRVKNLNSS-RVPDLLV	75	Severe acute respiratory...
UHI22519.1 (+)	1	MYSFVSEETGTLIVNSVLLFLAFVVFLLVTLAILTALRLCAYCCNIVNVSLVKPSFYVYSRVKNLNTS-RVPDLLV	75	Severe acute respiratory...
QVU41150.1 (+)	1	MYSFVSEETGTLIVNSVLLFLAFVVFLLVTLAILTALRLCAYCCNIVNVSLVKPSFYVYTRVKNLNSS-RVPDLLV	75	Severe acute respiratory...
