# Supplementary material for: Design, Development and Immunogenicity Study of a Multi-Epitope Vaccine Prototype Against SARS-CoV-2
Source: Pharmaceuticals (Basel). 2024 Nov 7;17(11):1498. doi: 10.3390/ph17111498 (PMC11597159; doi:10.3390/ph17111498)
Supplement: Supplementary file 1 [file Table_S9_M_protein_alignment.pdf › Table_S9_M_protein_alignment.pdf]

NCBI Multiple Sequence Alignment Viewer, Version 1.25.2

Sequence ID	Start	Alignment	End	Organism
8CTK_A (+)	1	VLAAVYRINWITGGIAIAMACLVGLMWLSYFIASFRLFARTRSMWSFNPETNILLNVPLHGTILTRPLLESELVIGAVILRGHLRIAGHHLGRCDIKDLPKEITVATSRTL	231	Severe acute respiratory...
YP_009724393.1 (+)	1	VLAAVYRINWITGGIAIAMACLVGLMWLSYFIASFRLFARTRSMWSFNPETNILLNVPLHGTILTRPLLESELVIGAVILRGHLRIAGHHLGRCDIKDLPKEITVATSRTL	222	Severe acute respiratory...
QOI60349.1 (+)	1	VLAAVYRINWVTGGIAIAMACLVGLMWLSYFIASFRLFARTRSMWSFNPETNILLNVPLHGTILTRPLLESELVIGAVILRGHLRIAGHHLGRCDIKDLPKEITVATSRTL	222	Severe acute respiratory...
QRX47137.1 (+)	1	VLAAVYRINWITGGIAVAMACLVGLMWLSYFIASFRLFARTRSMWSFNPETNILLNVPLHGTILTRPLLESELVIGAVILRGHLRIAGHHLGRCDIKDLPKEITVATSRTL	222	Severe acute respiratory...
QRW77293.1 (+)	1	VLAAVYRINWITGGIAIAMACLVGLMWLSYFIASFRLFARTRSMWSFNPETNILLNVPLHGTILTRPLLESELVIGAVILRGHLRIAGHHLGRCDIKDLPKEITVATSRTL	222	Severe acute respiratory...
QOF16748.1 (+)	1	VLAAVYRINWITGGIAIAMACLVGLMWLSYFIASFRLFARTRSMWSFNPETNILLNVPLHGTILTRPLLESELVIGAVILRGHLRIAGHHLGRCDIKDLPKEITVATSRTL	222	Severe acute respiratory...
QRX28077.1 (+)	1	VLAAVYRINWITGGIAIAMACLVGLMWLSYFIASFRLFARTRSMWSFNPETNILLNVPLHGTILTRPLLESELVIGAVILRGHLRIAGHHLGRCDIKDLPKEITVATSRTL	222	Severe acute respiratory...
QUN75372.1 (+)	1	VLAAVYRINWITGGVAIAMACLVGLMWLSYFIASFRLFARTRSMWSFNPETNILLNVPLHGTILTRPLLESELVIGAVILRGHLRIAGHHLGRCDIKDLPKEITVATSRTL	222	Severe acute respiratory...
QRX46158.1 (+)	1	VLAAVYRINWITGGIAIAMACLVGLMWLSYFIASFRLFARTRSMWSFNPETNILLNVPLHGTILTRPLLESELVVGAVILRGHLRIAGHHLGRCDIKDLPKEITVATSRTL	222	Severe acute respiratory...
QOQ01737.1 (+)	1	VLAAVYRINWITGGIAIAMACLVGLMWLSYFVASFRLFARTRSMWSFNPETNILLNVPLHGTILTRPLLESELVIGAVILRGHLRIAGHHLGRCDIKDLPKEITVATSRTL	222	Severe acute respiratory...
QNL10877.1 (+)	1	VLAAVYRINWITGGIAIAMACLVGLMWLSYFIASFRLFARTRSMWSFNPETNILLNVPLHGTVLTRPLLESELVIGAVILRGHLRIAGHHLGRCDIKDLPKEITVATSRTL	222	Severe acute respiratory...
QRW49026.1 (+)	1	VLAAVYRINWITGGIAIAMACLVGLMWLSYFIASFRLFARTRSMWSFNPETNILLNVPLHGTILTRPLLESELVIGAVILRGHLRIAGHHLGRCDVKDLPKEITVATSRTL	222	Severe acute respiratory...
QRX21259.1 (+)	1	VLAAVYRINWITGGIAIAMACLVGLMWLSYFIASFRLFARTRSMWSFNPETNILLNVPLHGTILTRPLLESELVIGAVILRGHLRIAGHHLGRCDIKDLPKEITVATSRTL	222	Severe acute respiratory...
QTS65844.1 (+)	1	VLAAVYRINWITGGIAIAMACLVGLMWLSYFIASFRLFARTRSMWSFNPETNILLNVPLHGTILTRPLLESELVIGAVILRGHLRVAGHHLGRCDIKDLPKEITVATSRTL	222	Severe acute respiratory...
QTI94310.1 (+)	1	VLAAVYRVNWITGGIAIAMACLVGLMWLSYFIASFRLFARTRSMWSFNPETNILLNVPLHGTILTRPLLESELVIGAVILRGHLRIAGHHLGRCDIKDLPKEITVATSRTL	222	Severe acute respiratory...
QQV35322.1 (+)	1	VLAAVYRINWITGGIAIAMACLVGLMWLSYFIASFRLFARTRSMWSFNPETNILLNVPLHGTILTRPLLESELVIGAVILRGHLRIAGHHLGRCDIKDLPKEITVATSRTL	222	Severe acute respiratory...
QTJ91672.1 (+)	1	VLAAVYRINWITGGIAIAMACLVGLMWLSYFIASFRLFARTRSMWSFNPETNILLNVPLHGTILTRPLLESELVIGAVILRGHLRIAGHHLGRCDIKDLPKEVTVATSRTL	222	Severe acute respiratory...
UBG97710.1 (+)	1	VLAAVYRINWITGGIAIAMACLVGLMWLSYFIASFRLFARTRSMWSFNPETNVLLNVPLHGTILTRPLLESELVIGAVILRGHLRIAGHHLGRCDIKDLPKEITVATSRTL	222	Severe acute respiratory...
QQD88113.1 (+)	1	VLAAVYRINWITGGIAIAMACLVGLMWLSYFIASFRLFARTRSMWSFNPETNILLNVPLHGTILTRPLLESELVIGAVVLRGHLRIAGHHLGRCDIKDLPKEITVATSRTL	222	Severe acute respiratory...
QVY50055.1 (+)	1	VLAAVYRINWITGGIAIAMACLVGLMWLSYFIASFRLFARTRSMWSFNPETNILLNVPLHGTILTRPLLESELVIGAVILRGHLRIAGHHLGRCDIKDLPKEITVATSRTL	222	Severe acute respiratory...
QQE72579.1 (+)	1	VLAAVYRINWITGGIAIAMACLVGLMWLSYFIASFRLFARTRSMWSFNPETNILLNVPLHGTILTRPLLESELVIGAVILRGHLRIAGHHLGRCDIKDLPKEITVATSRTL	222	Severe acute respiratory...
QRV65674.1 (+)	1	VLAAVYRINWITGGIAIAMACLVGLMWLSYFIASFRLFARTRSMWSFNPETNILLNVPLHGTILTRPLLESELVIGAVILRGHLRIAGHHLGRCDIKDLPKEITVATSRTL	222	Severe acute respiratory...
UOK91796.1 (+)	1	VLAAVYRINWITGGIAIAMACLVGLMWLSYFIASFRLFARTRSMWSFNPETNILLNVPLHGTILTRPLLESELVIGAVILRGHLRIAGHHLGRCDIKDLPKZITVATSRTL	222	Severe acute respiratory...
QJR92940.1 (+)	1	VLAAVYRINWITGGIAIAMACLVGLMWLSYFIASFRLFARTRSMWSFBPETNILLNVPLHGTILTRPLLESELVIGAVILRGHLRIAGHHLGRCDIKDLPKEITVATSRTL	222	Severe acute respiratory...
QMU25206.1 (+)	1	VLAAVYRINWITGGIAIAMACLVGLMWLSYFIASFRLFARTRSMWSFNPETNILLNVPLHGTILTRPLLESELVIGAVILRGHLRIAGHHLGRCDIKDLPKEITVATSRTL	222	Severe acute respiratory...
QOH27381.1 (+)	1	VLAAVYRINWITGGIAIAMACLVGLMWLSYFIASFRLFARTRSMWSFNPETNILLNVPLHGTILTRPLLESELIIGAVILRGHLRIAGHHLGRCDIKDLPKEITVATSRTL	222	Severe acute respiratory...
QKR85732.1 (+)	1	VLAAVYRINWITGGIAIAMACLVGLMWLSYFIASFRLFARTRSMWSFNPETNILLNVPLHGTILTRPLLESELVIGAVILRGHLRIAGHHLGRCDIKDLPKEITIATSRTL	222	Severe acute respiratory...
QQG80949.1 (+)	1	VLAAVYRINWITGGIAIAMACLVGLMWLSYFIASFRLFARTRSMWSFNPETNILLNVPLHGTILTRPLLESELVIGAVILRGHLRIAGHHLGRCDIKDLPKEITVATSRTL	222	Severe acute respiratory...
QVG38620.1 (+)	1	VLAAVYRINWITGGIAIAMACLVGLMWLSYFIASFRLFARTRSMWSFNPETNILLNVPLHGTILTRPLLESELVIGAVILRGHLRIAGHHLGRCDIKDLPKEITVATSRTL	222	Severe acute respiratory...
QTQ44843.1 (+)	1	VLAAVYRINWITGGIAIAMACLIGLMWLSYFIASFRLFARTRSMWSFNPETNILLNVPLHGTILTRPLLESELVIGAVILRGHLRIAGHHLGRCDIKDLPKEITVATSRTL	222	Severe acute respiratory...
QSS85706.1 (+)	1	VLAAVYRINWITGGIAIAMACLVGLMWLSYFIASFRLFARTRSMWSFNPETNILLNVPLHGTILTRPLLESELVIGAVILRGHLRIAGHHLGRCDIKDLPKEITVATSRTL	222	Severe acute respiratory...
QMW50903.1 (+)	1	VLAAVYRINWITGGIAIAMACLVGLMWLSYFIASFRLFARTRSMWSFNPETNILLNVPLHGTILTRPLLESELVIGAVILRGHLRIAGHHLGRCDIKDLPKEITVATSRTL	222	Severe acute respiratory...
QII87843.1 (+)	1	VLAAIYRINWITGGIAIAMACLVGLMWLSYFIASFRLFARTRSMWSFNPETNILLNVPLHGTILTRPLLESELVIGAVILRGHLRIAGHHLGRCDIKDLPKEITVATSRTL	222	Severe acute respiratory...
UHB05809.1 (+)	1	VLAAVYRINWITGGIAIAMACLVGLMWJSYFIASFRLFARTRSMWSFNPETNILLNVPLHGTILTRPLLESELVIGAVILRGHLRIAGHHLGRCDIKDLPKEITVATSRTL	222	Severe acute respiratory...
QJR91524.1 (+)	1	VLAAVYRINWITGGIAIAMACLVGLMWLSYFIASFRLFARTRSMWSFNPETNILLNVPLHGTILTRPLLESEJVIGAVILRGHLRIAGHHLGRCDIKDLPKEITVATSRTL	222	Severe acute respiratory...
UDY76285.1 (+)	1	VLAAVYRINWITGGIAIAMACLVGLMWLSYFIASFRLFARTRSMWSFNPETNILLNVPLHGTILTRPLLESELVIGAVILRGHLRIAGHHLGRCDIKDLPKEITVATSRTL	222	Severe acute respiratory...
UGW28840.1 (+)	1	VLAAVYRJNWITGGIAIAMACLVGLMWLSYFIASFRLFARTRSMWSFNPETNILLNVPLHGTILTRPLLESELVIGAVILRGHLRIAGHHLGRCDIKDLPKEITVATSRTL	222	Severe acute respiratory...
QTP22999.1 (+)	1	VLAAVYRINWITGGIAIAMACLVGLMWLSYFIASFRLFARTRSMWSFNPETNILLNVPLHGTILTRPLLESELVIGAVILRGHLRIAGHHLGRCDIKDLPKEITVATSRTL	222	Severe acute respiratory...
QXD42760.1 (+)	1	VLAAVYRINWITGGIAIAMACLVGLMWLSYFIASFRLFARTRSMWSFNPETNILLNVPLHGTILTRPLLESELVIGAVILRGHLRIAGHHLGRCDIKDLPKEITVATSRTL	222	Severe acute respiratory...
QOQ27546.1 (+)	1	VLAAVYRINWITGGIAIAMACLVGLMWLSYFIASFKLFARTRSMWSFNPETNILLNVPLHGTILTRPLLESELVIGAVILRGHLRIAGHHLGRCDIKDLPKEITVATSRTL	222	Severe acute respiratory...
QRK20818.1 (+)	1	VLAAVYRINWITGGIAIAMACLVGLMWLSYFIASFRLFARTRSMWSFNPETNILLNVPLHGTILTRPLLESELVIGAVILRGHLRIAGHHLGRCDIKDLPKEITVATSRTL	222	Severe acute respiratory...
QRK16252.1 (+)	1	VLAAVYRINWITGGIAIAMACLVGLMWLSYFIASFRLFARTRSMWSFNPETNILLNVPLHGTILTRPLLESELVIGAVILRGHLRIAGHHLGRCDIKDLPKEITVATSRTL	222	Severe acute respiratory...
UIT02798.1 (+)	1	VLAAVYRINWITGGIAIAMACLVGLMWLSYFIASFRLFARTRSMWSFNPETNILLNVPLHGTILTRPLLESELVIGAVILRGHLRIAGHHLGRCDIKDLPKEITVATSRTL	222	Severe acute respiratory...
QRK18550.1 (+)	1	VLAAVYKINWITGGIAIAMACLVGLMWLSYFIASFRLFARTRSMWSFNPETNILLNVPLHGTILTRPLLESELVIGAVILRGHLRIAGHHLGRCDIKDLPKEITVATSRTL	222	Severe acute respiratory...
UHA86649.1 (+)	1	VLAAVYRINWITGGIAIAMACLVGLMWLSYFIASFRLFARTRSMWSFNPETNILLNVPLHGTILTRPLLESELVIGAVILRGHLRIAGHHLGRCDIKBLPKEITVATSRTL	222	Severe acute respiratory...
QWQ00714.1 (+)	1	VLAAVYRINWITGGIAIAMACLVGLMWLSYFIASFRLFARTRSMWSFNPETNILLNVPLHGTILTRPLLESELVIGAVILRGHLRIAGHHLGRCDIKDLPKEITVATSRTL	222	Severe acute respiratory...
QVI40318.1 (+)	1	VLAAVYRINWITGGIAIAMACLVGLMWLSYFIASFRLFARTRSMWSFNPETNILLNVPLHGTILTRPLLESELVIGAVILRGHLRIAGHHLGRCDIKDLPKEITVATSRTL	222	Severe acute respiratory...
QUN72234.1 (+)	1	VLAAVYRINWITGGIAIAMACLVGLMWLSYFIASFRLFARTRSMWSFNPETNILLNVPLHGTIMTRPLLESELVIGAVILRGHLRIAGHHLGRCDIKDLPKEITVATSRTL	222	Severe acute respiratory...
QUF27701.1 (+)	1	VLAAVYRINWITGGIAIAMACLVGMMWLSYFIASFRLFARTRSMWSFNPETNILLNVPLHGTILTRPLLESELVIGAVILRGHLRIAGHHLGRCDIKDLPKEITVATSRTL	222	Severe acute respiratory...
QUM25742.1 (+)	1	VLAAVYRINWITGGIAIAMACLVGLMWLSYFIASFRLFARTRSMWSFNPETNILLNVPLHGTILTRPLLESELVIGAVILRGHLRIAGHHLGRCDIKDMPKEITVATSRTL	222	Severe acute respiratory...
QRX28625.1 (+)	1	MLAAVYRINWITGGIAIAMACLVGLMWLSYFIASFRLFARTRSMWSFNPETNILLNVPLHGTILTRPLLESELVIGAVILRGHLRIAGHHLGRCDIKDLPKEITVATSRTL	222	Severe acute respiratory...
QYK38286.1 (+)	1	VLAAVYRINWITGGIAIAMACLVGLMWLSYFIASFRLFARTRSMWSFNPETNILLNMPLHGTILTRPLLESELVIGAVILRGHLRIAGHHLGRCDIKDLPKEITVATSRTL	222	Severe acute respiratory...
QRV71062.1 (+)	1	VIAAVYRINWITGGIAIAMACLVGLMWLSYFIASFRLFARTRSMWSFNPETNILLNVPLHGTILTRPLLESELVIGAVILRGHLRIAGHHLGRCDIKDLPKEITVATSRTL	222	Severe acute respiratory...
QOW17180.1 (+)	1	VLAAVYRINWITGGIAIAMACLVGLMWLSYFIASFRLFARTRSMWSFNPETNILLNVPLHGTILTRPLLESELVIGAVILRGHLRIAGHHLGRCDIKDLPKEITVATSRTL	222	Severe acute respiratory...
QRQ69020.1 (+)	1	VLAAVYRINWITGGIAIAMACLVGLMWLSYFIASFRLFARTRSMWSFNPETNILLNVPLHGTILTRPLLESELVIGAVILRGHLRIAGHHLGRCDIKDLPKEITVATSRTL	222	Severe acute respiratory...
QTG31587.1 (+)	1	VLAAVYRINWITGGIAIAMACLVGLMWLSYFIASFRLFARTRSMWSFNPETNILLNVPLHGTILTRPLLESELVIGAVILRGHLRIAGHHLGRCDIKDLPKEITVATSRTL	222	Severe acute respiratory...
QQX34689.1 (+)	1	VLAAVYRINWITGGIAIAMACLVGLMWLSYFIASFRLFARTRSMWSFNPETNILLNVPIHGTILTRPLLESELVIGAVILRGHLRIAGHHLGRCDIKDLPKEITVATSRTL	222	Severe acute respiratory...
QSO08934.1 (+)	1	VLAAVYRINWITGGIAIAMACLVGLMWLSYFIASFRLFARTRSMWSFNPETNILLNVPLHGTILTRPLLESELVIGAVILRGHLRIAGHHLGRCDIKDLPKEITVATSRTL	222	Severe acute respiratory...
QNV70149.1 (+)	1	VLAAVYRINWITGGIAIAMACLVGLMWLSYFIASFRLFARTRSMWSFNPETNILLNVPLHGTILTRPLLESELVIGAVILRGHIRIAGHHLGRCDIKDLPKEITVATSRTL	222	Severe acute respiratory...
QJX70745.1 (+)	1	VLAAVYRINWITGGIAIAMACLVGLMWLSYFIASFRLFARTRSMWSFNPETNILLNVPLHGTILTRPLLESEIVIGAVILRGHLRIAGHHLGRCDIKDLPKEITVATSRTL	222	Severe acute respiratory...
UPC42279.1 (+)	1	VLAAVYRINWITGGIAIAMACLVGLMWLSYFIASFRLFARTRSMWSFNPETNILLNVPLHGTILTRPLLESELVIGAVILRGHLRIAGHHLGRCDIKDLPKEITVATSRTL	222	Severe acute respiratory...
QTA94241.1 (+)	1	VLAAVYRINWITGGIAIAMACIVGLMWLSYFIASFRLFARTRSMWSFNPETNILLNVPLHGTILTRPLLESELVIGAVILRGHLRIAGHHLGRCDIKDLPKEITVATSRTL	222	Severe acute respiratory...
QPD96854.1 (+)	1	VLAAVYRINWITGGIAIAMACLVGLMWISYFIASFRLFARTRSMWSFNPETNILLNVPLHGTILTRPLLESELVIGAVILRGHLRIAGHHLGRCDIKDLPKEITVATSRTL	222	Severe acute respiratory...
URG22635.1 (+)	1	VLAAVYRINWITGGIAIAMACLVGLMWLSYFIASFRLFARTRSMWSFNPETNILLNVPLHGTILTRPLLESELVIGAVILRGHLRIAGHHIGRCDIKDLPKEITVATSRTL	222	Severe acute respiratory...
QVF56053.1 (+)	1	VLAAVYRINWITGGIAIAMACLVGLMWLSYFIASFRLFARTRSMWSFNPETNILLNVPLHGTILTRPLLESELVIGAVILRGHLRIAGHHLGRCDIKDLPKEITVATSRTL	222	Severe acute respiratory...
QWB52244.1 (+)	1	VLAAVYRINWITGGIAIAMACLVGLMWLSYFIASFRLFARTRSMWSFNPETNILLNVPLHGTILTRPLLESELVIGAVIIRGHLRIAGHHLGRCDIKDLPKEITVATSRTL	222	Severe acute respiratory...
QQZ31451.1 (+)	1	VLAAVYRINWITGGIAIAMACLVGLMWLSYFIASFRLFARTRSMWSFNPETNILINVPLHGTILTRPLLESELVIGAVILRGHLRIAGHHLGRCDIKDLPKEITVATSRTL	222	Severe acute respiratory...
QTC59278.1 (+)	1	VLAAVYRINWITGGIAIAMACLVGLMWLSYFIASFRLFARTRSMWSFNPETNILLNVPLHGTILTRPLLESELVIGAVILRGHLRIAGHHLGRCDIKDLPKEITVATSRTL	222	Severe acute respiratory...
QTG43810.1 (+)	1	VLAAVYRINWITGGIAMAMACLVGLMWLSYFIASFRLFARTRSMWSFNPETNILLNVPLHGTILTRPLLESELVIGAVILRGHLRIAGHHLGRCDIKDLPKEITVATSRTL	222	Severe acute respiratory...
QVG46728.1 (+)	1	VLAAVYRINWITGGIAIAMACLVGLMWLSYFIASFRLFARTRSMWSFNPETNILLNVPLHGTILTRPLLESELVIGAVILRGHLRIAGHHLGRCDIKDLPKEITVATSRTL	222	Severe acute respiratory...
QTK18905.1 (+)	1	VLAAVYRINWITGGIAIAMACLVGLMWLSYFIASFRLFARTRSMWSFNPETNILLNVPLHGTILTRPLLESELVIGAVILRGHLRIAGHHLGRCDIKDLPKEITVATSRTL	222	Severe acute respiratory...
QOE87559.1 (+)	1	VLAAVYRINWITGGIAIAMACLVGLMWLSYFIASFRLFARTRSMWSFNPETNILLNVPLHGTILTRPLLESELVIGAVILRGHLRIAGHHLGRCDMKDLPKEITVATSRTL	222	Severe acute respiratory...
QSN96221.1 (+)	1	VLAAVYRMNWITGGIAIAMACLVGLMWLSYFIASFRLFARTRSMWSFNPETNILLNVPLHGTILTRPLLESELVIGAVILRGHLRIAGHHLGRCDIKDLPKEITVATSRTL	222	Severe acute respiratory...
QRX07945.1 (+)	1	VLAAVYRINWITGGIAIAMACLVGLMWLSYFIASFRLYARTRSMWSFNPETNILLNVPLHGTILTRPLLESELVIGAVILRGHLRIAGHHLGRCDIKDLPKEITVATSRTL	222	Severe acute respiratory...
WKJ45353.1 (+)	1	VLAAVYRINWITGGIAIAMACLVGLMWLSYFIASFRLFARTRSMWSFNPETNILLNVPLHGTILTRPLLESELVIGAVILRGHLRIAGHHLGRCDIKDLPKEITVATSRTL	222	Severe acute respiratory...
UGW30145.1 (+)	1	VLAAVYRINWITGGIAIAMACLVGLMWLSYFIASFRLFARTRSMWSYNPETNILLNVPLHGTILTRPLLESELVIGAVILRGHLRIAGHHLGRCDIKDLPKEITVATSRTL	222	Severe acute respiratory...
WNH94212.1 (+)	1	VLAAVYRINWITGGIAIAMACLVGLMWLSYFIASFRLFARTRSMWSFNPETNILLNVPLHGTILTRPLLESELVIGAVILRGHLRIAGHHLGRCDIKDLPKEITVATSRTL	222	Severe acute respiratory...
QRX19824.1 (+)	1	VLAAVYRINWITGGIAIAMACLVGLMWLSYFIASFRLFARTRSMWSFNPETNILLNVPLHGTILTRPLLESELVIGAVILRGHLRIAGHHLGRCDIKDLPKEITVATSRTL	222	Severe acute respiratory...
QSG76325.1 (+)	1	VLAAVYRINWITGGIAIAMACLVGLMWLSYFIASFRLFARTRSMWSFNPETNILLNVPLHGTILTRPLLESELVIGAVILRGHLRIAGHHLGRCDIKDLPKEITVATSRTL	222	Severe acute respiratory...
QRF79522.1 (+)	1	VLAAVYRINWITGGIAIAMACLVGLMWLSYFIASFRLFARTRSMWSFNPETNILLNVPLHGTILTRPLLESELVIGAVILRGHLRIAGHHLGRCDIKDLPKEITVATARTL	222	Severe acute respiratory...
QWV61896.1 (+)	1	VLAAVYRINWITGGIAIAMACLVGLMWLSYFIASFRLFARTRSMWSFNPETNILLNVPLHGTILTRPLLESELVIGAVILRGHLRIAGHHLGRCDIKDLPKEITVATSRTL	222	Severe acute respiratory...
QVN34057.1 (+)	1	VLAAVYRINWITGGIAIAMACLVGLMWLSYFIASFRLFARTRSMWSFNPETNILLNVPLHGTILTRPLLESELVIGAVILRGHLRIAGHHLGRCDIKDLPKEITVATSRTL	222	Severe acute respiratory...
QVM67164.1 (+)	1	VLAAVYRINWITGGIAIAMACLVGLMWLSYFIAAFRLFARTRSMWSFNPETNILLNVPLHGTILTRPLLESELVIGAVILRGHLRIAGHHLGRCDIKDLPKEITVATSRTL	222	Severe acute respiratory...
QTN64908.1 (+)	1	VLAAVYRINWITGGIAIAMACLVGLMWLSYFIASFRLFARTRSMWSFNPETNILLNVPLHGTILTRPLLESELVIGAVILRGHLRIAGHHLGRCDIKDLPKEITVATSRTL	222	Severe acute respiratory...
WQR46117.1 (+)	1	VLAAVYRINWITGGIAIAMACLVGLMWLSYFIASFRLFARTRSMWSFNPETNILLNVPLHGTILTRPLLESELVIGAVILRGHLRIAGHHLGRCDIKDLPKEITVATSRTL	222	Severe acute respiratory...
QVQ63800.1 (+)	1	VLAAVYRINWITGGIAIAMACLVGLMWLSYFIASFRLFARTRSMWSFNPETNILLNVPLHGTILTRPLLESELVIGAVILRGHLRIAGHHLGRCDIKDLPKEITVATSRTL	222	Severe acute respiratory...
QTC72454.1 (+)	1	VLAAVYRINWITGGIAIAMACLVGLMWLSYFIASFRLFARTRSMWSFNPETNILLNVPLHGTILTRPLLESELVIGAVILRGHLRIAGHHLGRCDIKDLPREITVATSRTL	222	Severe acute respiratory...
QVN52091.1 (+)	1	VLAAVYRINWITGGIAIAMACLVGLMWLSYFIASFRLFARTRSMWSFNPETNILLNVPLHGTILTRPLLESELVIGAVILRGHLRIAGHHLGRCDIRDLPKEITVATSRTL	222	Severe acute respiratory...
QKU30584.1 (+)	1	VLAAVYRINWITGGIAIAMACLVGLMWLSYFIASFRLFARTRSMWSFNPETNILLNVPLHGTILTRPLLESELVIGAVILRGHLRIAGHHLGRCDIKDLPKEITVATSRTL	222	Severe acute respiratory...
7VGR_A (+)	1	VLAAVYRINWITGGIAIAMACLVGLMWLSYFIASFRLFARTRSMWSFNPETNILLNVPLHGTILTRPLLESELVIGAVILRGHLRIAGHHLGRCDIKDLPKEITVATSRTL	246	Severe acute respiratory...
QSV96183.1 (+)	1	VLAAVYRINWITGGIAIAMACLVGLMWLSYFIASFRLFARTRSMWSFNPETNILLNVPLHGTILTRPLLESELVIGAVILRGHLRIAGHHLGRCDIQDLPKEITVATSRTL	222	Severe acute respiratory...
QZF22880.1 (+)	1	VLAAVYRINWITGGIAIAMACLVGLMWLSYFIASFRLFARTRSMWSFNPETNILLNVPLHGTILTRPLLESELVIGAVILRGHLRIAGHHLGRCDIKDLPKEITVATSRTL	222	Severe acute respiratory...
QVO86468.1 (+)	1	VLAAVYRINWITGGIAIAMACLVGLMWLSYFIASFRLFARTRSMWSFNPETNILLNVPLHGTILTRPLLESELVIGAVILRGHLRIAGHHLGRCDIKDLPKEITVATSRTL	222	Severe acute respiratory...
QVK10821.1 (+)	1	VLAAVYRINWITGGIAIAMACLVGLMWLSYFIASFRLFARTRSMWSFNPETNILLNVPLHGTILTRPLLESELVIGAVILRGHLRIAGHHLGRCDIKDLPKEITVATSRTL	222	Severe acute respiratory...
QTU36018.1 (+)	1	VLAAVYRINWITGGIAIAMACLVGLMWLSYFIASFRLFARTRSMWSFNPETNILLNVPLHGTILTRPLLESELVIGAVILRGHLRIAGHHLGRCDIKDLPKEITVATSRTL	222	Severe acute respiratory...
QRX09606.1 (+)	1	VLAAVYRINWITGGIAIAMACLVGLMWLTYFIASFRLFARTRSMWSFNPETNILLNVPLHGTILTRPLLESELVIGAVILRGHLRIAGHHLGRCDIKDLPKEITVATSRTL	222	Severe acute respiratory...
UCL48894.1 (+)	1	VLAAVYRINWVTGGIAIAMACLVGLMWLSYFIASFRLFARTRSMWSFNPETNILLNVPLHGTILTRPLLESELVIGAVILRGHLRIAGHHLGRCDIKDLPKEITVATSRTL	222	Severe acute respiratory...
QVI41468.1 (+)	1	VLAAVYRINWLTGGIAIAMACLVGLMWLSYFIASFRLFARTRSMWSFNPETNILLNVPLHGTILTRPLLESELVIGAVILRGHLRIAGHHLGRCDIKDLPKEITVATSRTL	222	Severe acute respiratory...
QOQ03225.1 (+)	1	VLAAVYRINWITGGIAIAMACLVGLMWLSYFIASFRLFARTRSMWSFNPETNILLNVPLHGTILTRPLLESELVIGAVILRGHLRIAGHHLGRCDIKDLPKELTVATSRTL	222	Severe acute respiratory...
QVJ84810.1 (+)	1	VLAAVYRLNWITGGIAIAMACLVGLMWLSYFIASFRLFARTRSMWSFNPETNILLNVPLHGTILTRPLLESELVIGAVILRGHLRIAGHHLGRCDIKDLPKEITVATSRTL	222	Severe acute respiratory...
