# Supplementary material for: Design, Development and Immunogenicity Study of a Multi-Epitope Vaccine Prototype Against SARS-CoV-2
Source: Pharmaceuticals (Basel). 2024 Nov 7;17(11):1498. doi: 10.3390/ph17111498 (PMC11597159; doi:10.3390/ph17111498)
Supplement: Supplementary file 1 [file pharmaceuticals-17-01498-s001.zip › Tables_S1-S7.pdf]

Table S1. Input settings of the molecular docking protocols used in the present study.

Parameter	H2-Db	H2-Kb	I-Ab
X centre	2.782	-14.764	49.226
Y centre	28.595	7.262	32.683
Z centre	5.877	29.778	77.223
X size Å	26	28	40
Y size Å	32	42	36
Z size Å	40	28	40
Energy range	3	3	3
Exhaustiveness	8	8	8
Flexible residues in the protein binding site	5, 7, 9, 22, 24, 33, 34, 45, 59, 62, 63, 65, 66, 68, 70, 72, 73, 74, 76, 77, 80, 81, 84, 95, 97, 99, 114, 116, 118, 123, 124, 142, 143, 146, 147, 149, 150, 155, 156, 159, 160, 163, 167, 168, 171	5, 7, 8, 9, 22, 24, 25, 33, 45, 59, 62, 63, 66, 70, 72, 73, 74, 76, 77, 80, 81, 84, 95, 97, 99, 114, 116, 118, 123, 124, 142, 143, 146, 147, 152, 155, 156, 159, 160, 163, 167, 168, 171	9A, 11A, 22A, 24A, 26A, 31A, 32A, 43A, 50A, 51A, 53A, 54A, 55A, 57A, 61A, 62A, 63A, 65A, 66A, 68A, 69A, 70A, 72A, 73A, 76A, 9B, 11B, 12B, 26B, 28B, 30B, 37B, 47B, 53B, 57B, 60B, 61B, 64B, 67B, 68B, 70B, 71B, 74B, 76B, 77B, 78B, 81B, 82B, 86B, 88B
Peptide flexibility	Rigid backbone	Rigid backbone	Rigid backbone
Peptide anchor positions	p2, p5, p9	p1, p3, p5	p1, p4, p6, p9

Table S2. Quantity of peptides within the datasets used in the present study. The training sets consist of peptides with quantitatively measured affinities. Test sets A – D contain binders measured qualitatively by radioactivity, fluorescence or mass spectrometry. Test set N includes non-binders.

Set	H2-Db<br>Nonamers	H2-Kb<br>Octamers	I-Ab<br>Different length
Training set	690	880	571
Test set A	404	362	188
Test set B	2898	2832	394
Test set C	203	217	168
Test set D	-	296	227
Test set N	133	135	139

Table S3. *Sensitivity* and *specificity* of the best-performing models for peptide binding to H2-Db.

Model	*Sensitivity %*<br>**Test Set A**	*Sensitivity %*<br>**Test Set B**	*Sensitivity %*<br>**Test Set C**	*Specificity %*<br>**Test Set N**
PLS cutoff 5.6	74	83	84	60
RBF cutoff 5.6	74	88	88	71
RF	78	86	86	65

Table S4. PLS-based quantitative matrix (QM) for peptide binding to H2-Db. The free term in the model is 5.649.

ак	p1	p2	p3	p4	p5	p6	p7	p8	p9
Ala	0.015	0.163	-0.068	-0.014	-0.031	-0.023	0.049	-0.023	-0.051
Arg	-0.007	-0.082	-0.107	0.008	-0.081	-0.028	-0.070	-0.047	-0.074
Asn	0.0123	-0.084	-0.008	-0.044	0.338	-0.009	-0.064	-0.010	-0.029
Asp	-0.079	-0.087	-0.086	-0.118	-0.056	0.011	0.148	-0.048	-0.024
Cys	-0.071	-0.082	-0.053	-0.016	-0.056	-0.012	0.014	-0.043	-0.041
Gln	-0.023	0.074	-0.035	0.051	-0.063	0.030	-0.023	-0.054	-
Glu	-0.148	-0.104	-0.081	0.012	-0.080	-0.032	-0.014	0.059	-0.012
Gly	-0.041	0.031	-0.061	-0.098	-0.025	0.055	-0.070	-0.153	-0.015
His	-0.050	-0.090	-0.078	-0.035	0.009	-0.018	0.008	0.058	-
Ile	-0.011	-0.051	0.141	0.072	-0.068	-0.031	0.021	-0.044	0.129
Leu	-0.051	-0.070	0.125	0.051	0.002	0.045	-0.017	0.002	0.067
Lys	0.006	-0.099	-0.123	0.014	-0.087	0.011	-0.115	-0.056	-0.142
Met	-0.018	0.096	0.117	-0.002	0.034	0.023	-0.009	-0.023	0.084
Phe	0.112	-0.032	-0.030	-0.022	-0.112	-0.059	0.049	0.055	-0.015
Pro	-0.102	-0.118	0.049	-0.081	-0.036	-0.041	0.035	0.060	-0.034
Ser	0.083	0.151	-0.122	-0.017	-0.102	-0.024	-0.051	-0.004	-0.070
Thr	0.011	0.051	-0.050	0.043	-0.078	0.038	0.087	0.028	-0.081
Trp	-0.017	-0.066	-0.028	-		0.004	-0.019	0.002	-
Tyr	0.078	-0.078	0.042	-0.055	-0.086	0.043	0.032	0.098	-0.124
Val	0.043	-0.010	0.117	0.141	-0.096	0.011	-0.044	0.044	-0.049

Table S5. *Sensitivity* and *specificity* of the best-performing models for peptide binding to H2-Kb.

Model	*Sensitivity %* **Test Set A**	*Sensitivity %* **Test Set B**	*Sensitivity %* **Test Set C**	*Sensitivity %* **Test Set D**	*Specificity %* **Test Set N**
PLS cutoff 6.2	78	82	88	93	< 50
RBF cutoff 6.2	71	87	89	98	73
RF	77	87	93	94	61

Table S6. PLS-based quantitative matrix (QM) for peptide binding to H2-Kb. The free term in the model is 6.024.

ак	p1	p2	p3	p4	p5	p6	p7	p8
Ala	0.021	0.004	-0.077	0.028	-0.095	0.089	0.031	-0.049
Arg	0.008	-0.074	0.016	0.066	-0.056	0.012	0.046	-0.041
Asn	-0.115	0.050	-0.083	0.013	-0.119	-0.026	0.154	0.013
Asp	-0.091	-0.105	-0.128	-0.006	-0.067	-0.087	-0.052	-0.055
Cys	-0.007	-0.082	-0.009	-0.037	-0.019	0.043	-0.064	0.004
Gln	-0.045	0.067	-0.041	0.029	-0.063	-0.051	0.003	-0.112
Glu	-0.084	-0.114	-0.123	-0.011	-0.085	-0.087	-0.050	-0.045
Gly	-0.107	-0.011	-0.101	-0.082	-0.128	-0.017	0.005	0.047
His	-0.105	0.036	-0.040	-0.066	0.021	0.009	0.042	-0.078
Ile	0.162	0.076	-0.056	-0.001	-0.018	0.080	-0.081	0.203
Leu	-0.015	-0.111	0.085	0.025	-0.090	-0.045	-0.039	-0.075
Lys	-0.037	-0.092	-0.070	0.042	-0.064	-0.042	-0.051	-0.020
Met	0.068	0.024	0.041	-0.005	-0.030	0.001	-0.004	-0.068
Phe	0.021	0.027	0.211	-0.002	0.284	-0.003	0.030	-0.027

Pro	-0.187	-0.077	-0.070	-0.073	-0.086	0.014	0.026	-0.076
Ser	0.118	0.171	-0.177	-0.005	-0.118	0.037	0.020	-0.033
Thr	0.033	0.051	-0.050	-0.006	-0.078	0.012	0.046	-0.099
Trp	0.005	-0.045	0.021	-0.014	-0.039	-0.035	-0.106	0.011
Tyr	-0.003	-0.033	0.259	0.026	0.196	0.067	0.021	-0.049
Val	0.087	-0.012	0.046	0.008	-0.015	0.018	-0.109	-0.041

Table S7. *Sensitivity* and *specificity* of the best-performing model for peptide binding to I-Ab.

**Model**	***Sensitivity %*** **Test Set A**	***Sensitivity %*** **Test Set B**	***Sensitivity %*** **Test Set C**	***Sensitivity %*** **Test Set D**	***Specificity %*** **Test Set N**
RBF cutoff 6.3	100	75	93	100	67
