# Supplementary material for: Design, Development and Immunogenicity Study of a Multi-Epitope Vaccine Prototype Against SARS-CoV-2
Source: Pharmaceuticals (Basel). 2024 Nov 7;17(11):1498. doi: 10.3390/ph17111498 (PMC11597159; doi:10.3390/ph17111498)
Supplement: Supplementary file 1 [file pharmaceuticals-17-01498-s001.zip › Table_S13_S_protein_alignment[490..550].pdf]

NCBI Multiple Sequence Alignment Viewer, Version 1.25.2

Sequence ID	Start	Alignment (490 … 500 … 510 … 520 … 530 … 540 … 550)	End	Organism
BCN86353.1 (+)	1	N G V E G F N C Y F P L Q S Y G F Q P T N G V G Y Q P Y R V V V L S F E L L H A P A T V C G P K K S T N L V K N K C V N F	1,282	Severe acute respiratory...
6XR8_A (+)	1	N G V E G F N C Y F P L Q S Y G F Q P T N G V G Y Q P Y R V V V L S F E L L H A P A T V C G P K K S T N L V K N K C V N F	1,310	Severe acute respiratory...
QJF75467.1 (+)	1	N G V E G F N C Y F P L Q S Y G F Q P T N G V G Y Q P Y R V V V L S F E L L H A P A T V C G P K K S T N L V K N K C V N F	1,273	Severe acute respiratory...
8DT3_C (+)	1	N G V E G F N C Y F P L Q S Y G F Q P T N G V G Y Q P Y R V V V L S F E L L H A P A T V C G P K K S T N L V K N K C V N F	1,281	Severe acute respiratory...
QJX45031.1 (+)	1	N G V E G F N C Y F P L Q S Y G F Q P T N G V G Y Q P Y R V V V L S F E L L H A P A T V C G P K K S T N L V K N K C V N F	1,273	Severe acute respiratory...
QJR85953.1 (+)	1	N G V E G F N C Y F P L Q S Y G F Q P T N G V G Y Q P Y R V V V L S F E L L H A P A T V C G P K K S T N L V K N K C V N F	1,273	Severe acute respiratory...
YP_009724390.1 (+)	1	N G V E G F N C Y F P L Q S Y G F Q P T N G V G Y Q P Y R V V V L S F E L L H A P A T V C G P K K S T N L V K N K C V N F	1,273	Severe acute respiratory...
QRN64146.1 (+)	1	N G V E G F N C Y F P L Q S Y G F Q P T N G V G Y Q P Y R V V V L S F E L L H A P A T V C G P K K S T N L V K N K C V N F	1,273	Severe acute respiratory...
QNN86157.1 (+)	1	N G V E G F N C Y F P L Q S Y G F Q P T N G V G Y Q P Y R V V V L S F E L L H A P A T V C G P K K S T N L V K N K C V N F	1,273	Severe acute respiratory...
QJF77846.1 (+)	1	N G V E G F N C Y F P L Q S Y G F Q P T N G V G Y Q P Y R V V V L S F E L L H A P A T V C G P K K S T N L V K N K C V N F	1,273	Severe acute respiratory...
QRN78371.1 (+)	1	N G V E G F N C Y F P L Q S Y G F Q P T N G V G Y Q P Y R V V V L S F E L L H A P A T V C G P K K S T N L V K N K C V N F	1,273	Severe acute respiratory...
QMS52716.1 (+)	1	N G V E G F N C Y F P L Q S Y G F Q P T N G V G Y Q P H R V V V L S F E L L H A P A T V C G P K K S T N L V K N K C V N F	1,273	Severe acute respiratory...
QIZ16509.1 (+)	1	N G V E G F N C Y F P L Q S Y G F Q P T N G V G Y Q P Y R V V V L S F E L L H A P A T V C G P K K S T N L V K N K C V N F	1,273	Severe acute respiratory...
QMI90807.1 (+)	1	N G V E G F N C Y F P L Q S Y G F Q P T N G V G Y Q P Y R V V V L S F E L L H A P A T V C G P K K S T N L V K N K C V N F	1,273	Severe acute respiratory...
QIZ97039.1 (+)	1	N G V E G F N C Y F P L Q S Y G F Q P T N G V G Y Q P Y R V V V L S F E L L H A P A T V C G P K K S T N L V K N K C V N F	1,273	Severe acute respiratory...
QKS90791.1 (+)	1	N G V E G F N C Y F P L Q S Y G F Q P T N G V G Y Q P Y R V V V L S F E L L H A P A T V C G P K K S T N L V K N K C V N F	1,273	Severe acute respiratory...
QQP45825.1 (+)	1	N G V E G F N C Y F P L Q S Y G F Q P T N G V G Y Q P Y R V V V L S F E L L H A P A T V C G P K K S T N L V K N K C V N F	1,273	Severe acute respiratory...
QMJ01317.1 (+)	1	N G V E G F N C Y F P L Q S Y G F Q P T N G V G Y Q P Y R V V V L S F E L L H A P A T V C G P K K S T N L V K N K C V N F	1,273	Severe acute respiratory...
QOH28993.1 (+)	1	N G V E G F N C Y F P L Q S Y G F Q P T N G V G Y Q P Y R V V V L S F E L L H A P A T V C G P K K S T N L V K N K C V N F	1,273	Severe acute respiratory...
QPN00919.1 (+)	1	N G V E G F N C Y F P L Q S Y G F Q P T N G V G Y Q P Y R V V V L S F E L L H A P A T V C G P K K S T N L V K N K C V N F	1,273	Severe acute respiratory...
QOS49673.1 (+)	1	N G V E G F N C Y F P L Q S Y G F Q P T N G V G Y Q P Y R V V V L S F E L L H A P A T V C G P K K S T N L V K N K C V N F	1,273	Severe acute respiratory...
UJE19031.1 (+)	1	N G V E G F N C Y F P L Q S Y G F Q P T N G V G Y Q P Y R V V V L S F E L L H A P A T V C G P K K S T N L V K N K C V N F	1,273	Severe acute respiratory...
QNN86445.1 (+)	1	N G V E G F N C Y F P L Q S Y G F Q P T N G V G Y Q P Y R V V V L S F E L L H A P A T V C G P K K S T N L V K N K C V N F	1,273	Severe acute respiratory...
QKV39923.1 (+)	1	N G V E G F N C Y F P L Q S Y G F Q P T N G V G Y Q P Y R V V V L S F E L L H A P A T V C G P K K S T N L V K N K C V N F	1,273	Severe acute respiratory...
QNA39510.1 (+)	1	N G V E G F N C Y F P L Q S Y G F Q P T N G V G Y Q P Y R V V V L S F E L L H A P A T V C G P K K S T N L V K N K C V N F	1,273	Severe acute respiratory...
WJZ59893.1 (+)	1	N G V E G F N C Y F P L Q S Y G F Q P T N G V G Y Q P Y R V V V L S F E L L H A P A T V C G P K K S T N L V K N K C V N F	1,273	Severe acute respiratory...
QMX86773.1 (+)	1	N G V E G F N C Y F P L Q S Y G F Q P T N G V G Y Q P Y R V V V L S F E L L H A P A T V C G P K K S T N L V K N K C V N F	1,273	Severe acute respiratory...
QJT73034.1 (+)	1	N G V E G F N C Y F P L Q S Y G F Q P T N G V G Y Q P Y R V V V L S F E L L H A P A T V C G P K K S T N L V K N K C V N F	1,273	Severe acute respiratory...
WQR41098.1 (+)	1	N G V E G F N C Y F P L Q S Y G F Q P T N G V G Y Q P Y R V V V L S F E L L H A P A T V C G P K K S T N L V K N K C V N F	1,273	Severe acute respiratory...
QIZ16559.1 (+)	1	N G V E G F N C Y F P L Q S Y G F Q P T N G V G Y Q P Y R V V V L S F E L L H A P A T V C G P K K S T N L V K N K C V N F	1,273	Severe acute respiratory...
QOF10625.1 (+)	1	N G V E G F N C Y F P L Q S Y G F Q P T N G V G Y Q P Y R V V V L S F E L L H A P A T V C G P K K S T N L V K N K C V N F	1,273	Severe acute respiratory...
QWC76579.1 (+)	1	N G V E G F N C Y F P L Q S Y G F Q P T N G V G Y Q P Y R V V V L S F E L L H A P A T V C G P K K S T N L V K N K C V N F	1,273	Severe acute respiratory...
QSE99577.1 (+)	1	N G V E G F N C Y F P L Q S Y G F Q P T N G V G Y Q P Y R V V V L S F E L L H A P A T V C G P K K S T N L V K N K C V N F	1,273	Severe acute respiratory...
QOF08429.1 (+)	1	N G V E G F N C Y F P L Q S Y G F Q P T N G V G Y Q P Y R V V V L S F E L L H A P A T V C G P K K S T N L V K N K C V N F	1,273	Severe acute respiratory...
QJS39579.1 (+)	1	N G V E G F N C Y F P L Q S Y G F Q P T N G V G Y Q P Y R V V V L S F E L L H A P A T V C G P K K S T N L V K N K C V N F	1,273	Severe acute respiratory...
QOE75686.1 (+)	1	N G V E G F N C Y F P L Q S Y G F Q P T N G V G Y Q P Y R V V V L S F E L L H A P A T V C G P K K S T N L V K N K C V N F	1,273	Severe acute respiratory...
QQO76328.1 (+)	1	N G V E G F N C Y F P L Q S Y G F Q P T N G V G Y Q P Y R V V V L S F E L L H A P A T V C G P K K S T N L V K N K C V N F	1,273	Severe acute respiratory...
UXB96093.1 (+)	1	N G V E G F N C Y F P L Q S Y G F Q P T N G V G Y Q P Y R V V V L S F E L L H A P A T V C G P K K S T N L V K N K C V N F	1,273	Severe acute respiratory...
UYA31504.1 (+)	1	N G V E G F N C Y F P L Q S Y G F Q P T N G V G Y Q P Y R V V V L S F E L L H A P A T V C G P K K S T N L V K N K C V N F	1,273	Severe acute respiratory...
QTI95854.1 (+)	1	N G V E G F N C Y F P L Q S Y G F Q P T N G V G Y Q P Y R V V V L S F E L L H A P A T V C G P K K S T N L V K N K C V N F	1,273	Severe acute respiratory...
QNJ99456.1 (+)	1	N G V E G F N C Y F P L Q S Y G F Q P T N G V G Y Q P Y R V V V L S F E L L H A P A T V C G P K K S T N L V K N K C V N F	1,273	Severe acute respiratory...
QXE71677.1 (+)	1	N G V E G F N C Y F P L Q S Y G F Q P T N G V G Y Q P Y R V V V L S F E L L H A P A T V C G P K K S T N L V K N K C V N F	1,273	Severe acute respiratory...
QIT07011.1 (+)	1	N G V E G F N C Y F P L Q S Y G F Q P T N G V G Y Q P Y R V V V L S F E L L H A P A T V C G P K K S T N L V K N K C V N F	1,273	Severe acute respiratory...
QMX85049.1 (+)	1	N G V E G F N C Y F P L Q S Y G F Q P T N G V G Y Q P Y R V V V L S F E L L H A P A T V C G P K K S T N L V K N K C V N F	1,273	Severe acute respiratory...
QJW70147.1 (+)	1	N G V E G F N C Y F P L Q S Y G F Q P T N G V G Y Q P Y R V V V L S F E L L H A P A T V C G P K K S T N L V K N K C V N F	1,273	Severe acute respiratory...
QKU28894.1 (+)	1	N G V E G F N C Y F P L Q S Y G F Q P T N G V G Y Q P Y R V V V L S F E L L H A P A T V C G P K K S T N L V K N K C V N F	1,273	Severe acute respiratory...
QXR88761.1 (+)	1	N G V E G F N C Y F P L Q S Y G F Q P T N G V G Y Q P Y R V V V L S F E L L H A P A T V C G P K K S T N L V K N K C V N F	1,273	Severe acute respiratory...
QOU94298.1 (+)	1	N G V E G F N C Y F P L Q S Y G F Q P T N G V G Y Q P Y R V V V L S F E L L H A P A T V C G P K K S T N L V K N K C V N F	1,273	Severe acute respiratory...
QJS53398.1 (+)	1	N G V E G F N C Y F P L Q S Y G F Q P T N G V G Y Q P Y R V V V L S F E L L H A P A T V C G P K K S T N L V K N K C V N F	1,273	Severe acute respiratory...
QOU91202.1 (+)	1	N G V E G F N C Y F P L Q S Y G F Q P T N G V G Y Q P Y R V V V L S F E L L H A P A T V C G P K K S T N L V K N K C V N F	1,273	Severe acute respiratory...
QQP47157.1 (+)	1	N G V E G F N C Y F P L Q S Y G F Q P T N G V G Y Q P Y R V V V L S F E L L H A P A T V C G P K K S T N L V K N K C V N F	1,273	Severe acute respiratory...
QLC93524.1 (+)	1	N G V E G F N C Y F P L Q S Y G F Q P T N G V G Y Q P Y R V V V L S F E L L H A P A T V C G P K K S T N L V K N K C V N F	1,273	Severe acute respiratory...
QWM87761.1 (+)	1	N G V E G F N C Y F P L Q S Y G F Q P T N G V G Y Q P Y R V V V L S F E L L H A P A T V C G P K K S T N L V K N K C V N F	1,273	Severe acute respiratory...
QXR89097.1 (+)	1	N G V E G F N C Y F P L Q S Y G F Q P T N G V G Y Q P Y R V V V L S F E L L H A P A T V C G P K K S T N L V K N K C V N F	1,273	Severe acute respiratory...
QOF12353.1 (+)	1	N G V E G F N C Y F P L Q S Y G F Q P T N G V G Y Q P Y R V V V L S F E L L H A P A T V C G P K K S T N L V K N K C V N F	1,273	Severe acute respiratory...
WAQ38474.1 (+)	1	N G V E G F N C Y F P L Q S Y G F Q P T N G V G Y Q P Y R V V V L S F E L L H A P A T V C G P K K S T N L V K N K C V N F	1,273	Severe acute respiratory...
QOU89966.1 (+)	1	N G V E G F N C Y F P L Q S Y G F Q P T N G V G Y Q P Y R V V V L S F E L L H A P A T V C G P K K S T N L V K N K C V N F	1,273	Severe acute respiratory...
QOU93974.1 (+)	1	N G V E G F N C Y F P L Q S Y G F Q P T N G V G Y Q P Y R V V V L S F E L L H A P A T L C G P K K S T N L V K N K C V N F	1,273	Severe acute respiratory...
QQK89979.1 (+)	1	N G V E G F N C Y F P L Q S Y G F Q P T N G V G Y Q P Y R V V V L S F E L L H A P A T V C G P K K S T N L V K N K C V N F	1,273	Severe acute respiratory...
QMI94525.1 (+)	1	N G V Q G F N C Y F P L Q S Y G F Q P T N G V G Y Q P Y R V V V L S F E L L H A P A T V C G P K K S T N L V K N K C V N F	1,273	Severe acute respiratory...
QLC48052.1 (+)	1	N G V E G F N C Y F P L Q S Y G F Q P T N G V G Y Q P Y R V V V L S F E L L H A P A T V C G P K K S T N L V K N K C V N F	1,273	Severe acute respiratory...
QJE38426.1 (+)	1	N G V E G F N C Y F P L Q S Y G F Q P T N G V G Y Q P Y R V V V L S F E L L H A P A T V C G P K K S T N L V K N K C V N F	1,273	Severe acute respiratory...
QIS60489.1 (+)	1	N G V E G F N C Y F P L Q S Y G F Q P T N G V G Y Q P Y R V V V L S F E L L H S P A T V C G P K K S T N L V K N K C V N F	1,273	Severe acute respiratory...
UCK95537.1 (+)	1	N G V E G F N C Y F P L Q S Y G F Q P T N G V G Y Q P Y R V V V L S F E L L H A P A T V C G P K K S T N L V K N K C V N F	1,273	Severe acute respiratory...
QLI51781.1 (+)	1	N G V E G F N C Y F P L Q S Y G F Q P T N G V G Y Q P Y R V V V L S F E L L H A P A T V C G P K K S T N L V K N K C V N F	1,273	Severe acute respiratory...
QKV41471.1 (+)	1	N G V E G F N C Y F P L Q S Y G F Q P T N G V G Y Q P Y R V V V L S F E L L H A P A T V C G P K K S T N L V K N K C V N F	1,273	Severe acute respiratory...
QKY78084.1 (+)	1	N G V E G F N C Y F P L Q S Y G F Q P T N G V G Y Q P Y R V V V L S F E L L H A P A T V C G P K K S T N L V K N K C V N F	1,273	Severe acute respiratory...
UHX58263.1 (+)	1	N G V E G F N C Y F P L Q S Y G F Q P T N G V G Y Q P Y R V V V L S F E L L H A P A T V C G P K K S T N L V K N K C V N F	1,273	Severe acute respiratory...
QRA18933.1 (+)	1	N G V E G F N C Y F P L Q S Y G F Q P T N G V G Y Q P Y R V V V L S F E L L H A P A T V C G P K K S T N L V K N K C V N F	1,273	Severe acute respiratory...
QJR84873.1 (+)	1	N G V E G F N C Y F P L Q S Y G F Q P T N G V G Y Q P Y R V V V L S F E L L H A P A T V C G P K K S T N L V K N K C V N F	1,273	Severe acute respiratory...
QWC77885.1 (+)	1	N G V E G F N C Y F P L Q S Y G F Q P T N G V G Y Q P Y R V V V L S F E L L H A P A T V C G P K K S T N L V K N K C V N F	1,273	Severe acute respiratory...
QRN63738.1 (+)	1	N G V E G F N C Y F P L Q S Y G F Q P T N G V G Y Q P Y R V V V L S F E L L H A P A T V C G P K K S T N L V K N K C V N F	1,273	Severe acute respiratory...
QMT96172.1 (+)	1	N G V E G F N C Y F P L Q S Y G F Q P T N G V G Y Q P Y R V V V L S F E L L H A P A T V C G P K K S T N L V K N K C V N F	1,273	Severe acute respiratory...
QJQ39524.1 (+)	1	N G V E G F N C Y F P L Q S Y G F Q P T N G V G Y Q P Y R V V V L S F E L L H A P A T V C G P K K S T N L V K N K C V N F	1,273	Severe acute respiratory...
QIU80973.1 (+)	1	N G V E G F N C Y F P L Q S Y G F Q P T N G V G Y Q P Y R V V V L S F E L L H A P A T V C G P K K S T N L V K N K C V N F	1,273	Severe acute respiratory...
QOF15989.1 (+)	1	N G V E G F N C Y F P L Q S Y G F Q P T N G V G Y Q P Y R V V V L S F E L L H A P A T V C G P K K S T N L V K N K C V N F	1,273	Severe acute respiratory...
QIS30335.1 (+)	1	N G V E G F N C Y F P L Q S Y G F Q P T N G V G Y Q P Y R V V V L S F E L L H A P A T V C G P K K S T N L V K N K C V N F	1,273	Severe acute respiratory...
QKU28906.1 (+)	1	N G V E G F N C Y F P L Q S Y G F Q P T N G V G Y Q P Y R V V V L S F E L L H A P V T V C G P K K S T N L V K N K C V N F	1,273	Severe acute respiratory...
QRN58518.1 (+)	1	N G V E G F N C Y F P L Q S Y G F Q P T N G V G Y Q P Y R V V V L S F E L L H A P A T V C G P K K S T N L V K N K C V N F	1,273	Severe acute respiratory...
QJQ84676.1 (+)	1	N G V E G F N C Y F P L Q S Y G F Q P T N G V G Y Q P Y R V V V L S F E L L H A P A T V C G P K K S T N L V K N K C V N F	1,273	Severe acute respiratory...
UGP11912.1 (+)	1	N G V E G F N C Y F P L Q S Y G F Q P T N G V G Y Q P Y R V V V L S F E L L H A P A T V C G P K K S T N L V K N K C V N F	1,273	Severe acute respiratory...
QKU33257.1 (+)	1	N G V E G F N C Y F P L Q S Y G F Q P T N G V G Y Q P Y R V V V L S F E L L H A P A T V C G P K K S T N L V K N K C V N F	1,273	Severe acute respiratory...
QJR84837.1 (+)	1	N G V E G F N C Y F P L Q S Y G F Q P T N G V G Y Q P Y R V V V L S F E L L H A P A T V C G P K K S T N L V K N K C V N F	1,273	Severe acute respiratory...
QOU86714.1 (+)	1	N G V E G F N C Y F P L Q S Y G F Q P T N G V G Y Q P Y R V V V L S F E L L H A P A T V C G P K K S T N L V K N K C V N F	1,273	Severe acute respiratory...
QIS60906.1 (+)	1	N G V E G F N C Y F P L Q S Y G F Q P T N G V G Y Q P Y R V V V L S F E L L H A P A T V C G P K K S T N L V K N K C V N F	1,273	Severe acute respiratory...
QKU31481.1 (+)	1	N G V E G F N C Y F P L Q S Y G F Q P T N G V G Y Q P Y R V V V L S F E L L H A P A T V C G P K K S T N L V K N K C V N F	1,273	Severe acute respiratory...
QZJ49063.1 (+)	1	N G V E G F N C Y F P L Q S Y G F Q P T N G V G Y Q P Y R V V V L S F E L L H A P A T V C G P K K S T N L V K N K C V N F	1,273	Severe acute respiratory...
QJC19455.1 (+)	1	N G V E G F N C Y F P L Q S Y G F Q P T N G V G Y Q P Y R V V V L S F E L L H A P A T V C G P K K S T N L V K N K C V N F	1,273	Severe acute respiratory...
QQP44733.1 (+)	1	N G V E G F N C Y F P L Q S Y G F Q P T N G V G Y Q P Y R V V V L S F E L L H A P A T V C G P K K S T N L V K N K C V N F	1,273	Severe acute respiratory...
QWJ89677.1 (+)	1	N G V E G F N C Y F P L Q S Y G F Q P T N G V G Y Q P Y R V V V L S F E L L H A P A T V C G P K K S T N L V K N K C V N F	1,273	Severe acute respiratory...
QKV35819.1 (+)	1	N G V E G F N C Y F P L Q S Y G F Q P T N G V G Y Q P Y R V V V L S F E L L H A P A T V C G P K K S T N L V K N K C V N F	1,273	Severe acute respiratory...
QPF48560.1 (+)	1	N G V E G F N C Y F P L Q S Y G F Q P T N G V G Y Q P Y R V V V L S F E L L H A P A T V C G P K K S T N L V K N K C V N F	1,273	Severe acute respiratory...
UFV35855.1 (+)	1	N G V E G F N C Y F P L Q S Y G F Q P T N G V G Y Q P Y R V V V L S F E L L H A P A T V C G P K K S T N L V K N K C V N F	1,273	Severe acute respiratory...
QIA98583.1 (+)	1	N G V E G F N C Y F P L Q S Y G F Q P T N G V G Y Q P Y R V V V L S F E L L H A P A T V C G P K K S T N L V K N K C V N F	1,273	Severe acute respiratory...
UHH97155.1 (+)	1	N G V E G F N C Y F P L Q S Y G F Q P T N G V G Y Q P Y R V V V L S F E L L H A P A T V C G P K K S T N L V K N K C V N F	1,273	Severe acute respiratory...
QIS30165.1 (+)	1	N G A E G F N C Y F P L Q S Y G F Q P T N G V G Y Q P Y R V V V L S F E L L H A P A T V C G P K K S T N L V K N K C V N F	1,273	Severe acute respiratory...
QLJ53548.1 (+)	1	N G V E G F N C Y F P L Q S Y G F Q P T N G V G Y Q P Y R V V V L S F E L L H A P A T V C G P K K S T N L V K N K C V N F	1,273	Severe acute respiratory...
QJW39952.1 (+)	1	N G V E G F N C Y F P L Q S Y G F Q P T N G V G Y Q P Y R V V V L S F E L L H A P A T V C G P K K S T N L V K N K C V N F	1,273	Severe acute respiratory...
QIU81873.2 (+)	1	N G V E G F N C Y F P L Q S Y G F Q P T N G V G Y Q P Y R V V V L S F E L L H A P A T V C G P K K S T N L V K N K C V N F	1,273	Severe acute respiratory...
QOU93902.1 (+)	1	N G V E G F N C Y F P L Q S Y G F Q P T N G V G Y Q P Y R V V V L S F E L L H A P A T V C G P K K S T N L V K N K C V N F	1,273	Severe acute respiratory...
