# Supplementary material for: Design, Development and Immunogenicity Study of a Multi-Epitope Vaccine Prototype Against SARS-CoV-2
Source: Pharmaceuticals (Basel). 2024 Nov 7;17(11):1498. doi: 10.3390/ph17111498 (PMC11597159; doi:10.3390/ph17111498)
Supplement: Supplementary file 1 [file pharmaceuticals-17-01498-s001.zip › Table_S14_S_protein_alignment[890..920].pdf]

NCBI Multiple Sequence Alignment Viewer, Version 1.25.2

Sequence ID	Start	Alignment																															End	Organism
		890		892	893	894	895	896	897	898	899	900	901	902	903	904	905	906	907	908	909	910	911	912	913	914	915	916	917	918	919	920		
BCN86353.1 (+)	1	T	I	T	S	G	W	T	F	G	A	G	A	A	L	Q	I	P	F	A	M	Q	M	A	Y	R	F	N	G	I	G	V	1,282	Severe acute respiratory...
6XR8_A (+)	1	T	I	T	S	G	W	T	F	G	A	G	A	A	L	Q	I	P	F	A	M	Q	M	A	Y	R	F	N	G	I	G	V	1,310	Severe acute respiratory...
QJF75467.1 (+)	1	T	I	T	S	G	W	T	F	G	A	G	A	A	L	Q	I	P	F	A	M	Q	M	A	Y	R	F	N	G	I	G	V	1,273	Severe acute respiratory...
8DT3_C (+)	1	T	I	T	S	G	W	T	F	G	A	G	A	A	L	Q	I	P	F	A	M	Q	M	A	Y	R	F	N	G	I	G	V	1,281	Severe acute respiratory...
QJX45031.1 (+)	1	T	I	T	S	G	W	T	F	G	A	G	A	A	L	Q	I	P	F	A	M	Q	M	A	Y	R	F	N	G	I	G	V	1,273	Severe acute respiratory...
QJR85953.1 (+)	1	T	I	T	S	G	W	T	F	G	A	G	A	A	L	Q	I	P	F	A	M	Q	M	A	Y	R	F	N	G	I	G	V	1,273	Severe acute respiratory...
YP_009724390.1 (+)	1	T	I	T	S	G	W	T	F	G	A	G	A	A	L	Q	I	P	F	A	M	Q	M	A	Y	R	F	N	G	I	G	V	1,273	Severe acute respiratory...
QRN64146.1 (+)	1	T	I	T	S	G	W	T	F	G	A	G	A	A	L	Q	I	P	F	A	M	Q	M	A	Y	R	F	N	G	I	G	V	1,273	Severe acute respiratory...
QNN86157.1 (+)	1	T	I	T	S	G	W	T	F	G	A	G	A	A	L	Q	I	P	F	A	M	Q	M	A	Y	R	F	N	G	I	G	V	1,273	Severe acute respiratory...
QJF77846.1 (+)	1	T	I	T	S	G	W	T	F	G	A	G	A	A	L	Q	I	P	F	A	M	Q	M	A	Y	R	F	N	G	I	G	V	1,273	Severe acute respiratory...
QRN78371.1 (+)	1	T	I	T	S	G	W	T	F	G	A	G	A	A	L	Q	I	P	F	A	M	Q	M	A	Y	R	F	N	G	I	G	V	1,273	Severe acute respiratory...
QMS52716.1 (+)	1	T	I	T	S	G	W	T	F	G	A	G	A	A	L	Q	I	P	F	A	M	Q	M	A	Y	R	F	N	G	I	G	V	1,273	Severe acute respiratory...
QIZ16509.1 (+)	1	T	I	T	S	G	W	T	F	G	A	G	A	A	L	Q	I	P	F	A	M	Q	M	A	Y	R	F	N	G	I	G	V	1,273	Severe acute respiratory...
QMI90807.1 (+)	1	T	I	T	S	G	W	T	F	G	A	G	A	A	L	Q	I	P	F	A	M	Q	M	A	Y	R	F	N	G	I	G	V	1,273	Severe acute respiratory...
QIZ97039.1 (+)	1	T	I	T	S	G	W	T	F	G	A	G	A	A	L	Q	I	P	F	A	M	Q	M	A	Y	R	F	N	G	I	G	V	1,273	Severe acute respiratory...
QKS90791.1 (+)	1	T	I	T	S	G	W	T	F	G	A	G	A	A	L	Q	I	P	F	A	M	Q	M	A	Y	R	F	N	G	I	G	V	1,273	Severe acute respiratory...
QQP45825.1 (+)	1	T	I	T	S	G	W	T	F	G	A	G	A	A	L	Q	I	P	F	A	M	Q	M	A	Y	R	F	N	G	I	G	V	1,273	Severe acute respiratory...
QMJ01317.1 (+)	1	T	I	T	S	G	W	T	F	G	A	G	A	A	L	Q	I	P	F	A	M	Q	M	A	Y	R	F	N	G	I	G	V	1,273	Severe acute respiratory...
QOH28993.1 (+)	1	T	I	T	S	G	W	T	F	G	A	G	A	A	L	Q	I	P	F	A	M	Q	M	A	Y	R	F	N	G	I	G	V	1,273	Severe acute respiratory...
QPN00919.1 (+)	1	T	I	T	S	G	W	T	F	G	A	G	A	A	L	Q	I	P	F	A	M	Q	M	A	Y	R	F	N	G	I	G	V	1,273	Severe acute respiratory...
QOS49673.1 (+)	1	T	I	T	S	G	W	T	F	G	A	G	A	A	L	Q	I	P	F	A	M	Q	M	A	Y	R	F	N	G	I	G	V	1,273	Severe acute respiratory...
UJE19031.1 (+)	1	T	I	T	S	G	W	T	F	G	A	G	A	A	L	Q	I	P	F	A	M	Q	M	A	Y	R	F	N	G	I	G	V	1,273	Severe acute respiratory...
QNN86445.1 (+)	1	T	I	T	S	G	W	T	F	G	A	G	A	A	L	Q	I	P	F	A	M	Q	M	A	Y	R	F	N	G	I	G	V	1,273	Severe acute respiratory...
QKV39923.1 (+)	1	T	I	T	S	G	W	T	F	G	A	G	A	A	L	Q	I	P	F	A	M	Q	M	A	Y	R	F	N	G	I	G	V	1,273	Severe acute respiratory...
QNA39510.1 (+)	1	T	I	T	S	G	W	T	F	G	A	G	A	A	L	Q	I	P	F	A	M	Q	M	A	Y	R	F	N	G	I	G	V	1,273	Severe acute respiratory...
WJZ59893.1 (+)	1	T	I	T	S	G	W	T	F	G	A	G	A	A	L	Q	I	P	F	A	M	Q	M	A	Y	R	F	N	G	I	G	V	1,273	Severe acute respiratory...
QMX86773.1 (+)	1	T	I	T	S	G	W	T	F	G	A	G	A	A	L	Q	I	P	F	A	M	Q	M	A	Y	R	F	N	G	I	G	V	1,273	Severe acute respiratory...
QJT73034.1 (+)	1	T	I	T	S	G	W	T	F	G	A	G	A	A	L	Q	I	P	F	A	M	Q	M	A	Y	R	F	N	G	I	G	V	1,273	Severe acute respiratory...
WQR41098.1 (+)	1	T	I	T	S	G	W	T	F	G	A	G	A	A	L	Q	I	P	F	A	M	Q	M	A	Y	R	F	N	G	I	G	V	1,273	Severe acute respiratory...
QIZ16559.1 (+)	1	T	I	T	S	G	W	T	F	G	A	G	A	A	L	Q	I	P	F	A	M	Q	M	A	Y	R	F	N	G	I	G	V	1,273	Severe acute respiratory...
QOF10625.1 (+)	1	T	I	T	S	G	W	T	F	G	A	G	A	A	L	Q	I	P	F	A	M	Q	M	A	Y	R	F	N	G	I	G	V	1,273	Severe acute respiratory...
QWC76579.1 (+)	1	T	I	T	S	G	W	T	F	G	A	G	A	A	L	Q	I	P	F	A	M	Q	M	A	Y	R	F	N	G	I	G	V	1,273	Severe acute respiratory...
QSE99577.1 (+)	1	T	I	T	S	G	W	T	F	G	A	G	A	A	L	Q	I	P	F	A	M	Q	M	A	Y	R	F	N	G	I	G	V	1,273	Severe acute respiratory...
QOF08429.1 (+)	1	T	I	T	S	G	W	T	F	G	A	G	A	A	L	Q	I	P	F	A	M	Q	M	A	Y	R	F	N	G	I	G	V	1,273	Severe acute respiratory...
QJS39579.1 (+)	1	T	I	T	S	G	W	T	F	G	A	G	A	A	L	Q	I	P	F	A	M	Q	M	A	Y	R	F	N	G	I	G	V	1,273	Severe acute respiratory...
QOE75686.1 (+)	1	T	I	T	S	G	W	T	F	G	A	G	A	A	L	Q	I	P	F	A	M	Q	M	A	Y	R	F	N	G	I	G	V	1,273	Severe acute respiratory...
QQO76328.1 (+)	1	T	I	T	S	G	W	T	F	G	A	G	A	A	L	Q	I	P	F	A	M	Q	M	A	Y	R	F	N	G	I	G	V	1,273	Severe acute respiratory...
UXB96093.1 (+)	1	T	I	T	S	G	W	T	F	G	A	G	A	A	L	Q	I	P	F	A	M	Q	M	A	Y	R	F	N	G	I	G	V	1,273	Severe acute respiratory...
UYA31504.1 (+)	1	T	I	T	S	G	W	T	F	G	A	G	A	A	L	Q	I	P	F	A	M	Q	M	A	Y	R	F	N	G	I	G	V	1,273	Severe acute respiratory...
QTI95854.1 (+)	1	T	I	T	S	G	W	T	F	G	A	G	A	A	L	Q	I	P	F	A	M	Q	M	A	Y	R	F	N	G	I	G	V	1,273	Severe acute respiratory...
QNJ99456.1 (+)	1	T	I	T	S	G	W	T	F	G	A	G	A	A	L	Q	I	P	F	A	M	Q	M	A	Y	R	F	N	G	I	G	V	1,273	Severe acute respiratory...
QXE71677.1 (+)	1	T	I	T	S	G	W	T	F	G	A	G	A	A	L	Q	I	P	F	A	M	Q	M	A	Y	R	F	N	G	I	G	V	1,273	Severe acute respiratory...
QIT07011.1 (+)	1	T	I	T	S	G	W	T	F	G	A	G	A	A	L	Q	I	P	F	A	M	Q	M	A	Y	R	F	N	G	I	G	V	1,273	Severe acute respiratory...
QMX85049.1 (+)	1	T	I	T	S	G	W	T	F	G	A	G	A	A	L	Q	I	P	F	A	M	Q	M	A	Y	R	F	N	G	I	G	V	1,273	Severe acute respiratory...
QJW70147.1 (+)	1	T	I	T	S	G	W	T	F	G	A	G	A	A	L	Q	I	P	F	A	M	Q	M	A	Y	R	F	N	G	I	G	V	1,273	Severe acute respiratory...
QKU28894.1 (+)	1	T	I	T	S	G	W	T	F	G	A	G	A	A	L	Q	I	P	F	A	M	Q	M	A	Y	R	F	N	G	I	G	V	1,273	Severe acute respiratory...
QXR88761.1 (+)	1	T	I	T	S	G	W	T	F	G	A	G	A	A	L	Q	I	P	F	A	M	Q	M	A	Y	R	F	N	G	I	G	V	1,273	Severe acute respiratory...
QOU94298.1 (+)	1	T	I	T	S	G	W	T	F	G	A	G	A	A	L	Q	I	P	F	A	M	Q	M	A	Y	R	F	N	G	I	G	V	1,273	Severe acute respiratory...
QJS53398.1 (+)	1	T	I	T	S	G	W	T	F	G	A	G	A	A	L	Q	I	P	F	A	M	Q	M	A	Y	R	F	N	G	I	G	V	1,273	Severe acute respiratory...
QOU91202.1 (+)	1	T	I	T	S	G	W	T	F	G	A	G	A	A	L	Q	I	P	F	A	M	Q	M	A	Y	R	F	N	G	I	G	V	1,273	Severe acute respiratory...
QQP47157.1 (+)	1	T	I	T	S	G	W	T	F	G	A	G	A	A	L	Q	I	P	F	A	M	Q	M	A	Y	R	F	N	G	I	G	V	1,273	Severe acute respiratory...
QLC93524.1 (+)	1	T	I	T	S	G	W	T	F	G	A	G	A	A	L	Q	I	P	F	A	M	Q	M	A	Y	R	F	N	G	I	G	V	1,273	Severe acute respiratory...
QWM87761.1 (+)	1	T	I	T	S	G	W	T	F	G	A	G	A	A	L	Q	I	P	F	A	M	Q	M	A	Y	R	F	N	G	I	G	V	1,273	Severe acute respiratory...
QXR89097.1 (+)	1	T	I	T	S	G	W	T	F	G	A	G	A	A	L	Q	I	P	F	A	M	Q	M	A	Y	R	F	N	G	I	G	V	1,273	Severe acute respiratory...
QOF12353.1 (+)	1	T	I	T	S	G	W	T	F	G	A	G	A	A	L	Q	I	P	F	A	M	Q	M	A	Y	R	F	N	G	I	G	V	1,273	Severe acute respiratory...
WAQ38474.1 (+)	1	T	I	T	S	G	W	T	F	G	A	G	A	A	L	Q	I	P	F	A	M	Q	M	A	Y	R	F	N	G	I	G	V	1,273	Severe acute respiratory...
QOU89966.1 (+)	1	T	I	T	S	G	W	T	F	G	A	G	A	A	L	Q	I	P	F	A	M	Q	M	A	Y	R	F	N	G	I	G	V	1,273	Severe acute respiratory...
QOU93974.1 (+)	1	T	I	T	S	G	W	T	F	G	A	G	A	A	L	Q	I	P	F	A	M	Q	M	A	Y	R	F	N	G	I	G	V	1,273	Severe acute respiratory...
QQK89979.1 (+)	1	T	I	T	S	G	W	T	F	G	A	G	A	A	L	Q	I	P	F	A	M	Q	M	A	Y	R	F	N	G	I	G	V	1,273	Severe acute respiratory...
QMI94525.1 (+)	1	T	I	T	S	G	W	T	F	G	A	G	A	A	L	Q	I	P	F	A	M	Q	M	A	Y	R	F	N	G	I	G	V	1,273	Severe acute respiratory...
QLC48052.1 (+)	1	T	I	T	S	G	W	T	F	G	A	G	A	A	L	Q	I	P	F	A	M	Q	M	A	Y	R	F	N	G	I	G	V	1,273	Severe acute respiratory...
QJE38426.1 (+)	1	T	I	T	S	G	W	T	F	G	A	G	A	A	L	Q	I	P	F	A	M	Q	M	A	Y	R	F	N	G	I	G	V	1,273	Severe acute respiratory...
QIS60489.1 (+)	1	T	I	T	S	G	W	T	F	G	A	G	A	A	L	Q	I	P	F	A	M	Q	M	A	Y	R	F	N	G	I	G	V	1,273	Severe acute respiratory...
UCK95537.1 (+)	1	T	I	T	S	G	W	T	F	G	A	G	A	A	L	Q	I	P	F	A	M	Q	M	A	Y	R	F	N	G	I	G	V	1,273	Severe acute respiratory...
QLI51781.1 (+)	1	T	I	T	S	G	W	T	F	G	A	G	A	A	L	Q	I	P	F	A	M	Q	M	A	Y	R	F	N	G	I	G	V	1,273	Severe acute respiratory...
QKV41471.1 (+)	1	T	I	T	S	G	W	T	F	G	A	G	A	A	L	Q	I	P	F	A	M	Q	M	A	Y	R	F	N	G	I	G	V	1,273	Severe acute respiratory...
QKY78084.1 (+)	1	T	I	T	S	G	W	T	F	G	A	G	A	A	L	Q	I	P	F	A	M	Q	M	A	Y	R	F	N	G	I	G	V	1,273	Severe acute respiratory...
UHX58263.1 (+)	1	T	I	T	S	G	W	T	F	G	A	G	A	A	L	Q	I	P	F	A	M	Q	M	A	Y	R	F	N	G	I	G	V	1,273	Severe acute respiratory...
QRA18933.1 (+)	1	T	I	T	S	G	W	T	F	G	A	G	A	A	L	Q	I	P	F	A	M	Q	M	A	Y	R	F	N	G	I	G	V	1,273	Severe acute respiratory...
QJR84873.1 (+)	1	T	I	T	S	G	W	T	F	G	A	G	A	A	L	Q	I	P	F	A	M	Q	M	A	Y	R	F	N	G	I	G	V	1,273	Severe acute respiratory...
QWC77885.1 (+)	1	T	I	T	S	G	W	T	F	G	A	G	A	A	L	Q	I	P	F	A	M	Q	M	A	Y	R	F	N	G	I	G	V	1,273	Severe acute respiratory...
QRN63738.1 (+)	1	T	I	T	S	G	W	T	F	G	A	G	A	A	L	Q	I	P	F	A	M	Q	M	A	Y	R	F	N	G	I	G	V	1,273	Severe acute respiratory...
QMT96172.1 (+)	1	T	I	T	S	G	W	T	F	G	A	G	A	V	L	Q	I	P	F	A	M	Q	M	A	Y	R	F	N	G	I	G	V	1,273	Severe acute respiratory...
QJQ39524.1 (+)	1	T	I	T	S	G	W	T	F	G	A	G	A	A	L	Q	I	P	F	A	M	Q	M	A	Y	R	F	N	G	I	G	V	1,273	Severe acute respiratory...
QIU80973.1 (+)	1	T	I	T	S	G	W	T	F	G	A	G	A	A	L	Q	I	P	F	A	M	Q	M	A	Y	R	F	N	G	I	G	V	1,273	Severe acute respiratory...
QOF15989.1 (+)	1	T	I	T	S	G	W	T	F	G	A	G	A	A	L	Q	I	P	F	A	M	Q	M	A	Y	R	F	N	G	I	G	V	1,273	Severe acute respiratory...
QIS30335.1 (+)	1	T	I	T	S	G	W	T	F	G	A	G	A	A	L	Q	I	P	F	A	M	Q	M	A	Y	R	F	N	G	I	G	V	1,273	Severe acute respiratory...
QKU28906.1 (+)	1	T	I	T	S	G	W	T	F	G	A	G	A	A	L	Q	I	P	F	A	M	Q	M	A	Y	R	F	N	G	I	G	V	1,273	Severe acute respiratory...
QRN58518.1 (+)	1	T	I	T	S	G	W	T	F	G	A	G	A	A	L	Q	I	P	F	A	M	Q	M	A	Y	R	F	N	G	I	G	V	1,273	Severe acute respiratory...
QJQ84676.1 (+)	1	T	I	T	S	G	W	T	F	G	A	G	A	A	L	Q	I	P	F	A	M	Q	M	A	Y	R	F	N	G	I	G	V	1,273	Severe acute respiratory...
UGP11912.1 (+)	1	T	I	T	S	G	W	T	F	G	A	G	A	A	L	Q	I	P	F	A	M	Q	M	A	Y	R	F	N	G	I	G	V	1,273	Severe acute respiratory...
QKU33257.1 (+)	1	T	I	T	S	G	W	T	F	G	A	G	A	A	L	Q	I	P	F	A	M	Q	M	A	Y	R	F	N	G	I	G	V	1,273	Severe acute respiratory...
QJR84837.1 (+)	1	T	I	T	S	G	W	T	F	G	A	G	A	A	L	Q	I	P	F	A	M	Q	M	A	Y	R	F	N	G	I	G	V	1,273	Severe acute respiratory...
QOU86714.1 (+)	1	T	I	T	S	G	W	T	F	G	A	G	V	A	L	Q	I	P	F	A	M	Q	M	A	Y	R	F	N	G	I	G	V	1,273	Severe acute respiratory...
QIS60906.1 (+)	1	T	I	T	S	G	W	T	F	G	A	G	A	A	L	Q	I	P	F	A	M	Q	M	A	Y	R	F	N	G	I	G	V	1,273	Severe acute respiratory...
QKU31481.1 (+)	1	T	I	T	X	G	W	T	F	G	A	G	A	A	L	Q	I	P	F	A	M	Q	M	A	Y	R	F	N	G	I	G	V	1,273	Severe acute respiratory...
QZJ49063.1 (+)	1	T	I	T	S	G	W	T	F	G	A	G	A	A	L	Q	I	P	F	A	M	Q	M	A	Y	R	F	N	G	I	G	V	1,273	Severe acute respiratory...
QJC19455.1 (+)	1	T	I	T	S	G	W	T	F	G	A	G	A	A	L	Q	I	P	F	A	M	Q	M	A	Y	R	F	N	G	I	G	V	1,273	Severe acute respiratory...
QQP44733.1 (+)	1	T	I	T	S	G	W	T	F	G	A	G	A	A	L	Q	I	P	F	A	M	Q	M	A	Y	R	F	N	G	I	G	V	1,273	Severe acute respiratory...
QWJ89677.1 (+)	1	T	I	T	S	G	W	T	F	G	A	G	A	A	L	Q	I	P	F	A	M	Q	M	A	Y	R	F	N	G	I	G	V	1,273	Severe acute respiratory...
QKV35819.1 (+)	1	T	I	T	S	G	W	T	F	G	A	G	A	A	L	Q	I	P	F	A	M	Q	M	A	Y	R	F	N	G	I	G	V	1,273	Severe acute respiratory...
QPF48560.1 (+)	1	T	I	T	S	G	W	T	F	G	A	G	A	A	L	Q	I	P	F	A	M	Q	M	A	Y	R	F	N	G	I	G	V	1,273	Severe acute respiratory...
UFV35855.1 (+)	1	T	I	T	S	G	W	T	F	G	A	G	A	A	L	Q	I	P	F	A	M	Q	M	A	Y	R	F	N	G	I	G	V	1,273	Severe acute respiratory...
QIA98583.1 (+)	1	T	I	T	S	G	W	T	F	G	A	G	A	A	L	Q	I	P	F	A	M	Q	M	A	Y	R	F	N	G	I	G	V	1,273	Severe acute respiratory...
UHH97155.1 (+)	1	T	I	T	S	G	W	T	F	G	A	G	A	A	L	Q	I	P	F	A	M	Q	M	A	Y	R	F	N	G	I	G	V	1,273	Severe acute respiratory...
QIS30165.1 (+)	1	T	I	T	S	G	W	T	F	G	A	G	A	A	L	Q	I	P	F	A	M	Q	M	A	Y	R	F	N	G	I	G	V	1,273	Severe acute respiratory...
QLJ53548.1 (+)	1	T	I	T	S	G	W	T	F	G	A	G	A	A	L	Q	I	P	F	A	M	Q	M	A	Y	R	F	N	G	I	G	V	1,273	Severe acute respiratory...
QJW39952.1 (+)	1	T	I	T	S	G	W	T	F	G	A	G	A	A	L	Q	I	P	F	A	M	Q	M	A	Y	R	F	N	G	I	G	V	1,273	Severe acute respiratory...
QIU81873.2 (+)	1	T	I	T	S	G	W	T	F	G	A	G	A	A	L	Q	I	P	F	A	M	Q	M	A	Y	R	F	N	G	I	G	V	1,273	Severe acute respiratory...
QOU93902.1 (+)	1	T	I	T	S	G	W	T	F	G	V	G	A	A	L	Q	I	P	F	A	M	Q	M	A	Y	R	F	N	G	I	G	V	1,273	Severe acute respiratory...
